# Discovery of a Myeloid Cell Leukemia 1 (Mcl-1) Inhibitor That Demonstrates Potent *In Vivo* Activities in Mouse Models of Hematological and Solid Tumors

**DOI:** 10.1021/acs.jmedchem.4c01188

**Published:** 2024-08-05

**Authors:** James C. Tarr, James M. Salovich, Martin Aichinger, KyuOk Jeon, Nagarathanam Veerasamy, John L. Sensintaffar, Heribert Arnhof, Matthias Samwer, Plamen P. Christov, Kwangho Kim, Tobias Wunberg, Norbert Schweifer, Francesca Trapani, Allison Arnold, Florian Martin, Bin Zhao, Nagaraju Miriyala, Danielle Sgubin, Stuart Fogarty, William J. Moore, Gordon M. Stott, Edward T. Olejniczak, Harald Engelhardt, Dorothea Rudolph, Taekyu Lee, Darryl B. McConnell, Stephen W. Fesik

**Affiliations:** † Department of Biochemistry, Vanderbilt University School of Medicine, 2215 Garland Avenue, 607 Light Hall, Nashville, Tennessee 37232-0146, United States; ‡ Discovery Research, Boehringer Ingelheim Regional Center Vienna GmbH & Co KG, 1120 Vienna, Austria; § Molecular Design and Synthesis Center, Vanderbilt Institute of Chemical Biology, Vanderbilt University, Nashville, Tennessee 37323-0146, United States; ∥ Leidos Biomedical Research, Frederick National Laboratory for Cancer Research, Frederick, Maryland 21701-4907, United States

## Abstract

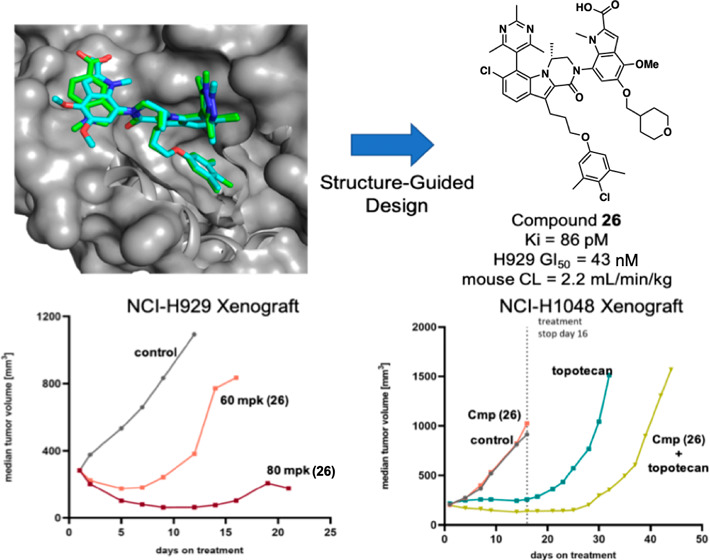

Myeloid cell leukemia 1 (Mcl-1) is a key regulator of the intrinsic apoptosis pathway. Overexpression of Mcl-1 is correlated with high tumor grade, poor survival, and both intrinsic and acquired resistance to cancer therapies. Herein, we disclose the structure-guided design of a small molecule Mcl-1 inhibitor, compound **26**, that binds to Mcl-1 with subnanomolar affinity, inhibits growth in cell culture assays, and possesses low clearance in mouse and dog pharmacokinetic (PK) experiments. Evaluation of **26** as a single agent in Mcl-1 sensitive hematological and solid tumor xenograft models resulted in regressions. Co-treatment of Mcl-1-sensitive and Mcl-1 insensitive lung cancer derived xenografts with **26** and docetaxel or topotecan, respectively, resulted in an enhanced tumor response. These findings support the premise that pro-apoptotic priming of tumor cells by other therapies in combination with Mcl-1 inhibition may significantly expand the subset of cancers in which Mcl-1 inhibitors may prove beneficial.

## Introduction

The B-cell lymphoma 2 (Bcl-2) family of proteins plays a crucial role in regulating the intrinsic apoptotic pathway.^1−3^ Evasion of cell death is one of the hallmarks of cancer and frequently associated with dysregulation of the Bcl-2 family of proteins, which is comprised of effector proteins (BAK, BAX), BH3-only pro-apoptotic proteins (Bim, Bid, Bad, PUMA, NOXA), and antiapoptotic proteins (Bcl-2, Bcl-xL, Bcl-W, Bcl-A1, myeloid cell leukemia 1 (Mcl-1)).^4−8^ In normal cells, the anti-apoptotic family members bind to the pro-apoptotic proteins and the effector proteins of the family to prevent initiation of the programmed cell death process. When stress signals or death stimuli are received, upregulation of the pro-apoptotic members can occur, causing release of the BAK/BAX effector proteins. The unbound BAK/BAX proteins can then oligomerize and permeabilize the outer mitochondrial membrane, releasing cytochrome *c* and initiating the caspase cascade. However, increased expression of the anti-apoptotic family members can sequester the BH3-only pro-apoptotic members and thus allow a cell to evade apoptosis even in the presence of a death signal.^1,2,6,7^ Therefore, inhibition of anti-apoptotic Bcl-2 proteins can restore the normal intrinsic apoptotic pathway in cancer cells, as evidenced by the FDA approved selective Bcl-2 inhibitor Venetoclax (ABT-199) for the treatment of chronic lymphocytic leukemia.^9−11^

Anti-apoptotic Bcl-2 family member Mcl-1 has emerged as a target of significant interest for the treatment of cancer. Amplification of the *MCL1* gene is one of the most common genetic changes observed in cancer,^12−16^ and overexpression of the Mcl-1 protein has been observed in both hematologic cancers (leukemia, lymphoma, myeloma)^17−19^ as well as solid tumors (lung, breast, pancreatic, cervical, and ovarian).^20−24^ Mcl-1 plays a key role in tumor development, as overexpression of *Mcl1* in mice is associated with increased risk of development for B-cell lymphoma, T-cell lymphoma, and breast cancer.^25−27^ In addition to its role in tumorigenesis, Mcl-1 is also one of the most widely upregulated proteins responsible for development of resistance to existing drug therapies, including vincristine, taxol, gemcitabine, and cisplatin.^28−33^ Crucially, both tumorigenesis and drug resistance in cells with upregulated Mcl-1 can be reversed by RNAi, demonstrating the therapeutic potential of Mcl-1 inhibition.^2,29,34,35^ In addition to its direct role in regulating the apoptotic pathway, Mcl-1 is an even more attractive target as numerous studies have shown its synergistic potential when other cellular pathways, such as MAPK (Ras or MEK), mTOR, and ERK, are coinhibited.^36−42^

Inhibition of Mcl-1 with a small molecule inhibitor poses a significant challenge for drug discovery. Due to the need to closely regulate apoptosis, Mcl-1 binds very tightly to the BH3-only family members.^43^ All members of the Bcl-2 family possess a conserved BH3 domain, which consists of an amphipathic α-helix containing four key hydrophobic residues (L210, L213, V216, and V220 in Mcl-1), which interact with four corresponding hydrophobic pockets (P1–P4) located on the anti-apoptotic Bcl-2 family proteins.^8,43−47^ Given this large protein–protein interface that defines the binding interaction between BH3-only and anti-apoptotic family members, it is often necessary for inhibitors to exhibit extremely tight binding affinities (subnanomolar) before demonstrating activity in cellular experiments.^48^

**Figure 1:**
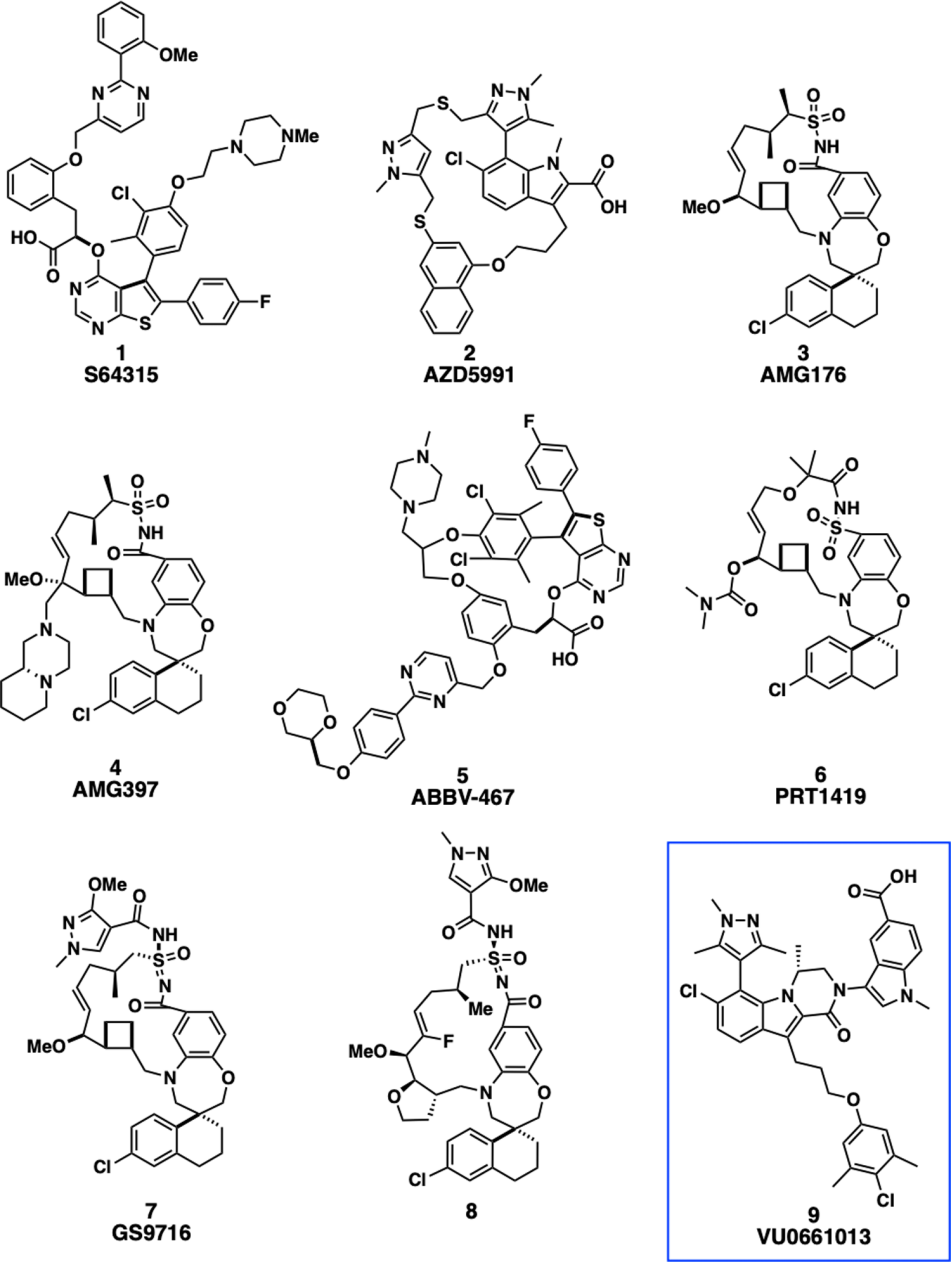
Recently Reported Clinical and Preclinical Mcl-1 Inhibitors.

Despite the challenges associated with inhibition of Mcl-1, potent small molecule Mcl-1 inhibitors (S64315 (**1**),^49,50^ AZD5991 (**2**),^51,52^ AMG176 (**3**),^53−57^ AMG397 (**4**),^58,59^ ABBV-467 (**5**),^60−64^ PRT1419 (**6**),^65,66^ GS9716 (**7**)^67−69^) have entered clinical trials within the last six years and have targeted not only hematological malignancies but also solid tumors, including breast, non-small cell lung cancer (NSCLC), small cell lung cancer (SCLC), cervical, and esophageal cancer (Figure 1). In addition to the efficacy of Mcl-1 inhibitors as a single agent, combination studies with chemotherapy and targeted therapies are also in progress (venetoclax, azacytidine, itraconazole). Additionally, inhibitor **8**,^70^ which is structurally similar to **7** and demonstrated antitumor efficacy in xenograft models and oral bioavailability, has been reported in the literature but has yet to enter the clinic. However, despite these recent advances in Mcl-1 inhibition, potential cytotoxicity of Mcl-1 inhibitors has presented a formidable barrier to further progression. While genetic deletion of Mcl-1 has been shown to be embryonically fatal in mice^71^ and siRNA knockout studies have demonstrated its importance in the survival of cardiomyocytes,^72,73^ hemopoietic stem cells,^74^ and lymphocytes,^75−77^ temporary pharmaceutical blockade has been well tolerated in mouse xenograft studies.^49,51,55^ In a humanized Mcl-1 mouse model, Mcl-1 inhibitor S63845, structurally related to compound **1**, was found to have a ∼3-fold lower maximum tolerated dose than in wild-type mice, but the changes observed in hemopoietic cells were transient and no organ damage was observed.^78,79^ In the clinic, however, compounds **1**, **4**, and **5** have reported dose limiting toxicities, including increases in patient troponin I levels, a possible indicator of cardiac injury.^50,59,63^ Furthermore, inhibition of the transcription factor CDK9, which results in downregulation of Mcl-1 among other proteins, has been associated with cardiomyocyte toxicity.^80,81^ Thus, an FDA-approved Mcl-1 inhibitor will need to not only deliver antitumor efficacy, but also successfully manage the associated patient risk.

Guo et al. have shown that disruption of cardiomyocyte function by siRNA is time dependent, with no change in contractile function observed 3 days post transfection, but a progressive reduction in beat amplitude and increase in beat rate on days 14 and 33.^82^ Compound **3** possesses a pharmacokinetic (PK) profile, a *T* = 14 h and *V*_ss_ = 0.6 L/kg,^55^ which would result in prolonged inhibition of Mcl-1, and potentially increase the risk of cardiomyocyte and other off-target toxicity. Additionally, Rasmussen et al. have demonstrated that structurally diverse small molecule Mcl-1 inhibitors impact cardiomyocyte mitochondrial networks and contractility to varying extents.^83^ In light of these observations, we have also pursued a structurally novel class of Mcl-1 inhibitors, possessing a *C*_max_-driven PK profile, with the goal of advancing these molecules to the clinic.^84−87^ We have previously reported a series of Mcl-1 inhibitors bearing a tricyclic (*R*)-methyl-dihydropyrazinoindolone core, as exemplified by compound **9**, which were also potent inhibitors of Mcl-1 (Figure 1).^88−90^ In this report, we describe our efforts to optimize the series to identify a candidate with a profile suitable for clinical development.

Our previously reported tricyclic dihydropyrazinoindolone series of Mcl-1 inhibitors, such as compound **9**, demonstrated excellent affinity for the Mcl-1 protein (*K*_*i*_ < 200 pM in time-resolved fluorescence resonance energy transfer (TR-FRET) binding assay) and highly selective antiproliferative activities in the Mcl-1-sensitive multiple myeloma cell line NCI-H929 (**9**, GI_50_ = 120 nM).^88^ The series also induced caspase activation in cancer cells in good correlation with Mcl-1 binding affinity to prove the inhibitory mechanism of action. Compound **9** was further evaluated by assessing its activity in *in vivo* cancer models where it demonstrated 60% tumor growth inhibition in the AMO-1 subcutaneous (SC) xenograft model by IP dosing (100 mg/kg QDx14). In an MV-4-11 disseminated leukemia model, treatment with compound **9** resulted in a dose-dependent reduction in tumor burden and nearly eliminated MV-4-11 cells (IP, 75 mg/kg QDx21) in the blood, bone marrow, and spleen.^90^ Finally, **9** also exhibited synergistic tumor growth inhibition in combination with a standard of care chemotherapeutic agent doxorubicin in HCC-1187 and BT-20 triple-negative breast cancer xenograft models.^88^ While these results obtained with compound **9** established an important proof of concept of *in vivo* efficacy for our series, we sought to further optimize this series to identify a compound with a profile suitable for clinical advancement. Key criteria to improve our Mcl-1 inhibitors include optimization of cellular potency, pharmaceutical, and PK properties to achieve robust *in vivo* efficacy using a clinically relevant dosing schedule.

## Results and Discussion

In our previous work, we systematically optimized the tricyclic indole-lactam core unit to identify the (*R*)-methyl-dihydropyrazinoindolone core unit as being optimal.^88^ Additionally, the *N*-aryl substituent on the amide nitrogen was screened, with the 5-carboxy-1*H*-indol-3-yl substitution of **9** being identified. In the current work, we first explored varying the point of attachment between the indole substituent and the amide nitrogen. To assess the impact of new modifications, compounds were profiled in a TR-FRET binding assay and a cellular growth inhibition assay which used Mcl-1-sensitive NCI-H929 cell line to measure cellular efficacy and the Mcl-1 insensitive cell line K562 to evaluate off-target liabilities.

We screened a number of indole substituents, varying both the point of attachment to the amide nitrogen and the location of the carboxylate moiety on the indole, and identified compound **10** (Table 1), where the (*R*)-methyl-dihydropyrazinoindolone nitrogen is attached to the indole 2-carboxylic acid at the 7-position. Compound **10** exhibits the same GI_50_ (187 nM) in NCI-H929 cells as **9** and maintains a comparable selectivity profile in the K562 cell line. However, the 2-carboxy-1*H*-indol-7-yl motif allows for functionalization of the indole 4- and 5-positions, which were further examined. Introduction of a methyl group at the 5-position (**11**) or a 5-methoxy substituent (**12**) results in a modest increase or decrease in potency, respectively, while introduction of a 4-methoxy group (**13**) leads to a ∼2-fold improvement in NCI-H929 GI_50_ relative to **10**. While a combination of both an alkyl and an ether substituent at the 4- and 5-positions (**15**–**17**) did not show an increase in cellular potency, the 4,5-dimethoxy compound **18** showed a significant improvement, with an NCI-H929 GI_50_ of 37 nM, a ∼5-fold increase relative to compound **10**. All of the compounds explored within this series maintain tight binding to the Mcl-1 protein as assessed in the TR-FRET assay, with binding affinities ranging between 90 to 280 pM. Furthermore, all of the compounds within this new 2-carboxy-1*H*-indol-7-yl series maintain a >1000-fold difference in the GI_50_ between the Mcl-1 sensitive NCI-H929 cell line and the insensitive K562 cell line.

**Table 1:**
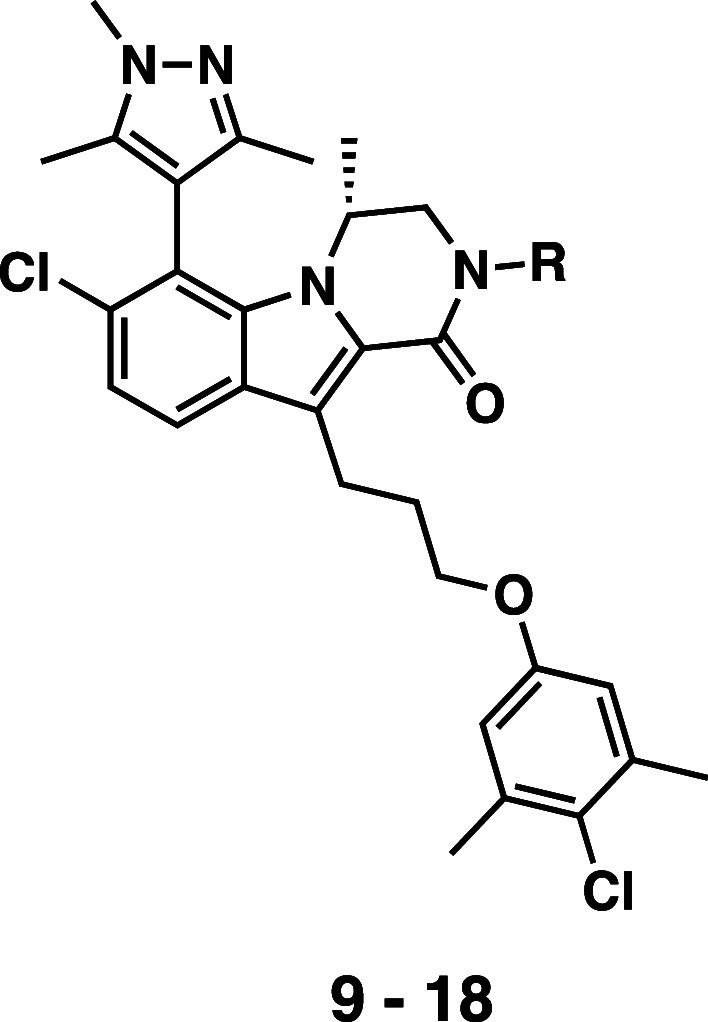

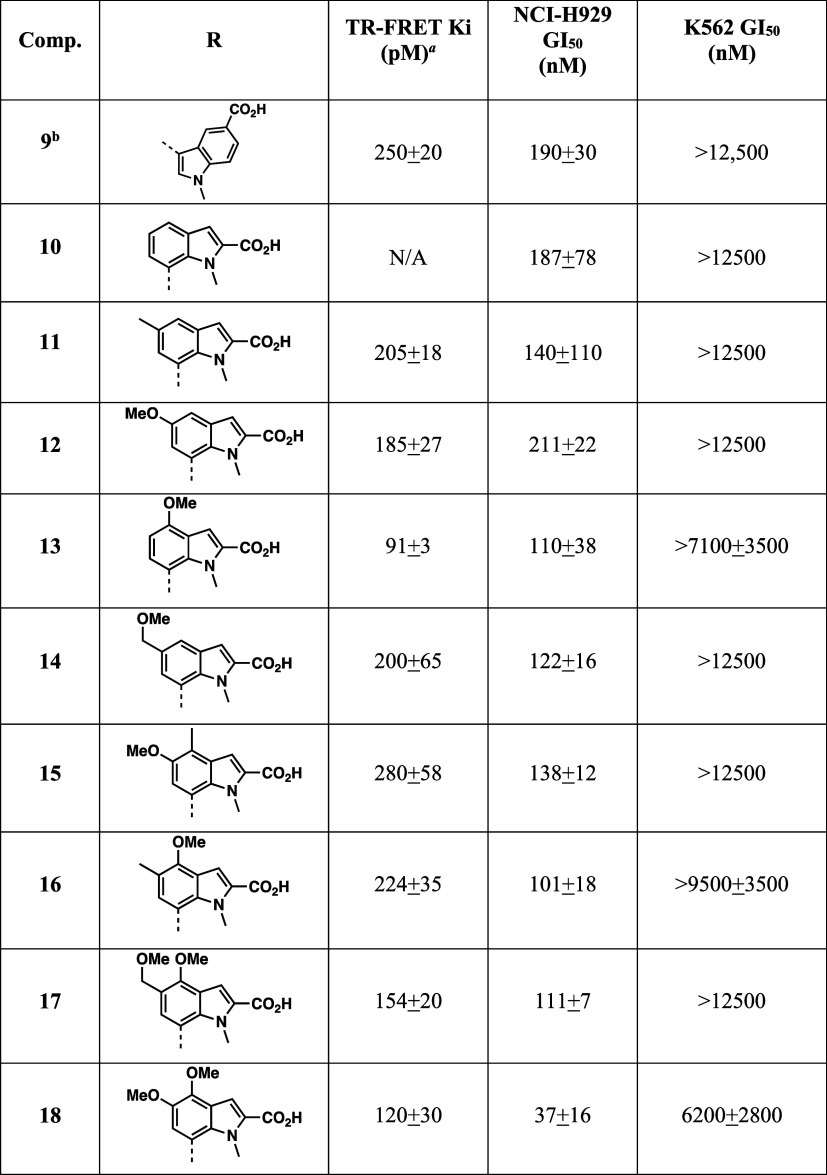
Binding Affinity and Cellular GI_50_ of Trimethyl Pyrazole Mcl-1 Inhibitors

a Mcl-1 *K*_*i*_ in the presence of 1% fetal bovine serum.

b Re-evaluated in proliferation assays with compounds **10**–**18**.

**Figure 2:**
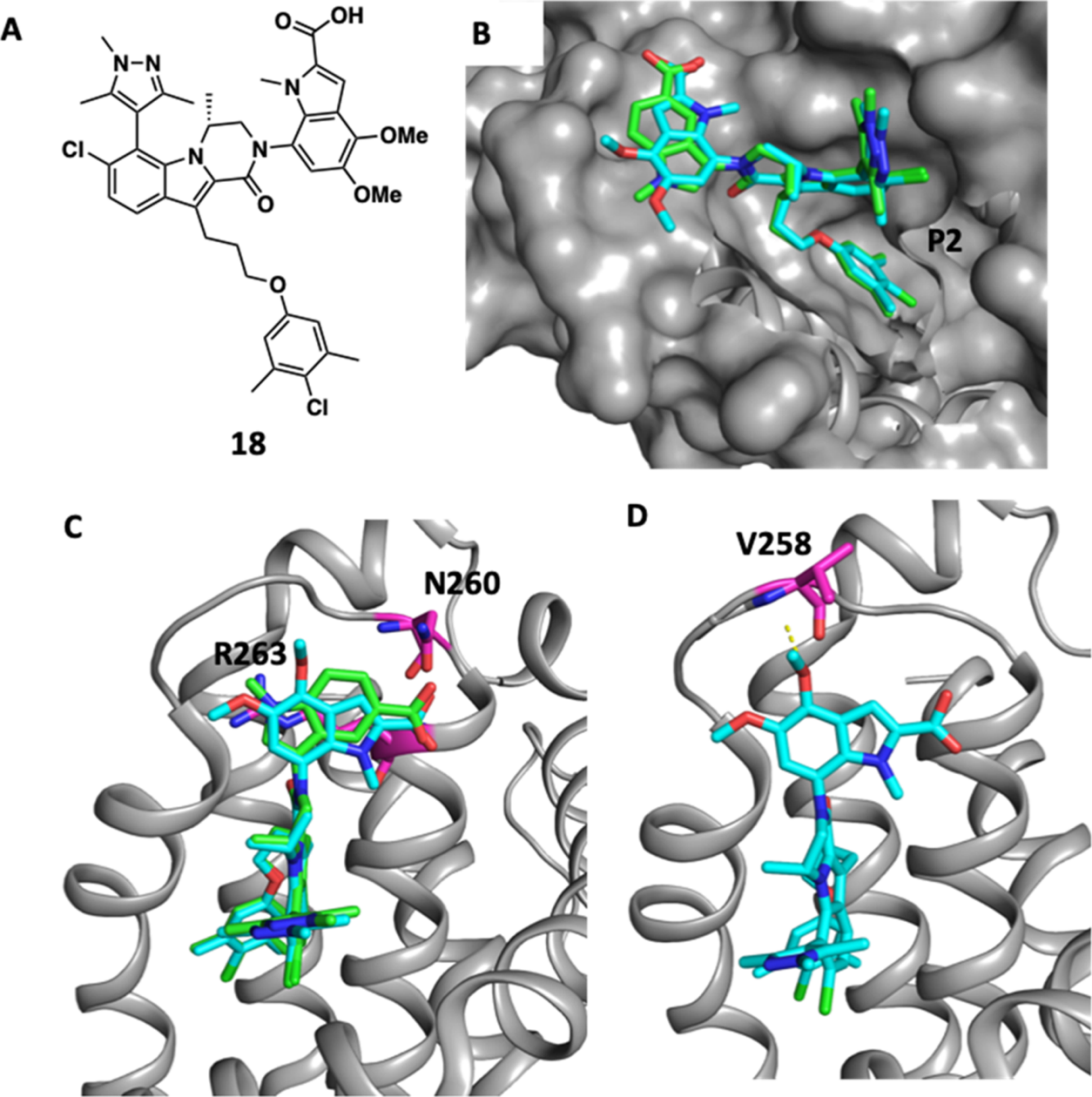
Comparison of X-ray Co-Crystal Structures of Mcl-1 Protein with **9** and **18**. **A**. Structure of compound **18**. **B**. Overlay of **9** (green) and **18** (cyan) bound to Mcl-1 protein, 3,5-dimethyl-4-chlorophenol moiety occupies induced P2 pocket of Mcl-1 (note: some authors refer to the induced pocket as P1 rather than P2). **C**. Overlay of **9** (green) and **18** (cyan) with Mcl-1 protein, π-stacking interaction with R263 (magenta) and hydrogen bonding interaction with N260 (magenta). **D**. Compound **18** bound to Mcl-1, polar interaction between 4-OMe and V258 (magenta).

To understand the binding interactions between the Mcl-1 protein and the 2-carboxy-1*H*-indol-7-yl series, we obtained the X-ray co-crystal structure of compound **18** bound to Mcl-1. The binding mode was then compared to the previously reported co-crystal structure of compound **9** (Figure 2).^85^ The overall binding pose is closely preserved in both compounds. In both crystal structures the atom positions of both the (*R*)-methyl-dihydropyrazinoindolone core and the 3,5-dimethyl-4-chlorophenoxy propyl moiety are nearly identical (Figure 2A). The 3,5-dimethyl-4-chlorophenol sits deep in the Mcl-1 P2 pocket where it forms an edge-to-face π-stacking interaction with Phe270 (Figure 2B). Despite the different attachment position of the C–N amide-indole bond, the indole moiety in both **9** and **18** forms a cation-π interaction with Arg263 (Figure 2C). This key interaction partially accounts for the observation whereby introduction of electron donating groups on the indole headpiece improves the binding affinity of our inhibitors. Additionally, both **9** and **18** present the carboxylic acid moiety in a similar position to the Mcl-1 peptide, where it forms a hydrogen bonding interaction with Asn260 (Figure 2C). Finally, we observe a conformational change in the Mcl-1 protein loop region to accommodate the bulkier substituted indole moiety. This occurs in the region where the peptide backbone sits closer to the small molecule, positioning the 4-methoxy group of **18** within hydrogen bonding distance of Val258 (Figure 2D). This proximity of the indole 4-position substituent to the loop region presents a sterically constrained environment, limiting the range of substituents that would likely be tolerated at this position. This prediction was born out in observation, as only small substituents (Me, Et, OMe, OEt) at the indole 4-position maintained good binding affinity and cellular potency. In contrast, the ether substituent at the indole 5-position is directed parallel to the shelf region of the protein, which offers significant space and an ideal vector for further exploration.

**Figure 3:**
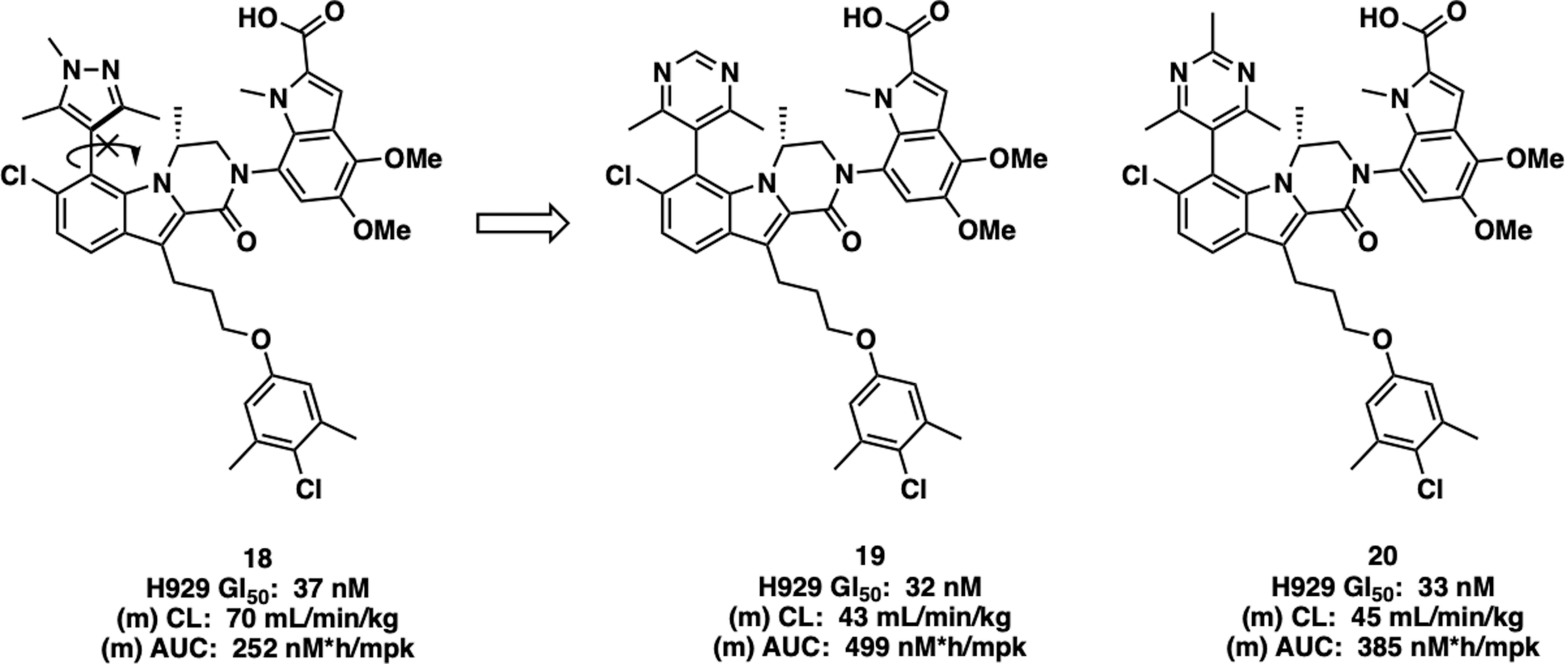
Comparison of Cellular Activity and PK^a^ of 1,3,5-Trimethylpyrazole (**18**), 4,6-Dimethylpyrimidine (**19**), and 2,4,6-Trimethylpyrimidine (**20**) Compounds. ^a^Mouse IV PK dosed at 25 mg/kg. AUC is dose normalized.

Prior to examining modifications of the 5-position, we directed our attention to the 1,3,5-trimethylpyrazole substituent. As previously reported, introduction of the 1,3,5-trimethylpyrazole substituent on the core indole generates an ortho–ortho′-substituted biaryl C–C bond with hindered rotation and no selectivity over the resultant axial stereocenter.^85^ Upon introduction of the (*R*)-methyl group of the piperazinone ring, the racemic atropisomers are rendered diastereomeric. The resultant diastereomers are separable by high-performance liquid chromatography (HPLC), thermally stable (no interconversion observed at 120 °C for 72 h), and can be used to access either atropisomer of the final compounds. We previously found that both (*M*) and (*P*) isomers had similar binding affinity to Mcl-1 and activity in the NCI-H929 growth inhibition assay, which was expected as the pyrazole *N*-methyl group is positioned toward the solvent and makes no significant interaction with the Mcl-1 protein.^85^ However, the (*M*)-diastereomer of **9** resulted in lower *in vivo* clearance, thus compound **18** was prepared as the single (*M*)-atropisomer for evaluation in mouse PK (Figure 3). While the diastereomeric atropisomers were separable by HPLC, the introduction of a separation step requiring careful HPLC purification and a loss of half of the material in the form of the undesired atropisomer posed a significant challenge to further progression of the series. To address this, we envisioned replacing the 1,3,5-trimethylpyrazole with a symmetrical heteroaryl group and thereby removing the site of axial chirality. As this moiety is oriented toward the solvent, this modification was not expected to significantly impact the binding of the inhibitor to Mcl-1. We thus replaced the 5-membered trimethylpyrazole substituent with both the symmetrical 4,6-dimethylpyrimidine (**19**) and 2,4,6-trimethylpyrimidine (**20**) rings. As predicted, both **19** and **20** show similar binding affinity to Mcl-1 and inhibition of NCI-H929 cell growth to compound **18**. Preserving a heterocyclic substituent at this position was necessary to maintain potency, as phenyl analogs resulted in a loss of potency (not shown).

The 4,6-dimethylpyrimidine **19** and 2,4,6-trimethylpyrimidine **20** were next evaluated in mouse IV PK to benchmark them against the 1,3,5-trimethylpyrazole series. Gratifyingly, both **19** and **20** showed a 2-fold improvement in the mouse IV clearance from (*M*)-**18**. Compound (*M*)-**18** possesses a clearance of 70 mL/min/kg and a dose-normalized AUC of 252 nM*h/mpk when dosed at 25 mg/kg in mice. Pyrimidines **19** and **20** showed a clearance of 43 and 45 mL/min/kg, respectively, with a corresponding increase in the dose-normalized AUC. Having identified two new series (4,6-dimethylpyrimidine and 2,4,6-trimethylpyrimidine) that did not face the same synthetic and development hurdles as the trimethylpyrazole series and possessing an improved PK profile, we sought to optimize these series by varying the ether substituents to further improve the physicochemical and PK disposition of each series.

We synthesized over 150 compounds bearing the 4,6-dimethyl or 2,4,6-trimethylpyrimidine moiety varying both the 4- and 5-position ethers on the indole ring. Introduction of aryl or larger aliphatic ethers at the 5-position resulted in reduced cellular efficacy and aqueous solubility. Zwitterionic nitrogen-containing ethers at the 5-position were well tolerated in terms of potency, but the mouse IV clearance of these compounds was inferior to the oxygen-bearing substituents. The most promising substituents with regards to maintaining efficacy in the NCI-H929 growth inhibition, aqueous solubility, and mouse IV clearance were cyclic oxygen-containing substituents at the 5-position and methyl or ethyl ethers at the 4-position. The most promising of these analogs are summarized in Table 2.

**Table 2:**
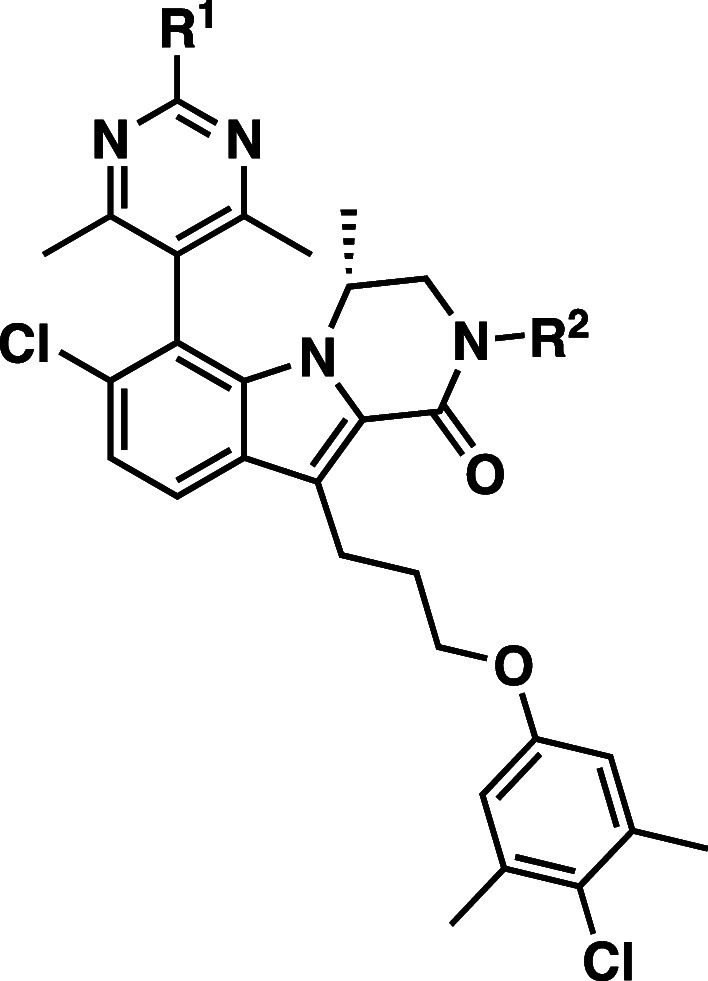

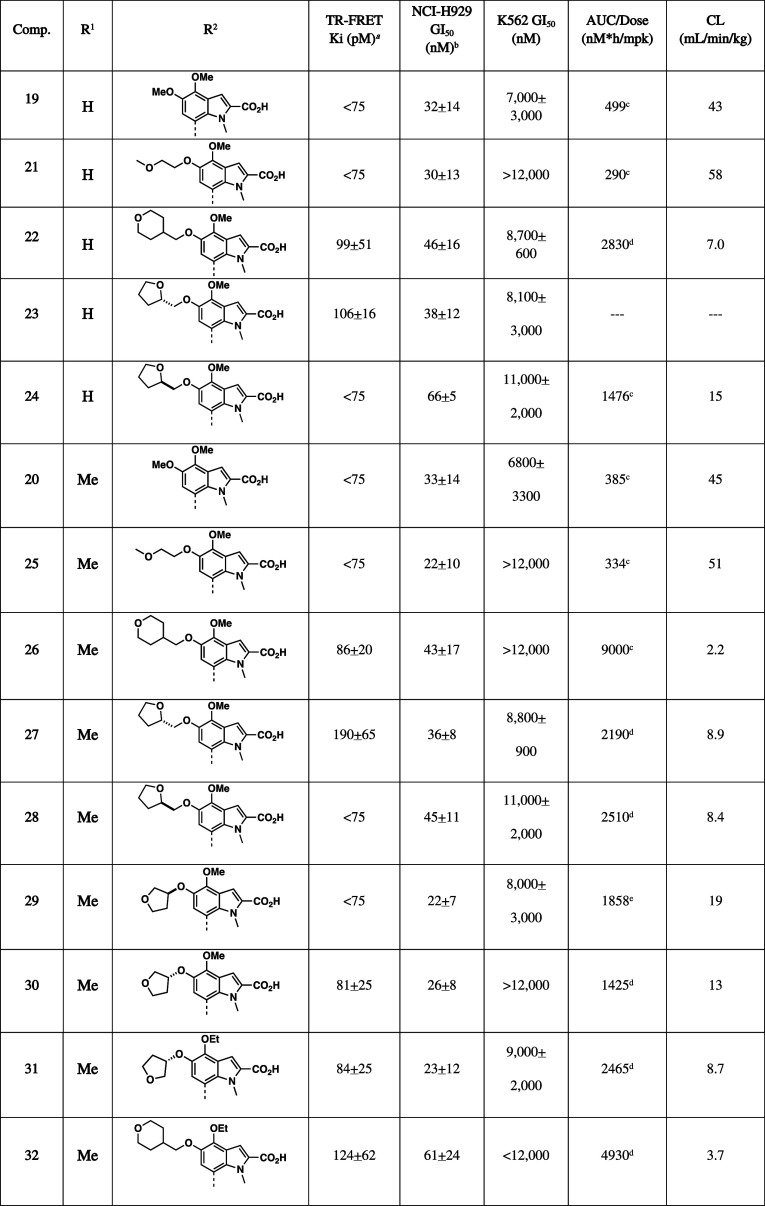
TR-FRET Binding Affinity, Cellular GI_50_, and Mouse IV PK of Pyrimidine Mcl-1 Inhibitors

a Mcl-1 *K*_*i*_ in the presence of 1% fetal bovine serum. All data points are an average of at least *n* = 2, run in triplicate.

b All data points are an average of at least *n* = 2, run in triplicate

c Dosed at 25 mg/kg.

d Dosed at 20 mg/kg.

e Dosed at 5 mg/kg

In general, these compounds maintain a comparable cellular potency in the NCI-H929 growth inhibition assay but offer significant improvement in the mouse IV clearance. All of the compounds exemplified in Table 2 show less than a 2-fold difference NCI-H929 GI_50_ relative to **19** or **20**, and maintain a good selectivity against K562. The similarity in binding affinity and cellular efficacy is not surprising given these modifications occupy the solvent-exposed shelf of the Mcl-1 binding site and do not make a significant interaction with the protein surface. However, these modifications do exert a significant impact on the IV clearance of the molecules. Incorporation of straight-chain ethers at the 5-position (**21**, **25**) results in an increase in the mouse IV clearance (58 and 51 mL/min/kg, respectively) from the 4,5-dimethoxy ether (**19**, **20**). However, all examples that incorporated a cyclic ether at the 5-position exhibited significantly lower clearance. The 2,4,6-trimethylpyrimidine series provides a ∼2-fold lower clearance than the 4,6-dimethylpyrimidines as can be observed from the matched pairs **21**/**25**, **22**/**26**, and **24**/**28**. For 5-position ethers containing a stereocenter, the difference at this site had little impact on the clearance of the molecules, with the (tetrahydrofuran-2-yl)methoxy ethers **27** and **28** having nearly identical mouse IV clearance, and the 3-tetrahydrofuryl ethers **29** and **30** showing only a modest difference (CL = 19 and 13 mL/min/kg, respectively). The best improvement in clearance was observed with the (4-tetrahydropyranyl)methyl substituent in both the dimethyl and trimethyl pyrimidine series (**22** and **26**, respectively). Compound **26** exhibited the lowest clearance for any compound tested (2.2 mL/min/kg), which corresponds to a 20-fold reduction in clearance from compound **20**, as well as commensurate increase in the exposure. In addition to its low clearance, **26** also exhibits a low volume of distribution (*V*_ss_ < 0.1 L/kg) resulting in a moderate half-life of *t*_1/2_ = 3.7 h, which limits its exposure temporally, decreasing potential risks of toxicity. Despite the improvement achieved in IV clearance from compounds **19** and **20**, all reported analogs showed low oral bioavailability in mice

Finally, modification of the 4-position ether was also evaluated. We hypothesized that substituting the methyl ether at this position may also improve the IV clearance; however, given the steric constraints of the nearby loop region of Mcl-1 binding site, 4-OEt was the largest ether that was well tolerated in terms of cellular potency. This modification did not yield a consistent trend for either potency or clearance. For matched pairs **29** and **31**, we saw virtually no change in the cellular potency; however, with **26** and **32** we observed a modest loss of potency. We observed an even more divergent trend when comparing the clearance data for the two matched pairs: exchanging the 4-OMe substituent for 4-OEt led a 2-fold decrease in the observed clearance for pair **29** and **31**; however, exhibited a 2-fold increase in clearance when comparing **26** and **32**.

In addition to the NCI-H929 growth inhibition assay and mouse PK studies, we also wanted to evaluate our compounds in dog PK, as dog would serve as a preclinical safety study species. We selected four compounds (**25**, **26**, **27**, and **29**) to evaluate in dog IV PK studies, that covered a range of mouse PK dispositions and ether structural variety (Table 3). Three of the compounds exhibited a higher clearance in dogs than was observed in the mouse studies. Compound **25**, which still showed the highest rate of clearance of any of the compounds tested in dog, possessed a similar clearance as a fraction of hepatic blood flow in both mouse and dog. Compounds **26**, **27**, and **29** all show a comparatively higher IV clearance in dog than in mouse. Of the compounds tested, **26** again showed the lowest clearance (5.2 mL/min/kg, 17% hepatic blood flow), which is 2- to 3-fold lower than the other compounds evaluated. Following the dog PK study, compound **26** was selected for further characterization prior to evaluation in *in vivo* efficacy studies.

**Table 3:** Dog IV PK Results of Pyrimidine Mcl-1 Inhibitors

comp.	AUC/dose (nM*h/mpk)	CL (mL/min/kg)	CL (% hep blood flow)
**25**^a^	1324	16	50
**26**^b^	3860	5.2	17
**27**^c^	2100	8.9	29
**29**^c^	1808	12	38

a Dosed at 0.25 mg/kg.

b Dosed at 0.5 mg/kg.

c Dosed at 0.9 mg/kg.

Compound **26** was found to maintain an excellent binding selectivity profile to Mcl-1 over other Bcl-2 family member proteins (Bcl-2 *K*_*i*_ = 1.8 μM, Bcl-XL *K*_*i*_ = 36 μM in FPA assay). The compound was found to have suitable aqueous solubility (15 mg/mL at pH 7.8) for IV formulation. Plasma protein binding of **26** was above the limit of quantification of our assay (>99.8% bound); however, relative differences in plasma binding between different species could be assessed by monitoring the shift in EC_50_ in a caspase induction assay. Using this paradigm, the plasma protein binding in mouse was found to be approximately equal to human, the plasma protein binding for dog and minipig were ∼2-fold lower than in human, and rat exhibited a 3-fold lower binding than human. Compound **26** was found to have good metabolic stability in hepatocytes (*h* = 12% Q_h_, *m* = 40% Q_h_, rat = 22% Q_h_, dog = 19% Q_h_, and minipig = 35% Q_h;_ incubated with 50% of human serum). It was also found to be a substrate of OATP1B1/ABCC2, and an excretion study in rat revealed that biliary clearance accounts for 60–75% of the total clearance. Compound **26** was found to be a moderate inhibitor of CYP3A4 (1.8 μM) and CYP2C8 (2.1 μM) and have moderate time dependent inhibition of CYP3A4 (67% function remaining following incubation). However, given the expected results of intermittent dosing, the high PPB resulting in very low unbound exposure, and no accumulation of compound in mice indicates a low risk as a DDI perpetrator.

**Figure 4:**
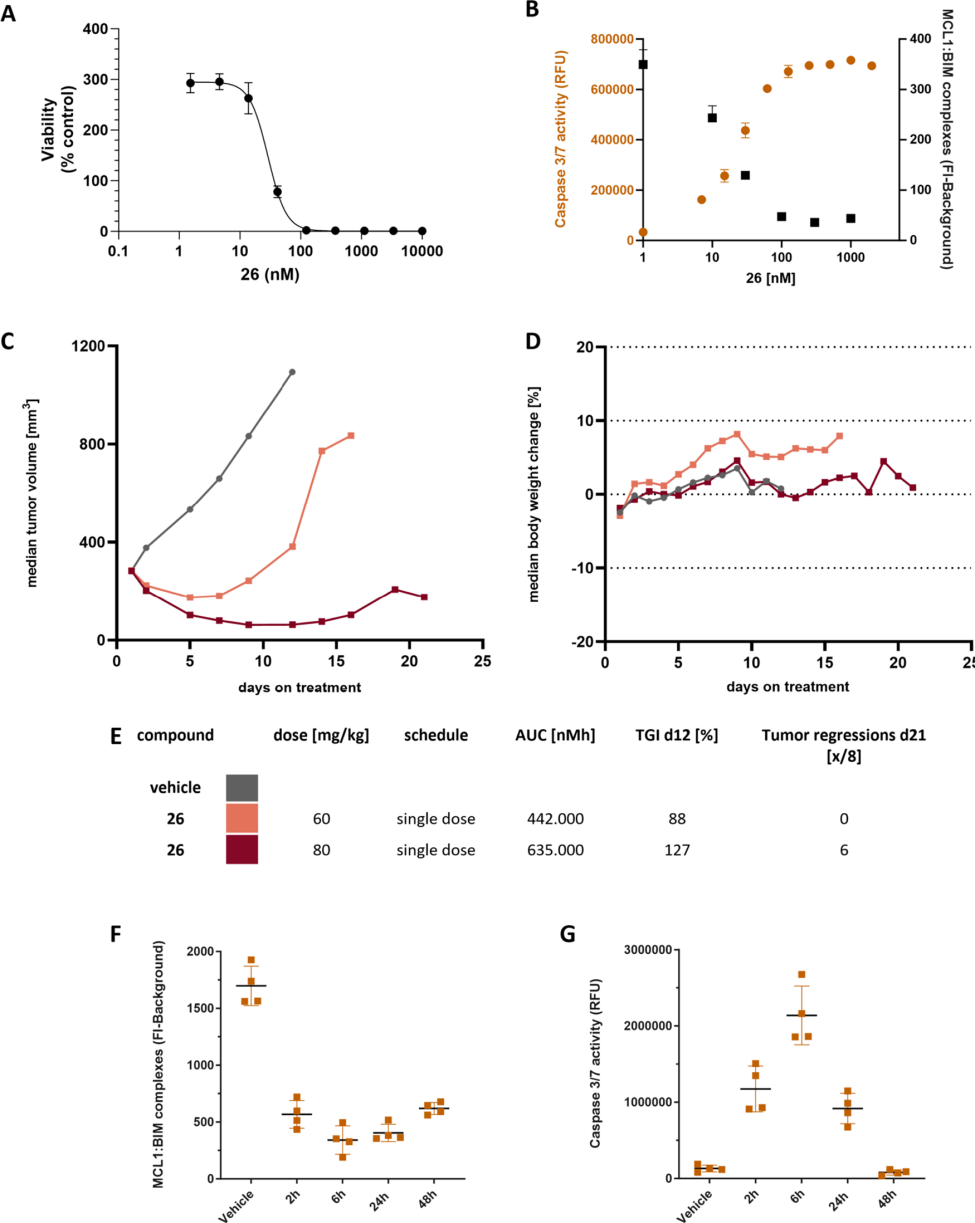
Efficacy study of **26** in the NCI-H929 cell line-derived MM xenograft model**A.** Cell viability following treatment with various doses of **26**; Viability is normalized to a control population measurement at the start of the experiment **B.***In vitro* dose-dependent effects of **26** in NCI-H929 cells at 4 h after treatment (data representative of at least three independent experiments with technical duplicates); **C.** Tumor volume of control (gray), **26** dosed at 60 mg/kg (orange), and **26** dosed at 80 mg/kg (dark red) administered as single IV dose. **D.** Body weights of control (gray), **26** dosed at 60 mg/kg (orange), and **26** dosed at 80 mg/kg (dark red) test groups. **E.** Tabulation of dose, AUC, tumor growth inhibition (TGI), and tumor regression. **F, G.** Treatment of NCI-H929-derived xenograft models with **26** showed significant induction of Caspase 3/7 activity and disruption of Mcl-1:BIM complexes with a maximum at 6 h after treatment.

Compound **26** was next evaluated in xenograft efficacy studies, the first being an NCI-H929 subcutaneous multiple myeloma xenograft model (Figure 4). On-target activity was first confirmed *in vitro*, where good correlation between the NCI-H929 growth inhibition of **26** and Mcl-1:Bim complex disruption and caspase 3/7 activation was observed. Compound **26** was dosed IV as a single dose at either 60 or 80 mg/kg. The two doses exhibited a dose-linear exposure profile and were both able to demonstrate initial tumor regression. The tumor regression at 60 mg/kg persisted until day 7 after dosing start; whereas the 80 mg/kg dose achieved a more durable effect with regression persisting until day 15. For both doses, no significant change was observed in the median weight of the mice. In line with the *in vitro* results, analysis of harvested tumor samples following treatment with **26** also showed a time-dependent disruption of Mcl-1:BIM complexes and an increase of caspase 3/7 activity *in vivo*, with maximum effect sizes being reached at 6 h post dosing.

**Figure 5:**
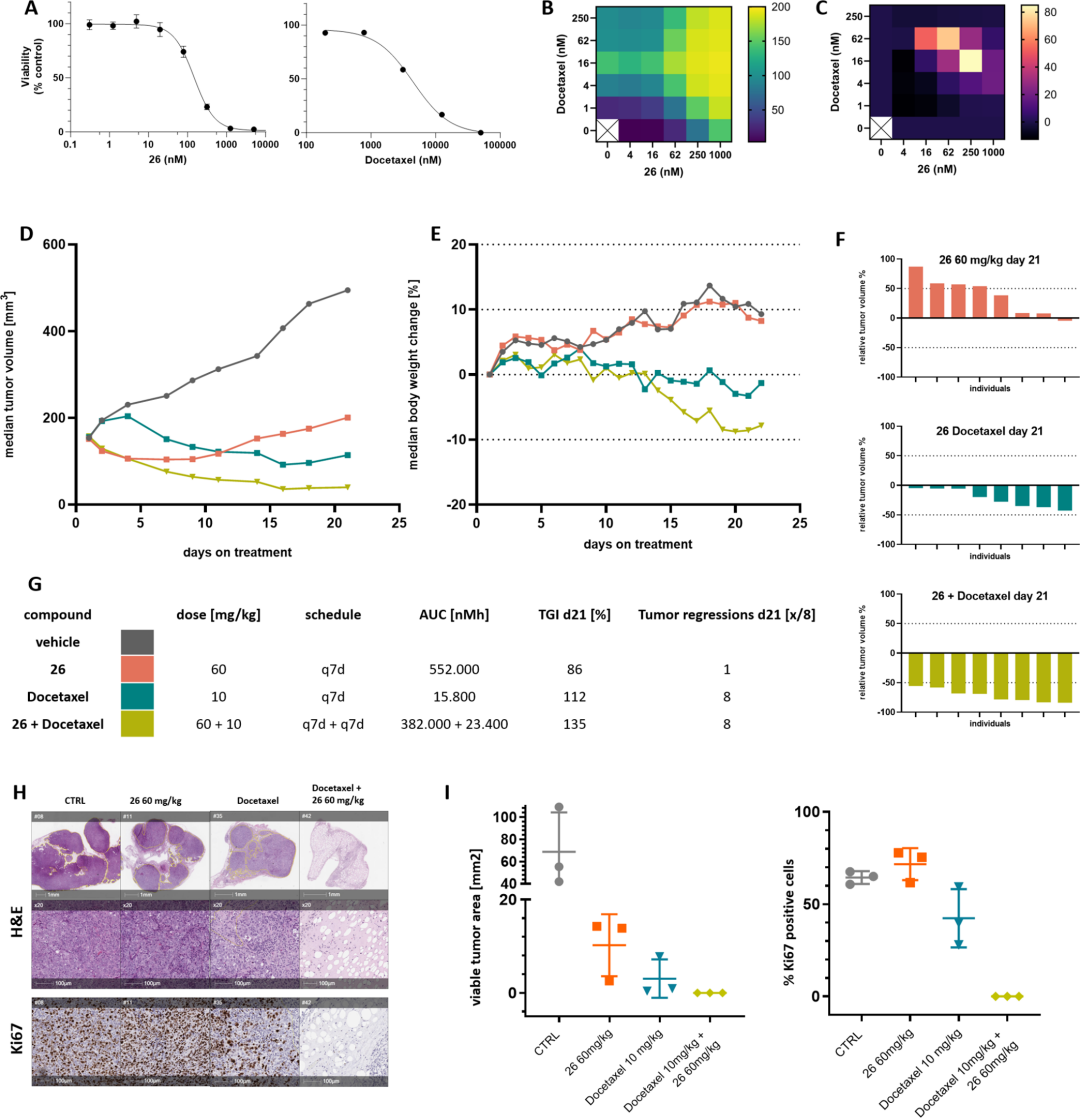
Efficacy study of **26** in the A427 cell line-derived NSCLC xenograft model.**A.** Cell viability measurement following treatment with **26** (left) or docetaxel (right) at indicated doses. Viability is normalized to control population measurement at the end of the treatment duration. **B.** Heatmap of cell growth inhibition. **C.** Synergy between the two treatments based on BLISS Gap score. **D.** Tumor volume of control (gray), **26** dosed at 60 mg/kg q7d (orange), docetaxel dosed at 10 mg/kg q7d (teal), and **26** dosed at 60 mg/kg q7d + docetaxel dosed at 10 mg/kg q7d (beige). **E.** Average body weight for each dosing cohort: control (gray), **26** dosed at 60 mg/kg q7d (orange), docetaxel dosed at 10 mg/kg q7d (teal), and **26** dosed at 60 mg/kg q7d + docetaxel dosed at 10 mg/kg q7d (beige). **F.** Comparison of relative tumor volumes for each dosing cohort. **G.** Tabulation of dose, AUC, % TGI, and % tumor regression. **H.** Representative images of hematoxylin and eosin (H&E) and Ki67 stained tumor sections for each dosing cohort. **I.** Summary of tumor viable area in mm^2^ and percentage of Ki67 positive cells for each tumor and treatment group.

We next evaluated compound **26** in an Mcl-1 sensitive non-small cell lung cancer cell line A427 both as a single agent and in combination with a standard of care chemotherapeutic agent, docetaxel (Figure 5). Compound **26** showed potency against A427 in a growth inhibition assay with a GI_50_ of 90 nM. As a single agent, docetaxel exhibited a GI_50_ of 4.4 μM in A427 cells. *In vitro* treatment of A427 cells with a combination of compound **26** and docetaxel resulted in enhanced growth inhibition and synergy based on BLISS gap analysis (Figure 5B). When compound **26** was dosed IV as a single agent at 60 mg/kg q7d in a subcutaneous xenograft model, tumor regression was observed with outgrowth occurring around day 7 after treatment start. On study day 21, best tumor control was in a single animal receiving **26** at 60 mg/kg exhibiting tumor stasis. A 10 mg/kg q7d dose of docetaxel alone resulted in tumor regression, with all study animals still exhibiting regressions at day 21; however, tumor outgrowth began around day 15 after treatment start. The combination of **26** and docetaxel (60 mg/kg + 10 mg/kg, q7d) resulted in a deepened response relative to either single agent treatment, with no sign of outgrowth after 21 days. To further characterize the residual tumors after 3 weeks of treatment, tumors were collected, formalin-fixed and embedded into paraffin blocks. Sections were then stained with hematoxylin and eosin or immunohistochemistry. While all treatment schedules resulted in a significant reduction in viable tumor area, the combination treatment arm showed no viable tumor cells in H&E and Ki67 staining, indicative of a full pathologic response. This underscores the therapeutic potential of combining pro-apoptotic therapy with the inhibition of anti-apoptotic proteins, such as Mcl-1. In addition to increasing the degree of tumor inhibition achieved relative to Mcl-1 monotherapy, the success of Mcl-1 inhibition in combination with other therapeutics could reduce the required dose of an Mcl-1 inhibitor, and thus mitigate the risk to healthy tissue.

**Figure 6:**
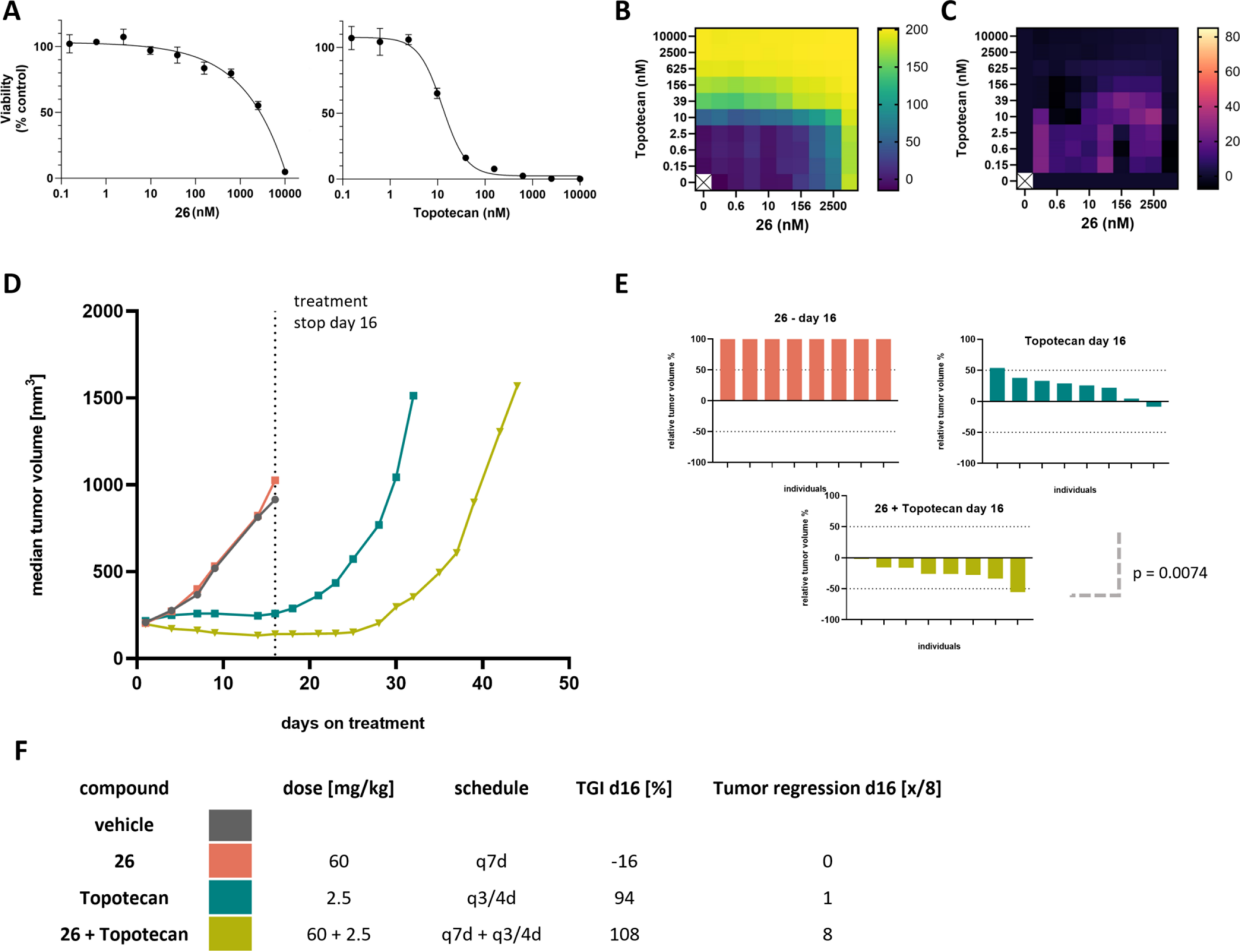
Efficacy Study of **26** in NCI-H1048 SCLC Xenograft Model. **A.** Cell viability measurement following treatment with **26** (left) or topotecan (right) at indicated doses. Viability is normalized a control population measurement at the end of the treatment duration. **B.** Heat map of cell growth inhibition and **C.** synergy between the two treatments based on BLISS Gap score. **D.** Tumor volume of control (gray), **26** dosed at 60 mg/kg q7d (orange), topotecan dosed at 2.5 mg/kg q3/4d (teal), and **26** dosed at 60 mg/kg q7d + topotecan dosed at 10 mg/kg q3/4d (beige). **E.** Comparison of relative tumor volumes for each dosing cohort. **F.** Tabulation of dose, AUC, % TGI, and % tumor regression.

Finally, compound **26** was evaluated in the SCLC model NCI-H1048 (Figure 6). *In vitro* evaluation revealed synergistic effects when combining topotecan (NCI-H1048 GI_50_ = 14 nM) with Mcl-1 inhibition, as evident by BLISS analysis, despite compound **26** showing weak single agent activity (GI_50_ = 2.3 μM) in NCI-H1048 cells. *In vivo*, compound **26** alone showed no impact on tumor growth whereas topotecan treatment had a substantial impact on tumor growth but was associated with fast outgrowth off treatment. Strikingly, the combination of these two agents lead to a deepened and prolonged response, with outgrowth occurring at a later time point, despite the lack of single agent activity for Mcl-1 inhibition. This result suggests that tumor sensitivity to Mcl-1 monotherapy was not required to see increased efficacy in Mcl-1 inhibition combination strategies and is in line with the concept of pro-apoptotic priming, where the pro-death activity of a combination therapy partner would generate a cellular state where apoptosis is kept in check by anti-apoptotic proteins, like Mcl-1.^91^ Importantly it is in such a scenario where inhibition of Mcl-1 may have the strongest therapeutic benefit.

**Scheme 1:**
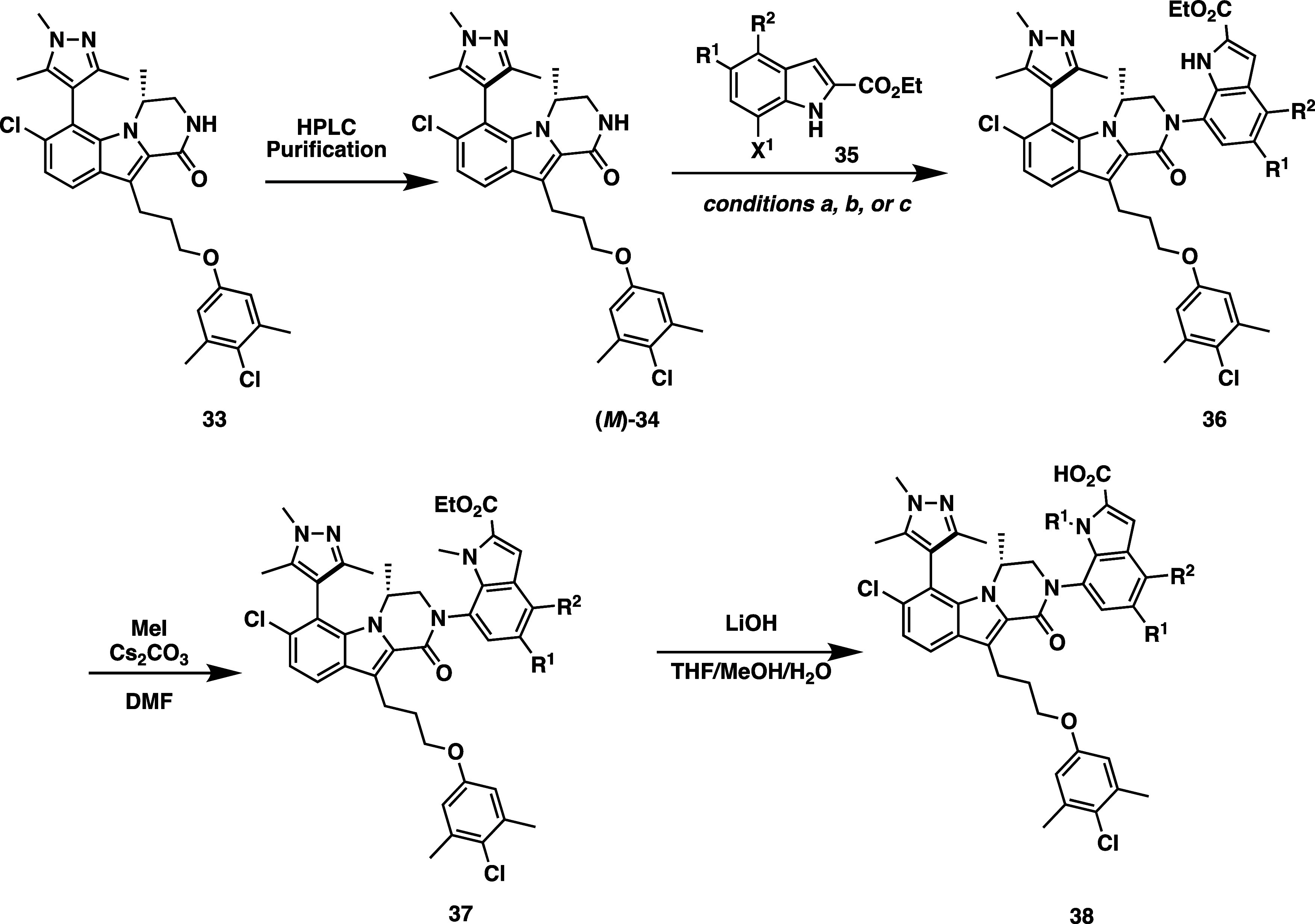
Synthetic Route for Pyrazole Series Inhibitors Conditions: (a) CuI, *N*^1^,*N*^2^-dimethylcyclohexane-1,2-diamine, K_3_PO_4_, toluene (b) Pd_2_(dba)_3_, Xantphos, Cs_2_CO_3_, dioxane (c) [Pd(cinnamyl)Cl]_2_, ^*t*^Bu-BrettPhos, Cs_2_CO_3_, toluene.

**Scheme 2:**
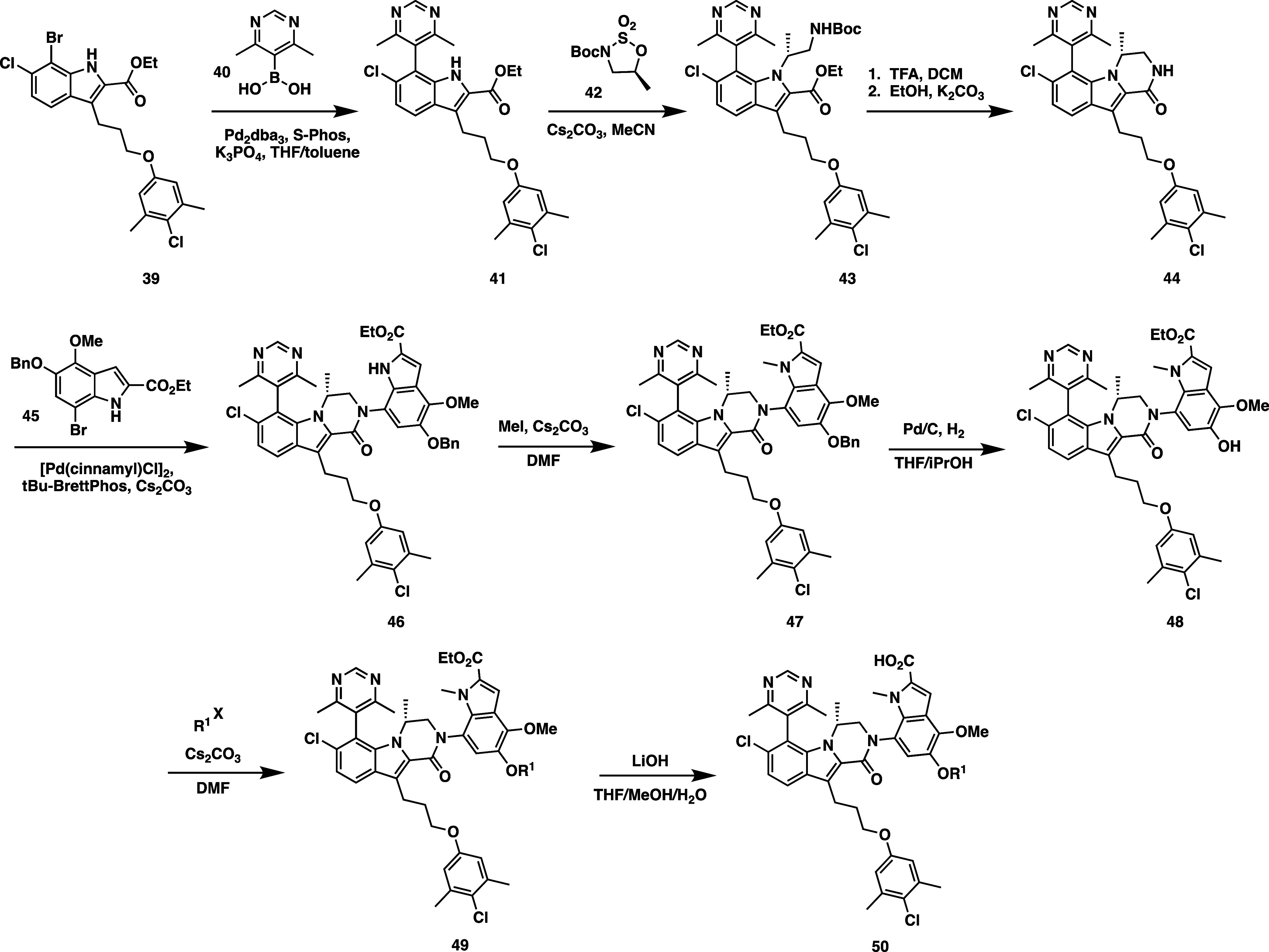
Synthetic Route for 4,6-Dimethyl Pyrimidine Series

**Scheme 3:**
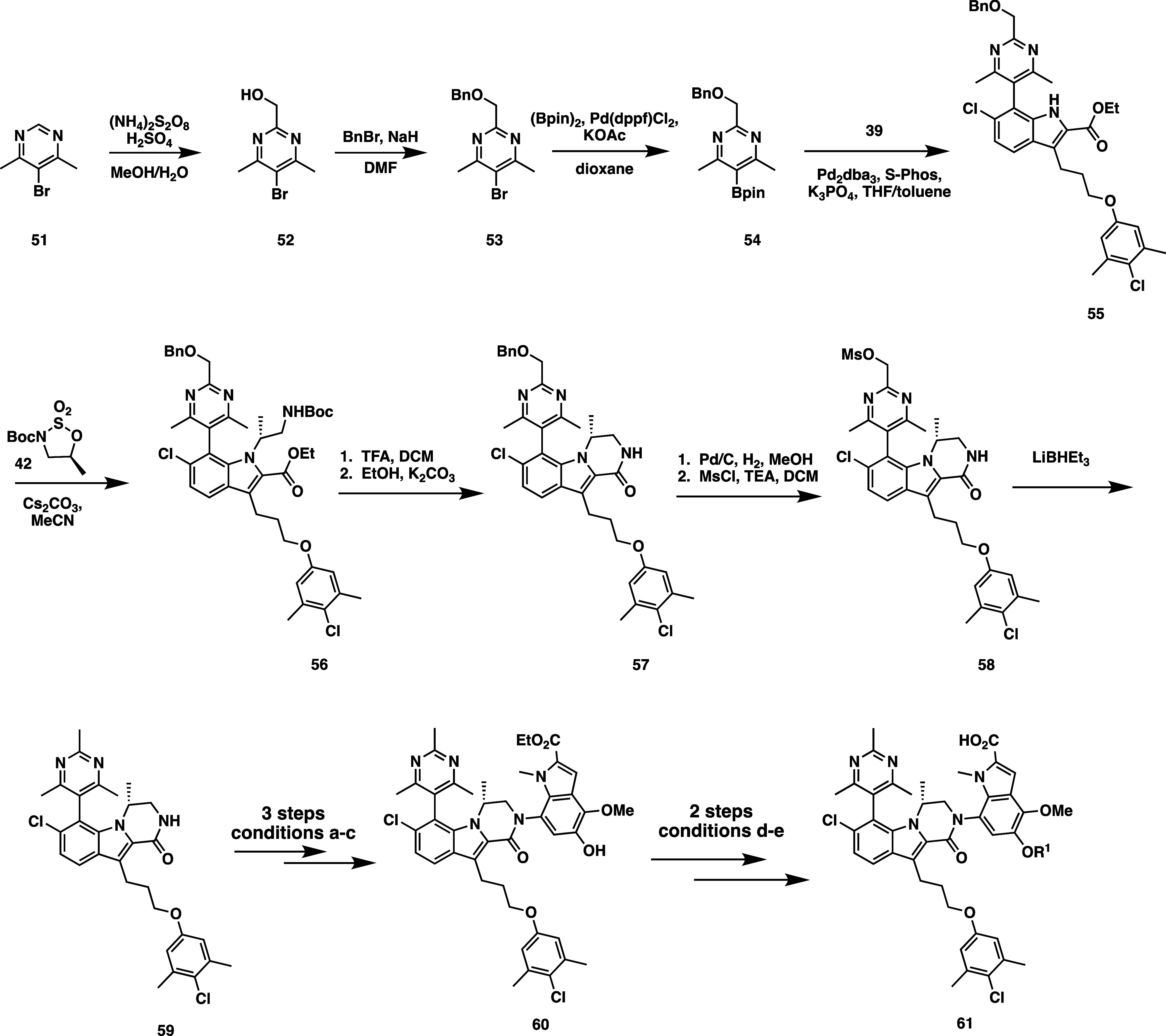
Synthetic Route for 2,4,6-Trimethyl Pyrimidine Series Conditions: (a) **45**, [Pd(cinnamyl)Cl]_2_, ^*t*^Bu-BrettPhos, Cs_2_CO_3_, toluene. (b) MeI, Cs_2_CO_3_, *N*,*N*-dimethylformamide (DMF). (c) Pd/C, Pd(OH)_2_/C, tetrahydrofuran (THF), isopropanol. (d) R^1^-X, Cs_2_CO_3_, DMF. (e) LiOH, THF**/**MeOH/H_2_O.

The Mcl-1 inhibitors described in this manuscript were prepared following the representative synthetic routes in Schemes 1–3. Synthesis of the 1,3,5-trimethylpyrazole series (Scheme 1) begins from compound **33**, whose synthesis as a mixture of axially chiral diastereomers has been previously reported.^85^ The (*R*)-methyl-dihydropyrazinoindolone core diastereomers can be separated by reverse phase HPLC to afford the conformationally stable diastereomer, (*M*)-**34**, shown. Compound (*M*)-**34** was then coupled to the 7-bromoindole or 7-iodoindole **35** under either Ullmann or Buchwald cross coupling conditions. Following the cross coupling, the nitrogen of indole intermediate **36** was alkylated with methyl iodide under basic conditions. Saponification with LiOH afforded the final 2-carboxylic acid analog, **38**.

Synthesis of the 4,6-dimethylpyrimidine series proceeds through a similar route illustrated in Scheme 2. Suzuki coupling of indole **39** with 4,6-dimethylpyrimidyl boronic acid (**40**) affords compound **41**, followed by indole *N*-alkylation with the sulfinate **42**. Boc deprotection with trifluoroacetic acid (TFA) and cyclization to the tricyclic piperazinone under basic conditions affords intermediate **44**. Compound **44** was then cross coupled using Buchwald conditions with indole **45** to afford **46**. Compound **46** could then be alkylated with MeI to afford **47**, which could in turn be hydrogenated to afford phenol **48**. Alkylation of the 5-OH position with the appropriate tosylate or alkyl halide furnished ester **49**, which could then be saponified using LiOH to furnish final analogs of structure **50**.

Synthesis of the trimethyl pyrimidine series (Scheme 3) began with conversion of 5-bromo-4,6-dimethylpyrimidine (**51**) to methyl alcohol **52** by treatment with ammonium persulfate in methanol. Alcohol **52** was protected with benzyl bromide to afford **53**, followed by boronylation under Miyaura conditions to give pinacol boronic ester **54**. Suzuki coupling to indole **39** afforded compound **55**. Sulfinate alkylation with **42**, Boc deprotection, and lactam cyclization afforded compound **57**. The resultant benzyloxy pyrimidine was deprotected with Pd/C under an atmosphere of H_2_, followed by mesylation of the resultant alcohol to afford compound **58**. Treatment of mesylate **58** with lithium triethylborohydride furnished trimethyl pyrimidine tricyclic core **59**, which could then be functionalized to final compounds (**62**) using the same synthetic sequence described for the 4,6-dimethylpyrimidine series.

## Conclusions

Inhibition of Mcl-1 has emerged as a promising strategy for the treatment of human cancers by restoring function in the intrinsic apoptotic pathway, which has often become dysregulated in cancer. Several new compounds have recently entered clinical trials; however, to date there is no approved therapy for Mcl-1 inhibition. In this manuscript we have described the design and synthesis of a novel Mcl-1 inhibitor, **26**, which shows increased potency and an improved pharmacokinetic profile, particularly a 20-fold improvement in mouse IV clearance relative to our previously disclosed inhibitors. Compound **26** was able to achieve tumor regression in both hematological and lung cancer xenograft models and demonstrated even more robust effects when dosed in combination with chemotherapeutic agents such as docetaxel or topotecan. Efforts are currently in progress to further improve the profile of the compounds within this series, including increasing potency and developing orally bioavailable inhibitors, in order to advance into clinical development.

## Experimental Section

### Chemistry

#### General

All NMR spectra were recorded at room temperature on a 400 MHz AMX Bruker spectrometer. ^1^H chemical shifts are reported in δ values in ppm downfield with the deuterated solvent as the internal standard. Data are reported as follows: chemical shift, multiplicity (s = singlet, d = doublet, t = triplet, q = quartet, br = broad, m = multiplet), integration, coupling constant (Hz). Low-resolution mass spectra were obtained on an Agilent 1200 series 6140 mass spectrometer with electrospray ionization. All samples were of ≥95% purity as analyzed by HPLC. Analytical HPLC was performed on an Agilent 1200 series with UV detection at 214 and 254 nm along with ELSD detection. LC/MS parameters were as follows: Method 1: Phenomenex-C18 Kinetex column, 50 × 2.1 mm, 2 min gradient, 5% (0.1% TFA/MeCN)/95% (0.1% TFA/H_2_O) to 95% (0.1% TFA/MeCN)/95% (0.1% TFA/H_2_O), Method 2: Phenomenex-C18 Kinetex column, 50 × 2.1 mm, 2 min gradient, 50% (0.1% TFA/MeCN)/50% (0.1% TFA/H_2_O) to 95% (0.1% TFA/MeCN)/5% (0.1% TFA/H_2_O). Preparative reverse phase purification was performed on a Gilson HPLC (Phenomenex-C18, 100 × 30 mm, 10 min gradient, 5 to 95% MeCN/H_2_O with 0.1% TFA). Normal phase purification was performed with Combi-flash Rf (plus-UV) Automated Flash Chromatography System. Solvents for reactions, extraction, and washing were ACS reagent grade, and solvents for chromatography were HPLC grade. All reagents were purchased from chemical suppliers and used without purification. Compounds **33**(87,88) and **39**(85) were synthesized as previously reported. LCMS traces for tested compounds are available in Supporting Information.

### Protein Expression and Purification for Assays and X-ray Structures

Protein preparation was described previously.^84^ Briefly, a previously reported construct was subcloned into an expression vector (pDEST-HisMBP) expressed in *Escherichia coli* BL21 CodonPlus (DE3) RIL (Stratagene) and purified through nickel-column and size-exclusion chromatography sequentially.

### Protein Crystallization, Data Collection, and Structure Refinement

Structural studies were performed as previously described.^84−88^ Briefly, Mcl-1 protein (15 mg/mL) was mixed with a 1.2× excess of ligand in solution (25–30% PEG 3350, 0.1 M Bis-TRIS pH 6.5, 0.2 M MgCl_2_) by hanging drop followed by flash freezing after cryo-protection using 10–20% glycol. Data were collected at Life Sciences Collaborative Access Team (LS-CAT) 21-ID-G beamline, Advanced Photon Source (APS), Argonne National Laboratory. Indexing, integration and scaling were performed with HKL2000 (HKL Research),^92^ phasing by molecular replacement with Phaser (CCP4)^93,94^ using the structure (PDB: 9BCG) as a model, refinement used Phenix.^95^ Structural statistics are given in the Supporting Information. Figures were prepared with PyMOL (Schrödinger, LLC: New York, 2010).

### TR-FRET Assay Conditions

A fluorescein isothiocyanate (FITC)-labeled BH3 peptide derived from Bak (FITC-Bak; FITC-AHx-GQVGRQLAIIGDDINR-NH2) was purchased from Genscript and used without further purification. TR-FRET measurements were made using 384-well, black, flat-bottom plates (Greiner Bio-One) containing 300 nM FITC labeled BAK peptide, 1 nM Mcl-1 6HIS fusion protein, 1 nM anti 6HIS-terbium (LanthaScreen Elite Tb-anti-HIS Antibody [Thermo Fisher]) and compound incubated in a buffer containing 4.5 mM monobasic potassium phosphate, 15.5 mM dibasic potassium phosphate, 1 mM EDTA, 50 mM NaCl, 1 mM DTT, 0.05% Pluronic F-68, pH 7.5. Mixtures containing vehicle without compound served as a negative control, while mixtures containing no protein served as a positive control. The mixtures were incubated for 3 h and signal (Delta F) was measured on the Biotek Cytation 3 equipped with a filter cube containing an Ex 340/30 nM Em 620/10 filter and an Ex 340/30 Em 520/10 filter. The ratio of 520/620 wavelengths was used to generate the TR-FRET signal. The TR-FRET signal was plotted versus compound concentration using XLFit (IDBS) curve fitting software to generate a four-parameter sigmoidal dose response (XLfit eq 205) to obtain an IC_50_. The IC_50_ was converted to a *K*_*i*_ using the following equation:
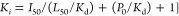
where [*I*]_50_ is the concentration of the free inhibitor at 50% inhibition (*I*_50_ = IC_50_ – *P*_0_ + PL_50_ [1+ (*K*_d_/*L*_50_)], *L*_50_ is the concentration of free ligand at 50% inhibition, *K*_d_ is the binding constant of the Bak BH3 peptide, *P*_0_ is the free protein concentration at 0% inhibition, PL_50_ is the protein–ligand complex concentration at 50% inhibition.^96^ Two or more repeats were obtained and average *K*_*i*_ values are reported.

### Cell Culture

NCI-H929, A427, NCI-H1048, and K562 cell lines were obtained from ATCC. NCI-H929 cells were cultured in RPMI1640 (ATCC-formulated, Catalog No. 30–2001) + 10% fetal calf serum (FCS, GIBCO BRL, Cat. No.: 26140) + 0,05 mM mercaptoethanol. NCI-H1975 and A-427 cells were cultured in RPMI1640 (ATCC-formulated, Catalog No. 30-2001) + 10% fetal calf serum (FCS, GIBCO BRL, Cat. No.: 26140). NCI-H1048 were cultured in custom medium prepared by PanBioTech containing DMEM/F12 + 5% fetal calf serum, 0.005 mg/mL insulin, 0.01 mg/mL transferrin, 30 nM sodium selenite, 10 nM hydrocortisone, 10 nM β-estradiol and 4.5 mM 2 mM l-glutamine. K562 cell lines were cultured in Iscove’s Modified Dulbecco Medium (Gibco-formulated, purchased from Thermo-Fisher, Cat. No.: 12440-053) + 10% fetal bovine serum (FBS, Sigma-Aldrich, F2442). All cell lines were cultured at 37 °C with 5% CO_2_ in a humidified incubator and have been regularly tested negatively for mycoplasm contamination.

### *In Vitro* Proliferation Assay

Cells were seeded at 750–1500 cells per well (depending on growth kinetics) in 384-well plates (Corning, Catalog No. 3707) or at 3000 cells per well in 96-well plates in the respective growth medium and allowed to settle overnight. Suspension cells were plated immediately before compound addition. Adherent cell lines were incubated overnight at 37 °C in a tissue culture incubator prior to compound addition. Compounds were added in triplicates using an HP D300e Digital Dispenser (Hewlett-Packard) or manually at the concentrations indicated and total volumes were normalized using dimethyl sulfoxide (DMSO) backfill, with final DMSO concentration of 0.5%. Control wells were treated with DMSO only. Following incubation for 72 or 96 h, a Cell Titer-Glo assay was conducted following the manufacturer recommendations (Promega, G9243) and signal was measured using a Victor X5 plate reader (PerkinElmer). %Viability was defined as relative luminescence units (RLU) of each well divided by the RLU of cells on day 0. Four parameter sigmoidal dose–response curves were generated and IC_50_ values were determined using XLFit (IDBS) software (equation 205) or in-house software. Drug combination experiments were performed in 96-well or 384- well format and drug synergy was determined using the BLISS model (PMID: 26171228).

### Complex Disruption and Caspase Activation Assay

Caspase-Glo 3/7 Assay (PROMEGA #G8090) was performed according to manufacturer’s instructions using 200 ng of sample lysate in a total volume of 100 μL per well. 100 μL of the GLO reagent were added, mixed, and incubated for 90 min at room temperature. The plate was measured on an EnSpireTM Plate reader (luminescence, 0.1s).

#### Complex Disruption Assay

The Milliplex Apoptosis Assay was carried out following manufacturer’s instructions with the MILLIPLEX Bcl-2 Family Apoptosis Panel 1 Magnetic Bead 6-plex Kit (#48-682MAG) using 20 μg of sample lysate as input. Incubation of sample and beads was performed as suggested at 4 °C overnight (16 h) in the dark, incubation with Biotinylated Detection Antibody was performed for 1 h at room temperature with shaking in the dark.

### Histopathology

After fixation, tumor samples were embedded in paraffin blocks. Two-μm thick sections of each FFPE-block were prepared on a microtome, placed on glass slides and dewaxed. H&E staining was performed using Papanicolaous Solution 1a Harris′ Hematoxylin solution (Merck) and Shandon Eosin Y aqueous (Thermo) on the Gemini stainer (Thermo). Ki67 staining was performed on the automated platform BOND RX (Leica) using a rabbit antibody against human Ki67 (clone D2H10 from CST) at 1:400 dilution with 20 min heat mediated antigen retrieval at pH 6, followed by DAB. Following the automated staining run, the slides were washed in a mild detergent, then thoroughly rinsed in distilled water, put in a 90% ethanol bath for 1 min, then moved to three baths of 100% ethanol for 1 min, then moved to two baths of xylene for 30 s, and finally coverslipped with mounting medium. Analysis was performed using HALO image analysis software 2.1 (Indica Laboratories). Quantification of Ki67 positivity was performed using multiplex IHC module with integrated AI based nucleus detection and a threshold set for positive nuclei was used. Viable tumor area was calculated from H&E staining using AI based classifier Densenet V2.

### *In Vivo* Xenograft Experiments

Female BomTac:NMRI-Foxn1nu mice and CB17/Icr-Prkdc(scid)/IcrCrl and were obtained from Taconic Denmark at an age of 6–8 weeks. After arrival of the local animal facility at Boehringer Ingelheim RCV GmbH & Co KG mice were allowed to adjust to housing conditions at least for 5 days before the start of the experiment. Mice were group-housed under pathogen-free and controlled environmental conditions and handled according to the institutional, governmental and European Union guidelines (Austrian Animal Protection Laws, GV-SOLAS and FELASA guidelines). Animal studies were approved by the internal ethics committee and the local governmental committee. To establish subcutaneous tumors mice were injected with 5 × 10^6^ A427cells in Matrigel (CB17/Icr-Prkdc(scid)/IcrCrl), 1 × 10^6^ NCI-H1048 cells in Matrigel (BomTac:NMRI-Foxn1nu and 5 × 10^6^ NCI-H929 cells in Matrigel (CB17/Icr-Prkdc(scid)/IcrCrl). Tumor diameters were measured with a caliper three times a week. The volume of each tumor [in mm^3^] was calculated according to the formula “tumor volume = length × diameter^2^ × π/6.” To monitor side effects of treatment, mice were inspected daily for abnormalities and body weight was determined three times per week. Animals were sacrificed when the tumors reached a size of 1500 mm^3^. Mice were dispatched randomly into treatment groups when the tumor size was 290 mm^3^ (NCI-H929), 153 mm^3^ (A-427) and 207 mm^3^ (NCI-H1048). Tumors were reported as regressing when the tumor volume at a given day was below the tumor volume at treatment start.

### Pharmacokinetic Analyses

For PK analysis, mice and dogs were administered intravenously with compound formulated either in 10% ethanol and 10% Cremophor EL for mice or in 50% ethanol and 50% PEG500 for dogs. Plasma samples were obtained at predefined time points and compound concentrations in plasma were measured by quantitative HPLC-MS/MS using an internal standard. Calibration and quality control samples were prepared using blank plasma from untreated animals. Samples were precipitated with acetonitrile and injected into a HPLC system (Agilent 1200). Separation was performed by gradients of 5 mmol/L ammonium acetate pH 5.0 and acetonitrile with 0.1% formic acid on a Luna C8 reversed-phase column with 2.5 μm particles (Phenomenex). The HPLC was interfaced by ESI operated in positive ionization mode to a triple quadrupole mass spectrometer (6500+ Triple Quad System, SCIEX) operated in multiple reaction monitoring mode. Chromatograms were analyzed with Analyst (SCIEX) and pharmacokinetic parameters were calculated by noncompartmental analysis using proprietary software.

### Chemistry General Procedures

#### General Procedure A: Ullmann Cross Coupling

In a reaction vessel, the (*R*)-methyl-dihydropyrazinoindolone core (e.g., **33**, **44**, or **59**) (1.0 equiv), 7-bromoindole or 7-iodoindole (1.5 equiv), CuI (0.5 equiv), (*trans*)-1,2-*N*,*N*′-dimethylaminocyclohexane (0.5 equiv), and K_2_CO_3_ (2.0 equiv) were massed. The reaction vessel was charged with toluene (0.4 M) and sparged with argon for 5 min. The vessel was then sealed and heated to 100 °C for 48 h. The reaction was cooled to room temperature and diluted with 1:1 EtOAc/H_2_O. The aqueous layer was separated and extracted with EtOAc (2×). The combined organic layers were washed with sat. NH_4_Cl, sat. NaHCO_3_, water, and brine. The organic layer was dried over MgSO_4_, filtered, and concentrated. The crude residue was purified by flash column chromatography eluting with either EtOAc/hexanes to afford the desired compound.

#### General Procedure B: Buchwald Cross Coupling Procedure 1

In a reaction vessel, the (*R*)-methyl-dihydropyrazinoindolone core (e.g., **33**, **44**, or **59**) (1.0 equiv), 7-bromoindole or 7-iodoindole (1.5 equiv), Pd_2_(dba)_3_ (0.1 equiv), Xantphos (0.2 equiv), and Cs_2_CO_3_ (2.5 equiv) were massed. The reaction vessel was charged with toluene (0.4 M) and sparged with argon for 5 min. The reaction was sealed and heated to 100 °C for 18 h. The reaction was cooled to room temperature and diluted with 1:1 EtOAc/H_2_O. The aqueous layer was separated and extracted with EtOAc (2×). The combined organic layers were washed with sat. NH_4_Cl, sat. NaHCO_3_, water, and brine. The organic layer was dried over MgSO_4_, filtered, and concentrated *in vacuo*. The crude residue was purified by flash column chromatography eluting with either EtOAc/hexanes to afford the desired compound.

#### General Procedure C: Buchwald Cross Coupling Procedure 2

In a reaction vessel, the (*R*)-methyl-dihydropyrazinoindolone core (e.g., **33**, **44**, or **59**) (1.0 equiv), 7-bromoindole or 7-iodoindole (1.2 equiv), [Pd(cinnamyl)Cl]_2_ (0.05 equiv), ^*t*^Bu-BrettPhos (0.1 equiv), and Cs_2_CO_3_ (4.0 equiv) were massed. The reaction vessel was charged with toluene (0.2 M) and sparged with argon for 5 min. The reaction was sealed and heated to 100 °C for 2–24 h. The reaction was cooled to room temperature and diluted with 1:1 EtOAc/H_2_O. The aqueous layer was separated and extracted with EtOAc (2×). The combined organic layers were washed with sat. NH_4_Cl, sat. NaHCO_3_, water, and brine. The organic layer was dried over MgSO_4_, filtered, and concentrated. The crude residue was purified by flash column chromatography eluting with either EtOAc/hexanes to afford the desired compound.

#### General Procedure D: Indole *N*-Methylation

In a reaction vessel, the *N*-H indole core (e.g., **36**, **46**) (1.0 equiv) was dissolved in DMF (0.2 M) followed by addition of Cs_2_CO_3_ (2.0 equiv). MeI was added and the reaction was heated to 60 °C for 3 h. The reaction was cooled to room temperature and diluted with 1:1 EtOAc/H_2_O. The aqueous layer was separated and extracted with EtOAc (2×). The combined organic layers were washed with sat. NH_4_Cl, sat. NaHCO_3_, water, and brine. The organic layer was dried over MgSO_4_, filtered, and concentrated. The crude residue was purified by flash column chromatography eluting with either EtOAc/hexanes to afford the desired compound.

#### General Procedure E: Hydrogenolysis of Benzyl Ether

In a reaction vessel, the benzylic ether (e.g., **47**) (1.0 equiv) was dissolved in THF/^*i*^PrOH (3:1) and the resultant mixture was sparged with argon for 5 min. Pd/C (10% wt, 0.1 equiv) and Pd(OH)_2_/C (20% wt, 0.1 equiv) were added to the reaction vessel, and the reaction was flushed with argon. The reaction mixture was allowed to stir under an atmosphere of H_2_ at 40 °C until complete by LCMS. The reaction mixture was filtered through a pad of Celite, rinsed with DCM, and concentrated. The crude residue was used without further purification.

#### General Procedure F: Alkylation of Indole 5-OH

In a reaction vessel, the 5-hydroxy indole core (e.g., **48**) (1.0 equiv) was dissolved in DMF (0.2 M). Cs_2_CO_3_ (3.0 equiv) was added, followed by addition of the appropriate alkylating agent (2.0 equiv). The reaction was heated to 90 °C and allowed to stir for 3 h, after which time the reaction was determined to be complete by LCMS. The reaction was cooled to room temperature and diluted with 1:1 EtOAc/H_2_O. The aqueous layer was separated and extracted with EtOAc (2×). The combined organic layers were washed with sat. NH_4_Cl, sat. NaHCO_3_, water, and brine. The organic layer was dried over MgSO_4_, filtered, and concentrated. The crude residue was purified by flash column chromatography eluting with either EtOAc/hexanes or MeOH/DCM to afford the desired compound.

#### General Procedure G: Saponification of Indole Ester

In a reaction vessel, the ester (e.g., **37**, **49**, **61**) was dissolved in THF/MeOH/H_2_O (5:1:1, 0.2 M). LiOH (10 equiv) was added, and the reaction was heated at 50 °C for 3–24 h until the LCMS shows complete conversion. The reaction was extracted with DCM, acidified with 1 M HCl, washed with H_2_O, washed with brine, dried over MgSO_4_, filtered, and concentrated. The crude residue was purified by reverse phase HPLC eluting with MeCN/H_2_O with 0.1% TFA additive. The resultant compound was concentrated, dissolved in DCM, washed with aq. NaHCO_3_, dried with MgSO_4_, filtered, and concentrated to afford the desired product.

### Syntheses of Key Intermediates

#### (*M*,*R*)-7-Chloro-10-(3-(4-chloro-3,5-dimethylphenoxy)propyl)-4-methyl-6-(1,3,5-trimethyl-1*H*-pyrazol-4-yl)-3,4-dihydropyrazino[1,2-*a*]indol-1(2*H*)-one ((*M*)-**34**)

The diastereomeric mixture of atropisomers **33** was separated by reverse phase HPLC (Phenomenex Gemini C18, H_2_O/CH_3_CN gradient from 50% to 95% CH_3_CN for 10 min, 0.1% TFA) to afford ∼40% of each atropisomer. Once separated, the atropisomers showed no interconversion after 72 h at 120 °C in DMSO. The absolute stereochemistry of (*M*)-**34** was confirmed by X-ray crystallography. (*M*)-**34**: LCMS Method 2: *R*_T_ = 1.281 min, MS (ESI): *m*/*z* 539.2 (M + H)^+^, ^1^H NMR (400 MHz, CDCl_3_) δ 7.63 (d, *J* = 8.8 Hz, 1H), 7.24 (d, *J* = 8.8 Hz, 1H), 6.63 (s, 2H), 5.73 (d, *J* = 5.2 Hz, 1H), 4.23–4.20 (m, 1H), 4.02–3.97 (m, 2H), 3.85 (s, 3H), 3.82 (dd, *J* = 21.2, 8.4 Hz, 1H), 3.44–3.28 (m, 2H), 3.19 (ddd, *J* = 12.8, 5.6, 1.6 Hz, 1H), 2.33 (s, 6H), 1.96 (s, 3H), 0.96 (d, *J* = 6.4 Hz, 3H). (*P*)-**34**: LCMS Method 2: *R*_T_ = 1.213 min, MS (ESI): *m*/*z* 539.2 (M + H)^+^, ^1^H NMR (400 MHz, CDCl_3_) δ 7.63 (d, *J* = 8.4 Hz, 1H), 7.24 (d, *J* = 4.4 Hz, 1H), 6.63 (s, 2H), 5.86 (d, *J* = 4.8 Hz, 1H), 4.13–4.10 (m, 1H), 4.00 (dt, *J* = 6.4, 2.0 Hz, 2H), 3.85 (s, 3H), 3.78 (dd, *J* = 12.4, 4.0 Hz, 1H), 3.44–3.28 (m, 2H), 3.20 (ddd, *J* = 12.4, 5.6, 1.6 Hz, 1H), 3.33 (s, 6H), 2.20–2.16 (m, 5H), 1.96 (3, 3H), 1.02 (d, *J* = 6.4 Hz, 3H).

#### (*R*)-7-Chloro-10-(3-(4-chloro-3,5-dimethylphenoxy)propyl)-6-(4,6-dimethylpyrimidin-5-yl)-4-methyl-3,4-dihydropyrazino[1,2-*a*]indol-1(2*H*)-one (**44**)

In a heavy-walled flask equipped with a stir bar compound **39** (8.2 g, 16.5 mmol), (4,6-dimethylpyrimidin-5-yl)boronic acid (**40**) (8.5 g, 35.6 mmol), 2-dicyclohexylphosphino-2′,6′-dimethoxybiphenyl (1.0 g, 2.5 mmol), Pd_2_(dba)_3_ (1.20 g, 1.3 mmol), and K_3_PO_4_ (17.4 g, 82 mmol) were massed. Toluene (80 mL) and THF (80 mL) were added, and the reaction was sparged with argon for 5 min. The flask was sealed and heated to 110 °C overnight. The reaction was poured into brine and extracted with EtOAc. The combined organic layers were dried over MgSO_4_, filtered, and concentrated. The crude residue was purified by flash column chromatography eluting with 0 to 100% EtOAc in hexanes to afford compound **41** (7.19 g, 83% yield). To a round-bottomed flask compound **41** (8.0 g, 15 mmol), *tert*-butyl (*S*)-5-methyl-1,2,3-oxathiazolidine-3-carboxylate 2,2-dioxide (**42**, 9.0 g, 38 mmol), and Cs_2_CO_3_ (9.9 g, 30.4 mmol). Anhydrous MeCN (100 mL) was added and the solution was stirred at 80 °C overnight. The reaction mixture was diluted with EtOAc, and washed with brine, dried over MgSO_4_, filtered, and then concentrated. The residue was purified by flash column chromatography eluting with 0 to 100% EtOAc in hexanes to afford **43** (8.0 g in 77% yield). Compound **43** was dissolved in DCM (80 mL) and cooled to 0 °C. TFA (20 mL) was added dropwise and the reaction mixture was allowed to stir at room temperature for 4 h. The solvent was removed *in vacuo*. Anhydrous EtOH (80 mL) was added followed by K_2_CO_3_ (6.8 g, 49 mmol). The reaction mixture was stirred at 50 °C for 2 h. The reaction mixture was concentrated to 1/3 volume and diluted with ethyl acetate (100 mL) and washed with brine (3 × 50 mL). The combined organic layers were dried over MgSO_4_, filtered, and concentrated. The crude residue was purified by flash column chromatography eluting with 0 to 5% MeOH in DCM to afford **44** (5.2 g, 84% yield). LCMS Method 2: *R*_T_ = 1.313 min, MS (ESI): *m*/*z* 537.2 (M + H)^+^. ^1^H NMR (CDCl_3_): δ 9.09 (s, 1H), 7.74 (d, *J* = 8.8 Hz, 1H), 7.30 (d, *J* = 8.8 Hz, 1H), 6.63 (s, 1H), 3.98 (t, *J* = 4.8 Hz, 2H), 3.72 (dd, *J* = 12.4, 3.6 Hz, 1H),3.60 (t, *J* = 4.8 Hz, 1H), 3.32–3.40 (m, 2H), 3.15 (dd, *J* = 12.4, 3.6 Hz, 1H), 2.39 (s, 3H), 2.33 (s, 6H), 2.20 (s, 6H), 1.02 (d, *J* = 6.8 Hz, 3H).

#### Ethyl (*R*)-7-(7-Chloro-10-(3-(4-chloro-3,5-dimethylphenoxy)propyl)-6-(4,6-dimethylpyrimidin-5-yl)-4-methyl-1-oxo-3,4-dihydropyrazino[1,2-*a*]indol-2(1*H*)-yl)-5-hydroxy-4-methoxy-1-methyl-1*H*-indole-2-carboxylate (**48**)

Compound **44** (1.0 g, 1.86 mmol, 1.0 equiv) and compound **45** (978 mg, 2.42 mmol, 1.3 equiv) were coupled following General Procedure C (1.51 g, 94% yield), LCMS Method 2: *R*_T_ = 1.972 min, MS (ESI): *m*/*z* 860.2 (M + H)^+^. The resultant compound (**46**) was alkylated with MeI following General Procedure D (1.22 g, 80% yield), LCMS Method 2: *R*_T_ = 2.303, 2.372 min, MS (ESI): *m*/*z* 874.1 (M + H)^+^. The resultant compound (**47**) was hydrogenated following General Procedure E to afford compound **48** (970 mg, 88% yield), LCMS Method 2: *R*_T_ = 1.743, 1.819 min, MS (ESI): *m*/*z* 784.2 (M + H)^+^. ^1^H NMR (CDCl_3_): δ (s, 1H), 7.855 (d, *J* = 8.8 Hz, 0.3H), 7.850 (d, *J* = 8.4 Hz, 0.7H), 7.40–7.34 (m, 2H), 6.81 (s, 0.7H), 6.80 (s, 0.3H), 6.62 (s, 1.4H), 6.60 (s, 0.6H), 4.39–4.33 (m, 2H), 4.20 (dd, *J* = 13.6, 4.0 Hz, 1H), 4.17–4.08 (m, 4H), 4.00–3.97 (m, 4H), 3.57–3.53 (m, 1H), 3.39–3.33 (m, 2H), 2.59 (s, 1H), 2.57 (s, 2H), 2.41 (s, 1H), 2.40 (s, 2H), 2.32 (s, 6H), 2.19–2.15 (m, 2H), 1.40 (t, *J* = 6.8 Hz, 3H), 1.28 (d, *J* = 7.2 Hz, 2H), 1.20 (d, *J* = 6.8 Hz, 1H).

#### (*R*)-7-Chloro-10-(3-(4-chloro-3,5-dimethylphenoxy)propyl)-4-methyl-6-(2,4,6-trimethylpyrimidin-5-yl)-3,4-dihydropyrazino[1,2-*a*]indol-1(2*H*)-one (**59**)

In a reaction vessel, compound **39** (6.75 g, 13.5 mmol), compound **54** (14.0 g, 39.5 mmol), 2-dicyclohexylphosphino-2′,6′-dimethoxybiphenyl (S-Phos, 2.44 g, 5.94 mmol), Pd_2_(dba)_3_ (1.81 g, 1.98 mmol), and K_2_CO_3_ (8.2 g, 59.4 mmol) were massed. Toluene (80 mL) and THF (80 mL) were added and the reaction mixture was sparged with argon for 5 min. The reaction was then sealed and heated to 110 °C for 4 h. The reaction mixture was cooled to room temperature and diluted into EtOAc/H_2_O. The mixture was extracted with EtOAc, washed with H_2_O, washed with brine, dried over MgSO_4_, filtered, and concentrated. The crude residue was purified by flash column chromatography eluting with 0 to 40% EtOAc in hexanes to afford the title compound (8.08 g, 63% yield). To a round-bottomed flask compound **55** (4.47 g, 6.9 mmol, 1.0 equiv), *tert*-butyl (*S*)-5-methyl-1,2,3-oxathiazolidine-3-carboxylate 2,2-dioxide (**42**, 2.46 g, 10.4 mmol), and Cs_2_CO_3_ (3.38 g, 10.4 mmol). Anhydrous MeCN (80 mL) was added and the solution was stirred at 80 °C overnight. The reaction mixture was diluted with EtOAc, and washed with brine, dried over MgSO_4_, filtered, and then concentrated (**56**). Compound **56** was then dissolved in DCM (80 mL) and cooled to 0 °C. TFA (8 mL) was added dropwise and the reaction mixture was allowed to stir at room temperature for 4 h. The solvent was removed *in vacuo*. Anhydrous EtOH (80 mL) was added followed by K_2_CO_3_ (23 g, 167 mmol). The reaction mixture was stirred at 50 °C for 2 h. The reaction mixture was concentrated to 1/3 volume and diluted with ethyl acetate (100 mL) and washed with brine (3 × 50 mL). The combined organic layers were dried over MgSO_4_, filtered, and concentrated. The crude residue was purified by flash column chromatography eluting with 0 to 5% MeOH in DCM to afford **57** (2.6 g, 84% yield). Compound **57** (2.6 g, 3.95 mmol, 1.0 equiv) was dissolved in MeOH (40 mL) and Pd/C (10 wt %, 420 mg, 0.39 mmol, 0.1 equiv) was added. The reaction was stirred at room temperature for 24 h under an atmosphere of hydrogen. The reaction was filtered through a pad of Celite, rinsed with DCM, and concentrated (2.20 g, 97% yield). LCMS Method 1: *R*_T_ = 1.969 min, MS (ESI): *m*/*z* 567.0 (M + H)^+^. The resultant material (2.2 g, 3.88 mmol, 1.0 equiv) was dissolved in DCM (40 mL) at 0 °C, followed by addition of MsCl (666 mg, 5.81 mmol, 1.5 equiv) and TEA (785 mg, 7.75 mmol, 2.0 equiv). The reaction was allowed to stir for 1 h at room temperature, followed by extraction with DCM, washed with H_2_O, dried over MgSO_4_, filtered, and concentrated to afford the crude product (2.5 g, quant. yield), LCMS Method 1: *R*_T_ = 2.131 min, MS (ESI): *m*/*z* 644.8 (M + H)^+^. The resultant product was taken up in THF (40 mL) and cooled to −40 °C. LiBHEt_3_ (1.0 M, 11.6 mL, 11.6 mmol, 3.0 equiv) was added and the reaction was allowed to stir at 0 °C for 5 h. The reaction was quenched with H_2_O, extracted with EtOAc, washed with H_2_O, dried over MgSO_4_, filtered, and concentrated. The crude residue was purified by flash column chromatography eluting with 0 to 5% MeOH in DCM to afford **59** (1.6 g, 75% yield), LCMS Method 1: *R*_T_ = 2.090 min, MS (ESI): *m*/*z* 550.9 (M + H)^+^. ^1^H NMR (CDCl_3_) δ 7.71 (d, *J* = 8.8 Hz, 1H), 7.28 (d, *J* = 8.4 Hz, 1H), 6.63 (s, 2H), 5.74 (d, *J* = 5.2 Hz, 1H), 4.00 (td, *J* = 6.4, 1.6 Hz, 2H), 3.75–3.69 (m, 2H), 3.42–3.30 (m, 2H), 3.15 (dd, *J* = 11.2, 5.6 Hz, 1H), 2.79 (s, 3H), 2.33 (s, 9H), 2.19 (quint, *J* = 7.2 Hz, 2H), 2.14 (s, 3H), 1.02 (d, *J* = 6.8 Hz, 3H).

#### Ethyl (*R*)-7-(7-Chloro-10-(3-(4-chloro-3,5-dimethylphenoxy)propyl)-4-methyl-1-oxo-6-(2,4,6-trimethylpyrimidin-5-yl)-3,4-dihydropyrazino[1,2-*a*]indol-2(1*H*)-yl)-5-hydroxy-4-methoxy-1-methyl-1*H*-indole-2-carboxylate (**60**)

Compound **59** (1.1 g, 2.05 mmol, 1.0 equiv) and compound **45** (1.08 g, 2.66 mmol, 1.3 equiv) were coupled following General Procedure C (1.61 g, 91% yield), LCMS Method 2: *R*_T_ = 1.870 min, MS (ESI): *m*/*z* 874.3 (M + H)^+^. The resultant compound was alkylated with MeI following General Procedure D (1.43 g, 85% yield), LCMS Method 2: *R*_T_ = 1.979, 2.025 min, MS (ESI): *m*/*z* 888.2 (M + H)^+^. The resultant compound was hydrogenated following General Procedure E to afford compound **60** (1.07 g, 83% yield), LCMS Method 2: *R*_T_ = 1.639, 1.703 min, MS (ESI): *m*/*z* 798.2 (M + H)^+^. ^1^H NMR (CDCl_3_): δ (d, *J* = 8.8 Hz, 0.3H), 7.845 (d, *J* = 8.8 Hz, 0.7H), 7.375 (d, *J* = 8.8 Hz, 0.3H), 7.365 (d, *J* = 8.8 Hz, 0.7H), 7.30 (s, 0.3H), 7.26 (s, 0.7H), 6.79 (s, 0.7H), 6.77 (s, 0.3H), 6.62 (s, 1.4H), 6.60 (s, 0.6H), 4.38–4.32 (m, 2H), 4.19–4.05 (m, 5H), 3.99–3.95 (m, 4H), 3.50–3.47 (m, 1H), 3.39–3.31 (m, 2H), 2.97 (s, 1H), 2.96 (s, 2H), 2.61 (s, 1H), 2.58 (s, 2H), 2.41 (s, 3H), 2.32 (s, 6H), 2.21–2.15 (m, 2H), 1.41–1.37 (m, 3H), 1.27 (d, *J* = 6.4 Hz, 2H), 1.19 (d, *J* = 6.4 Hz, 1H).

### Synthesis of Examples

#### (*R*)-7-(7-Chloro-10-(3-(4-chloro-3,5-dimethylphenoxy)propyl)-4-methyl-1-oxo-6-(1,3,5-trimethyl-1*H*-pyrazol-4-yl)-3,4-dihydropyrazino[1,2-*a*]indol-2(1*H*)-yl)-1-methyl-1*H*-indole-2-carboxylic Acid (**10**)

The title compound (16 mg, 45%) was prepared following General Procedure A using compound **33** (27 mg, 0.05 mmol) and ethyl 7-bromo-1-methyl-1*H*-indole-2-carboxylate (14 mg, 0.06 mmol) followed by saponification using General Procedure G. LCMS Method 2: *R*_T_ = 1.391, 1.435 min, MS (ESI): mass calcd for C_39_H_39_Cl_2_N_5_O_4_, 711.2, *m*/*z* found, 712.2 (M + H)^+^. ^1^H NMR (400 MHz, DMSO-*d*_6_) δ 7.75 (d, *J* = 8.4 Hz, 1H), 7.48 (d, *J* = 7.6 Hz, 0.7H), 7.43 (d, *J* = 7.6 Hz, 0.3H), 7.31–7.28 (m, 1H), 7.02–6.99 (m, 1H), 6.96–6.92 (m, 0.3H), 6.87–6.85 (m, 0.7H), 6.72 (s, 1H), 6.68–6.65 (m, 2H), 4.61–4.57 (m, 0.3H), 4.33–4.28 (m, 0.7H), 4.24–4.15 (m, 1H), 4.13 (s, 1H), 4.03 (s, 2H), 3.98–3.95 (m, 2H), 3.77 (d, *J* = 3.2 Hz, 2H), 3.75 (s, 1H), 3.71–3.61 (m, 1H), 3.28–3.15 (m, 3H), 2.24 (s, 6H), 2.12–2.11 (m, 1H), 2.07–1.98 (m, 6H), 1.92–1.89 (m, 1H), 1.16 (d, *J* = 6.4 Hz, 2H), 1.06 (d, *J* = 6.4 Hz, 1H), mixture of rotamers.

#### (*R*)-7-(7-Chloro-10-(3-(4-chloro-3,5-dimethylphenoxy)propyl)-4-methyl-1-oxo-6-(1,3,5-trimethyl-1*H*-pyrazol-4-yl)-3,4-dihydropyrazino[1,2-*a*]indol-2(1*H*)-yl)-1,5-dimethyl-1*H*-indole-2-carboxylic Acid (**11**)

The title compound (11 mg, 9% yield) was prepared following General Procedure B using compound **33** (90 mg, 0.17 mmol, 1.0 equiv) and methyl 7-bromo-1,5-dimethyl-1*H*-indole-2-carboxylate (96 mg, 0.34 mmol, 2.0 equiv) followed by saponification using General Procedure G. LCMS Method 1: *R*_T_ = 2.412, 2.450 min, MS (ESI): mass calcd for C_40_H_41_Cl_2_N_5_O_4_, 725.3, *m*/*z* found, 726.30 (M + H)^+^. ^1^H NMR (400 MHz, DMSO-*d*_6_) δ 7.75 (d, *J* = 8.4 Hz, 1H), 7.30 (t, *J* = 4.0 Hz, 1H), 7.28–7.27 (m, 1H), 7.22 (br. s, 0.5H), 6.88 (br. s, 0.5H), 6.72 (s, 1H), 6.69 (s, 1H), 6.60–6.58 (m, 1H), 4.61–4.57 (m, 0.4H), 4.32–4.26 (m, 0.6H), 4.24–4.12 (m, 1H), 4.10 (s, 1H), 3.99 (s, 2H), 3.97–3.95 (m, 2H), 3.78 (d, *J* = 2.8 Hz, 2H), 3.75 (s, 1H), 3.69–3.59 (m, 1H), 3.25–3.19 (m, 3H), 3.17 (s, 1H), 2.34 (s, 1.8H), 2.32 (s, 1.2H), 2.24 (s, 6H), 2.12–2.11 (m, 1H), 2.07–1.97 (m, 5H), 1.92–1.89 (m, 1H), 1.16 (d, *J* = 6.0 Hz, 2H), 1.07–1.05 (m, 1H), mixture of rotamers.

#### (*R*)-7-(7-Chloro-10-(3-(4-chloro-3,5-dimethylphenoxy)propyl)-4-methyl-1-oxo-6-(1,3,5-trimethyl-1*H*-pyrazol-4-yl)-3,4-dihydropyrazino[1,2-*a*]indol-2(1*H*)-yl)-5-methoxy-1-methyl-1*H*-indole-2-carboxylic Acid (**12**)

Compound **33** (1.20 g, 2.1 mmol, 1.0 equiv) and ethyl 5-(benzyloxy)-7-bromo-1*H*-indole-2-carboxylate (1.8 g, 4.4 mmol, 2.0 equiv) were coupled following General Procedure A (1.16 g, 63% yield). The resultant compound was methylated following General Procedure D and the benzyl ether cleaved following General Procedure E to afford the *N*-methyl phenol (83% yield over 2 steps). The resultant phenol was alkylated following General Procedure F using MeI (6 mg, 0.08 mmol, 2.0 equiv) and then the ester was saponified following General Procedure G to afford **12** (13.6 mg, 69% yield). LCMS Method 1: *R*_T_ = 2.028 min, MS (ESI): mass calcd for C_40_H_41_Cl_2_N_5_O_5_, 741.3, *m*/*z* found, 742.0 (M + H)^+^. ^1^H NMR (400 MHz, CDCl_3_) δ 7.73 (d, *J* = 8.6 Hz, 1H), 7.40–7.29 (m, 2H), 7.10 (d, *J* = 2.3 Hz, 0.66H), 7.06 (d, *J* = 2.3 Hz, 0.34H), 6.88–6.83 (m, 1H), 6.63 (s, 1.3H), 6.61 (s, 0.7H), 4.47–4.18 (m, 2H), 4.06–3.95 (m, 7H), 3.91 (s, 0.7H), 3.90 (s, 0.3H), 3.87 (s, 2H), 3.86 (s, 1H), 3.58 (d, *J* = 12.2 Hz, 0.3H),3.53 (d, *J* = 12.2 Hz, 0.7H), 3.45–3.30 (m, 2H), 2.32 (s, 6H), 2.28 (s, 3H), 2.22–2.15 (m, 2H), 2.10 (s, 1H), 2.08 (s, 2H), 1.30 (d, *J* = 6.5 Hz, 2H), 1.22 (d, *J* = 6.6 Hz, 1H), mixture of rotamers.

#### (*R*)-7-(7-Chloro-10-(3-(4-chloro-3,5-dimethylphenoxy)propyl)-4-methyl-1-oxo-6-(1,3,5-trimethyl-1*H*-pyrazol-4-yl)-3,4-dihydropyrazino[1,2-*a*]indol-2(1*H*)-yl)-4-methoxy-1-methyl-1*H*-indole-2-carboxylic Acid (**13**)

The title compound (11 mg, 30%) was prepared following General Procedure B using compound **33** (27 mg, 0.05 mmol) and methyl 7-bromo-4-methoxy-1-methyl-1*H*-indole-2-carboxylate (18 mg, 0.06 mmol followed by saponification using General Procedure G. LCMS Method 2: *R*_T_ = 1.423, 1.47 min, MS (ESI): mass calcd for C_40_H_41_Cl_2_N_5_O_5_, 741.3, *m*/*z* found, 742.0 (M + H). ^1^H NMR (400 MHz, DMSO-d6) δ 7.74 (d, *J* = 8.4 Hz, 1H), 7.31–7.27 (m, 1H), 6.92 (d, *J* = 8.4 Hz, 0.3H), 6.79–6.77 (m, 0.7H), 6.73 (s, 1H), 6.68–6.66 (m, 2H), 6.49 (d, *J* = 8.0 Hz, 0.7H), 6.43 (d, *J* = 8.0 Hz, 0.3H), 4.56–4.52 (m, 0.3H), 4.30–4.26 (m, 0.7H), 4.22–4.14 (m, 1H), 4.12 (s, 1H), 4.01 (s, 2H), 3.98–3.93 (m, 2H), 3.87 (s, 3H), 3.78–3.77 (m, 2H), 3.75–3.55 (m, 2H), 3.28–3.15 (m, 4H), 2.24 (s, 6H), 2.12–2.11 (m, 2H), 2.07–1.97 (m, 4H), 1.91–1.88 (m, 1H), 1.15 (d, *J* = 6.4 Hz, 2H), 1.05 (d, *J* = 6.4 Hz, 1H), mixture of rotamers.

#### (*R*)-7-(7-Chloro-10-(3-(4-chloro-3,5-dimethylphenoxy)propyl)-4-methyl-1-oxo-6-(1,3,5-trimethyl-1*H*-pyrazol-4-yl)-3,4-dihydropyrazino[1,2-*a*]indol-2(1*H*)-yl)-5-(methoxymethyl)-1-methyl-1*H*-indole-2-carboxylic Acid (**14**)

Compound **33** (510 mg, 0.94 mmol, 1.0 equiv) and ethyl 7-iodo-4-methoxy-1,5-dimethyl-1*H*-indole-2-carboxylate (335 mg, 0.94 mmol, 1.0 equiv) were coupled following General Procedure B (400 mg, 52% yield). The resultant product was dissolved in THF/MeOH (15 mL, 2:1) and concentrated HCl (36 drops) were added and the reaction was allowed to stir for 3 h at room temperature. The reaction was treated with aq. NaHCO_3_, extracted with DCM, and concentrated (280 mg, 75% yield). The product from this step was dissolved in DMF, treated with NaH, followed by MeI at room temperature. Upon completion, the crude material was extracted with DCM, dried, and concentrated. The resultant compound was saponified following General Procedure G (7 mg, 66% yield over 2 steps). LCMS Method 1: *R*_T_ = 2.159, 2.193 min, MS (ESI): mass calcd for C_41_H_43_Cl_2_N_5_O_5_, 755.3, *m*/*z* found, 755.8 (M + H)^+^. ^1^H NMR (400 MHz, CDCl_3_) δ 7.70 (dd, *J* = 8.4, 2.4 Hz, 1H), 7.65 (d, *J* = 4.0 Hz, 0.7H), 7.59 (d, *J* = 6.0 Hz, 0.3H), 7.43–7.42 (m, 1H), 7.32–7.29 (m, 1H), 7.19–7.17 (m, 0.3H), 7.13 (dd, *J* = 4.4, 1.2 Hz, 0.7H), 6.61 (s, 1.4H), 6.59 (s, 0.6H), 4.56–4.48 (m, 2H), 4.30–4.19 (m, 2H), 4.05 (d, *J* = 2.0 Hz, 2H), 4.00–3.96 (m, 2H), 3.93 (s, 2H), 3.91 (s, 1H), 3.64–3.57 (m, 1H), 3.42–3.30 (m, 5H), 2.30 (s, 6H), 2.25–2.23 (m, 3H), 2.21–2.16 (m, 2H), 2.11–2.06 (m, 4H), 1.32 (d, *J* = 6.8 Hz, 1.2H), 1.28 (d, *J* = 6.8 Hz, 0.6H), 1.20 (d, *J* = 6.8 Hz, 0.6H), 1.14 (d, *J* = 6.8 Hz, 0.6H), mixture of rotamers.

#### (*R*)-7-(7-Chloro-10-(3-(4-chloro-3,5-dimethylphenoxy)propyl)-4-methyl-1-oxo-6-(1,3,5-trimethyl-1*H*-pyrazol-4-yl)-3,4-dihydropyrazino[1,2-*a*]indol-2(1*H*)-yl)-5-methoxy-1,4-dimethyl-1*H*-indole-2-carboxylic Acid (**15**)

Compound **33** (60 mg, 0.11 mmol, 1.0 equiv) and ethyl 7-bromo-5-methoxy-4-methyl-1*H*-indole-2-carboxylate (70 mg, 0.22, 2.0 equiv) were coupled following General Procedure C (55 mg, 64% yield). The resultant product was alkylated with MeI following General Procedure D (44 mg, 80% yield)). The resultant compound was saponified following General Procedure G (28 mg, 66% yield). LCMS Method 1: *R*_T_ = 2.271 min, MS (ESI): mass calcd for C_41_H_43_Cl_2_N_5_O_5_, 755.3, *m*/*z* found, 755.8 (M + H)^+^. ^1^H NMR (400 MHz, CDCl_3_) δ 7.73 (d, *J* = 8.8 Hz, 0.7H), 7.72 (d, *J* = 8.8 Hz, 0.3H), 7.42 (d, *J* = 7.6 Hz, 1H), 7.33–7.30 (m, 1H), 6.87 (d, *J* = 6.8 Hz, 0.3H), 6.82 (d, *J* = 9.2 Hz, 0.7H), 6.61 (s, 1.4H), 6.59 (s, 0.6H), 4.54–4.48 (m, 0.3H), 4.29–4.14 (m, 2.7H), 4.01–3.96 (m, 7H), 3.84 (s, 0.5H), 3.82 (s, 2.5H), 3.62 (m, 2H), 3.43–3.30 (m, 1H), 2.43 (s, 0.7H), 2.42 (s, 2.3H), 2.30 (s, 6H), 2.27–2.45 (m, 3H), 2.19–2.10 (m, 5H), 1.32 (d, *J* = 6.4 Hz, 1.2H), 1.26 (d, *J* = 6.4 Hz, 1.2H), 1.21 (d, *J* = 6.8 Hz, 0.3H), 1.15 (d, *J* = 6.8 Hz, 0.3H), mixture of rotamers.

#### (*R*)-7-(7-Chloro-10-(3-(4-chloro-3,5-dimethylphenoxy)propyl)-4-methyl-1-oxo-6-(1,3,5-trimethyl-1*H*-pyrazol-4-yl)-3,4-dihydropyrazino[1,2-*a*]indol-2(1*H*)-yl)-4-methoxy-1,5-dimethyl-1*H*-indole-2-carboxylic Acid (**16**)

The title compound was prepared by coupling compound **33** (50 mg, 0.09 mmol, 1.0 equiv) and ethyl 7-iodo-4-methoxy-5-methyl-1*H*-indole-2-carboxylate (100 mg, 0.28 mmol, 3.0 equiv) following General Procedure A (48 mg, 67% yield). The resultant product was *N*-methylated using General Procedure D followed by saponification following General Procedure G (30 mg, 64% yield over 2 steps). LCMS Method 1: *R*_T_ = 2.178, 2.218 min, MS (ESI): mass calcd for C_41_H_43_Cl_2_N_5_O_5_, 755.3, *m*/*z* found, 755.90 (M + H)^+^. ^1^H NMR (400 MHz, CDCl_3_) δ 7.75 (d, *J* = 8.8 Hz, 0.7H), 7.74 (d, *J* = 8.4 Hz, 0.3H), 7.54 (s, 0.3H), 7.53 (s, 0.7H), 7.34–7.30 (m, 1H), 7.00–6.92 (m, 1H), 6.61 (s, 1.4H), 6.59 (0.6H), 4.45 (dd, *J* = 12.4, 4.0 Hz, 1H), 4.28–4.14 (m, 7H), 4.01–3.94 (m, 10H), 3.62–3.55 (m, 1H), 3.42–3.30 (m, 2H), 2.30 (s, 6H), 2.29–2.72 (m, 2H), 2.20–2.10 (m, 5H), 1.31 (d, *J* = 6.4 Hz, 1.7H), 1.25 (d, *J* = 3.2 Hz, 0.4H), 1.21 (d, *J* = 6.4 Hz, 0.6H), 1.14 (d, *J* = 6.4 Hz, 0.3H), mixture of rotamers.

#### (*R*)-7-(7-Chloro-10-(3-(4-chloro-3,5-dimethylphenoxy)propyl)-4-methyl-1-oxo-6-(1,3,5-trimethyl-1*H*-pyrazol-4-yl)-3,4-dihydropyrazino[1,2-*a*]indol-2(1*H*)-yl)-4-methoxy-5-(methoxymethyl)-1-methyl-1*H*-indole-2-carboxylic Acid (**17**)

Compound **33** (100 mg, 0.185 mmol, 1.0 equiv) and ethyl 7-iodo-4-methoxy-5-((methoxymethoxy)methyl)-1*H*-indole-2-carboxylate (194 mg, 0.46 mmol, 2.5 equiv, see Supporting Information (SI) for synthesis) were coupled following General Procedure B, followed by alkylation with MeI following General Procedure D. The resultant compound was then dissolved in THF/MeOH (2 mL, 2:1), conc. HCl (2.4 mL) was added, and the reaction was allowed to stir for 1 h. The reaction was quenched with aq. NaHCO_3_, extracted with DCM, and concentrated. The crude residue was saponified following General Procedure G to afford the title compound (30 mg, 51% yield over 2 steps). LCMS Method 2: *R*_T_ = 1.277, 1.345 min, MS (ESI): mass calcd for C_42_H_45_Cl_2_N_5_O_6_, 785.3, *m*/*z* found, 785.80 (M + H)^+^.

#### (*M*,*R*)-7-(7-Chloro-10-(3-(4-chloro-3,5-dimethylphenoxy)propyl)-4-methyl-1-oxo-6-(1,3,5-trimethyl-1*H*-pyrazol-4-yl)-3,4-dihydropyrazino[1,2-*a*]indol-2(1*H*)-yl)-4,5-dimethoxy-1-methyl-1*H*-indole-2-carboxylic Acid (*M*-**18**)

Compound (*M*)-**34** (730 mg, 1.35 mmol, 1.0 equiv) and ethyl 7-iodo-4,5-dimethoxy-1*H*-indole-2-carboxylate (1.27 g, 3.38 mmol, 2.5 equiv) were coupled following General Procedure A (690 mg, 65% yield). The resultant compound was *N*-alkylated using General Procedure D followed by saponification using General Procedure G to afford the title compound (500 mg, 74% yield). LCMS Method 1: *R*_T_ = 1.935 min, MS (ESI): mass calcd for C_41_H_43_Cl_2_N_5_O_6_, 771.3, *m*/*z* found, 771.9 (M + H)^+^. ^1^H NMR (400 MHz, DMSO-*d*_6_) δ 7.71 (d, *J* = 8.4 Hz, 0.3H), 7.70 (d, *J* = 8.8 Hz, 0.7H), 7.24 (d, *J* = 8.4 Hz, 0.3H), 7.23 (d, *J* = 8.8 Hz, 0.7H), 7.13 (s, 0.3H), 7.02 (s, 1H), 6.73 (s, 0.7H), 6.65 (s, 1.4H), 6.62 (s, 0.6H), 4.63 (dd, *J* = 12.8, 4.4 Hz, 0.3H), 4.24 (dd, *J* = 13.6, 3.6 Hz, 0.7H), 4.17–4.12 (m, 1H), 3.98 (s, 1H), 3.92–3.83 (m, 7H), 3.72 (s, 6H), 3.67–3.64 (m, 1H), 3.26–3.12 (m, 2H), 2.17 (s, 4H), 2.16 (s, 2H), 2.06 (s, 1H), 2.05 (s, 2H), 2.02–1.95 (m, 2H), 1.85 (s, 1H), 1.82 (s, 2H), 1.13 (d, *J* = 6.4 Hz, 2H), 0.98 (d, *J* = 6.4 Hz, 1H), mixture of rotamers. **Rac-18:**^1^H NMR (400 MHz, DMSO-d6) δ 7.75 (d, *J* = 8.4 Hz, 1H), 7.31–7.28 (m, 1H), 6.99 (s, 0.5H), 6.73–6.34 (m, 3.5H), 4.68–4.63 (m, 0.5H), 4.31–4.26 (m, 0.5H), 4.23–4.11 (m, 1H), 4.06 (s, 1H), 4.02–3.92 (m, 4H), 3.88 (s, 2H), 3.87 (s, 1H), 3.78 (s, 2H), 3.76 (s, 3H), 3.71–3.61 (m, 1H), 3.25–3.15 (m, 3H), 2.24 (s, 6H), 2.13–2.11 (m, 2H), 2.04–1.97 (m, 5H), 1.92–1.89 (m, 2H), 1.18 (d, *J* = 6.0 Hz, 2H), 1.05 (d, *J* = 6.4 Hz, 1H), mixture of rotamers.

#### (*R*)-7-(7-Chloro-10-(3-(4-chloro-3,5-dimethylphenoxy)propyl)-6-(4,6-dimethylpyrimidin-5-yl)-4-methyl-1-oxo-3,4-dihydropyrazino[1,2-*a*]indol-2(1*H*)-yl)-4,5-dimethoxy-1-methyl-1*H*-indole-2-carboxylic Acid (**19**)

Compound **44** (200 mg, 0.37 mmol, 1.0 equiv) and methyl 7-iodo-4,5-dimethoxy-1*H*-indole-2-carboxylate (336 mg, 0.93 mmol, 2.5 equiv) were coupled following General Procedure C (194 mg, 68% yield). The resultant compound (194 mg, 0.25 mmol) was treated with MeI (89 mg, 0.63 mmol, 2.5 equiv) following General Procedure D, followed by saponification following General Procedure G to afford the title compound (165 mg, 85% yield over 2 steps). LCMS Method 1: *R*_T_ = 2.146 min, MS (ESI): mass calcd for C_41_H_41_Cl_2_N_5_O_6_, 769.2, *m*/*z* found, 769.8 (M + H)^+^. ^1^H NMR (400 MHz, DMSO-*d*_6_) δ 9.09 (s, 1H), 7.92 (d, *J* = 8.4 Hz, 0.3H), 7.90 (d, *J* = 8.8 Hz, 0.7H), 7.44–7.40 (m, 1H), 7.16 (s, 0.3H), 7.09 (s, 1H), 6.81 (s, 0.7H), 6.73 (s, 1.4H), 6.70 (s, 0.6H), 4.68 (dd, *J* = 12.8, 4.0 Hz, 0.3H), 4.32 (dd, *J* = 13.6, 3.6 Hz, 0.7H), 4.03–3.98 (m, 3H), 3.92–3.90 (m, 5H), 3.79 (s, 2H), 3.78 (s, 1H), 3.72–3.60 (m, 2H), 3.37–3.19 (m, 2H), 2.32 (s, 1H), 2.27 (s, 2H), 2.25 (s, 4H), 2.24 (s, 2H), 2.20 (s, 2H), 2.19 (s, 1H), 2.09–2.05 (m, 2H), 1.22 (d, *J* = 6.4 Hz, 2H), 1.10 (d, *J* = 6.4 Hz, 1H), mixture of rotamers.

#### (*R*)-7-(7-Chloro-10-(3-(4-chloro-3,5-dimethylphenoxy)propyl)-4-methyl-1-oxo-6-(2,4,6-trimethylpyrimidin-5-yl)-3,4-dihydropyrazino[1,2-*a*]indol-2(1*H*)-yl)-4,5-dimethoxy-1-methyl-1*H*-indole-2-carboxylic Acid (**20**)

Compound **59** (300 mg, 0.54 mmol, 1.0 equiv) and ethyl 7-iodo-4,5-dimethoxy-1*H*-indole-2-carboxylate (408 mg, 1.09 mmol, 2.0 equiv) were coupled following General Procedure A (400 mg, 92% yield). The resultant compound was treated with MeI (106 mg, 0.75 mmol, 1.5 equiv) following General Procedure D, followed by saponification following General Procedure G to afford the title compound (230 mg, 58% yield over 2 steps). LCMS Method 1: *R*_T_ = 2.236 min, MS (ESI): mass calcd for C_42_H_43_Cl_2_N_5_O_6_, 783.3, *m*/*z* found, 783.9 (M + H)^+^. ^1^H NMR (400 MHz, DMSO-*d*_6_) δ 7.89 (d, *J* = 8.4 Hz, 0.3H), 7.87 (d, *J* = 8.4 Hz, 0.7H), 7.41–7.37 (m, 1H), 7.13 (s, 0.3H), 7.02 (s, 0.3H), 7.01 (s, 0.7H), 6.78 (s, 0.7H), 6.73 (s, 1.4H), 6.70 (s, 0.6H), 4.68 (dd, *J* = 12.4, 3.6 Hz, 0.3H), 4.31 (dd, *J* = 13.6, 3.6 Hz, 1H), 4.04 (s, 0.7H), 4.00 (t, *J* = 6.0 Hz, 2H), 3.92–3.90 (m, 5H), 3.81–3.73 (m, 4H), 3.67–3.60 (m, 1H), 3.37–3.17 (m, 3H), 2.66 (s, 3H), 2.27 (s, 1H), 2.25 (s, 4H), 2.24 (s, 1H), 2.21 (s, 2H), 2.14 (s, 2H), 2.13 (s, 1H), 2.08–2.03 (m, 2H), 1.23 (d, *J* = 6.8 Hz, 2H), 1.11 (d, *J* = 6.8 Hz, 1H), mixture of rotamers.

#### (*R*)-7-(7-Chloro-10-(3-(4-chloro-3,5-dimethylphenoxy)propyl)-6-(4,6-dimethylpyrimidin-5-yl)-4-methyl-1-oxo-3,4-dihydropyrazino[1,2-*a*]indol-2(1*H*)-yl)-4-methoxy-5-(2-methoxyethoxy)-1-methyl-1*H*-indole-2-carboxylic Acid (**21**)

The title compound (226 mg, 60% yield) was prepared following General Procedure F using compound **48** (365 mg, 0.47 mmol, 1.0 equiv) and 2-bromoethyl methyl ether (162 mg, 1.16 mmol, 2.5 equiv) followed by saponification using General Procedure G. LCMS Method 1: *R*_T_ = 2.213, 2.235 min, MS (ESI): mass calcd for C_43_H_45_Cl_2_N_5_O_7_, 813.3, *m*/*z* found, 813.9 (M + H)^+^. ^1^H NMR (400 MHz, CDCl_3_) δ 9.14 (s, 0.25H), 9.13 (s, 0.75H), 7.76 (d, *J* = 8.8 Hz, 1H), 7.45 (d, *J* = 8.8 Hz, 1H), 7.45 (br, s, 1H), 7.34 (dd, *J* = 8.8, 2.4 Hz, 1H), 6.89 (s, 1H), 6.60 (s, 1.5H), 6.58 (s, 0.5H), 4.36 (d, *J* = 8.0 Hz, 0.4H), 4.15–4.10 (m, 2.6H), 4.06 (s, 1H), 4.03 (s, 3H), 3.98–3.89 (m, 4H), 3.78–3.68 (m, 3H), 3.48–3.28 (m, 7H), 2.44 (s, 0.7H), 2.43 (s, 2.3H), 2.29 (s, 6H), 2.26 (s, 3H), 2.22–2.15 (m, 1H), 1.26 (d, *J* = 6.4 Hz, 2.4H), 1.15 (d, *J* = 6.4 Hz, 0.6H), mixture of rotamers.

#### (*R*)-7-(7-Chloro-10-(3-(4-chloro-3,5-dimethylphenoxy)propyl)-6-(4,6-dimethylpyrimidin-5-yl)-4-methyl-1-oxo-3,4-dihydropyrazino[1,2-*a*]indol-2(1*H*)-yl)-4-methoxy-1-methyl-5-((tetrahydro-2*H*-pyran-4-yl)methoxy)-1*H*-indole-2-carboxylic Acid (**22**)

The title compound (30 mg, 39% yield) was prepared following General Procedure F using compound **48** (70 mg, 0.089 mmol, 1.0 equiv) and (tetrahydro-2*H*-pyran-4-yl)methyl 4-methylbenzenesulfonate (75 mg, 0.27 mmol, 3.0 equiv) followed by saponification using General Procedure G. LCMS Method 2: *R*_T_ = 1.378 min, MS (ESI): mass calcd for C_46_H_49_Cl_2_N_5_O_7_, 853.3, *m*/*z* found 853.8 (M + H)^+^. ^1^H NMR (400 MHz, CDCl_3_) δ 9.14 (s, 1H), 7.78 (d, *J* = 8.8 Hz, 1H), 7.49 (s, 0.75H), 7.48 (s, 0.25H), 7.35 (d, *J* = 10.4 Hz, 1H), 6.83 (s, 0.25H), 6.82 (s, 0.75H), 6.61 (s, 1.5H), 6.59 (s, 0.5H), 4.48 (dd, *J* = 13.2, 3.6 Hz, 0.3H), 4.18 (dd, *J* = 13.2, 3.6 Hz, 0.9H), 4.13 (s, 0.8H), 4.03–3.97 (m, 9H), 3.87–3.74 (m, 2.3H), 3.73–3.68 (m, 0.7H), 3.49–3.32 (m, 5H), 2.46 (s, 2.3H), 2.44 (s, 0.7H), 2.30 (s, 6H), 2.29 (s, 0.7H), 2.28 (s, 2.3H), 2.23–2.16 (m, 1H), 2.09–2.04 (m, 1H), 1.76 (d, *J* = 8.0 Hz, 2H), 1.46 (dq, *J* = 12.0, 4.4 Hz, 2H), 1.28 (d, *J* = 6.4 Hz, 2.3H), 1.18 (d, *J* = 6.4 Hz, 0.7H), mixture of rotamers.

#### 7-((*R*)-7-Chloro-10-(3-(4-chloro-3,5-dimethylphenoxy)propyl)-6-(4,6-dimethylpyrimidin-5-yl)-4-methyl-1-oxo-3,4-dihydropyrazino[1,2-*a*]indol-2(1*H*)-yl)-4-methoxy-1-methyl-5-(((*S*)-tetrahydrofuran-2-yl)methoxy)-1*H*-indole-2-carboxylic Acid (**23**)

The title compound (34 mg, 45% yield) was prepared following General Procedure F using compound **48** (70 mg, 0.089 mmol, 1.0 equiv) and (*S*)-(tetrahydrofuran-2-yl)methyl 4-methylbenzenesulfonate (75 mg, 0.29 mmol, 3.2 equiv) followed by saponification using General Procedure G. LCMS Method 2: *R*_T_ = 1.353 min, MS (ESI): mass calcd for C_45_H_47_Cl_2_N_5_O_7_, 839.3, *m*/*z* found 839.8 (M + H)^+^. ^1^H NMR (400 MHz, CDCl_3_) δ 9.18 (s, 1H), 7.80 (d, *J* = 8.8 Hz, 1H), 7.49 (s, 0.3H), 7.48 (s, 0.7H), 7.34 (d, *J* = 8.8 Hz, 1H), 6.93–6.92 (M, 1H), 6.61 (s, 1.3H), 6.60 (s, 0.7H), 4.39 (d, *J* = 8.4 Hz, 0.3H), 4.30–4.23 (m, 1H), 4.17 (dd, *J* = 13.2, 3.6 Hz, 0.7H), 4.11 (s, 1H), 4.06 (s, 2H), 4.04 (s, 1H), 4.03–3.95 (m, 6H), 3.93–3.88 (m, 1H), 3.86–3.78 (m, 1H), 3.72–3.70 (m, 1H), 3.49 (d, *J* = 12.0 Hz, 1H), 3.42–3.31 (m, 2H), 2.49 (s, 1H), 2.47 (s, 2H), 2.32–2.30 (m, 9H), 2.22–2.14 (m, 2H), 2.09–2.01 (m, 1H), 1.98–1.89 (m, 2H), 1.82–1.71 (m, 1H), 1.28 (d, *J* = 6.4 Hz, 2H), 1.18 (d, *J* = 6.4 Hz, 1H), mixture of rotamers.

#### 7-((*R*)-7-Chloro-10-(3-(4-chloro-3,5-dimethylphenoxy)propyl)-6-(4,6-dimethylpyrimidin-5-yl)-4-methyl-1-oxo-3,4-dihydropyrazino[1,2-*a*]indol-2(1*H*)-yl)-4-methoxy-1-methyl-5-(((*R*)-tetrahydrofuran-2-yl)methoxy)-1*H*-indole-2-carboxylic Acid (**24**)

The title compound (32 mg, 43% yield) was prepared following General Procedure F using compound **48** (70 mg, 0.089 mmol, 1.0 equiv) and (*R*)-(tetrahydrofuran-2-yl)methyl 4-methylbenzenesulfonate (75 mg, 0.29 mmol, 3.2 equiv) followed by saponification using General Procedure G. LCMS Method 2: *R*_T_ = 1.376 min, MS (ESI): mass calcd for C_45_H_47_Cl_2_N_5_O_7_, 839.3, *m*/*z* found 839.8 (M + H)^+^. ^1^H NMR (400 MHz, CDCl_3_) δ 9.15 (s, 1H), 7.78 (d, *J* = 8.8 Hz, 1H), 7.49 (s, 0.3H), 7.47 (s, 0.7H), 7.35 (d, *J* = 8.8 Hz, 1H), 6.93 (s, 0.7H), 6.92 (s, 0.3H), 6.61 (s, 0.7H), 6.60 (s, 0.3H), 4.38 (dd, *J* = 12.8, 4.4 Hz, 0.3H), 4.29–4.22 (m, 1H), 4.16 (dd, *J* = 13.6, 4.0 Hz, 0.7H), 4.11 (s, 1H), 4.04 (s, 2H), 4.03 (s, 1H), 4.01–3.95 (m, 6H), 3.91–3.87 (m, 1H), 3.84 (m, 1H), 3.73–3.70 (m, 1H), 3.48 (d, *J* = 12.8 Hz, 1H), 3.41–3.31 (m, 2H), 2.47 (s, 1H), 2.45 (s, 2H), 2.30 (s, 8H), 2.28 (s, 1H), 2.22–2.14 (m, 2H), 2.08–2.02 (m, 1H), 1.97–1.89 (m, 2H), 1.78–1.70 (m, 1H), 1.29 (d, *J* = 7.6 Hz, 2H), 1.17 (d, *J* = 6.8 Hz, 1H), mixture of rotamers.

#### (*R*)-7-(7-Chloro-10-(3-(4-chloro-3,5-dimethylphenoxy)propyl)-4-methyl-1-oxo-6-(2,4,6-trimethylpyrimidin-5-yl)-3,4-dihydropyrazino[1,2-*a*]indol-2(1*H*)-yl)-4-methoxy-5-(2-methoxyethoxy)-1-methyl-1*H*-indole-2-carboxylic Acid (**25**)

The title compound (359 mg, 52% yield) was prepared following General Procedure F using compound **60** (750 mg, 0.94 mmol, 1.0 equiv) and 2-bromoethyl methyl ether (390 mg, 2.82 mmol, 3.0 equiv) followed by saponification using General Procedure G. LCMS Method 1: *R*_T_ = 2.219 min, MS (ESI): mass calcd for C_44_H_47_Cl_2_N_5_O_7_, 827.3, *m*/*z* found 827.8 (M + H)^+^. ^1^H NMR (400 MHz, CDCl_3_) δ 7.75 (d, *J* = 8.4 Hz, 1H), 7.49 (s, 0.25H), 7.47 (s, 0.75H), 7.33 (dd, *J* = 8.4, 2.0 Hz, 1H), 6.92 (s, 0.75H), 6.90 (s, 0.25H), 6.60 (s, 1.5H), 6.58 (s, 0.5H), 4.35 (dd, *J* = 8.8, 4.0 Hz, 0.4H), 4.20–4.10 (m, 3.6H), 4.05 (s, 2.3H), 4.04 (s, 0.7H), 4.00–3.96 (m, 4H), 3.81–3.76 (m, 1H), 3.72–3.69 (m, 2H), 3.49–3.46 (m, 1H), 3.42 (0.7H), 3.41 (s, 2.3H), 3.40–3.31 (m, 2H), 2.82 (s, 3H), 2.41 (s, 0.7H), 2.40 (s, 2.3H), 2.29 (s, 6H), 2.25 (s, 0.7H), 2.23 (s, 2.3H), 2.20–2.17 (m, 2H), 1.27 (d, *J* = 6.4 Hz, 2.3H), 1.18 (d, *J* = 6.0 Hz, 0.7H), mixture of rotamers.

#### (*R*)-7-(7-Chloro-10-(3-(4-chloro-3,5-dimethylphenoxy)propyl)-4-methyl-1-oxo-6-(2,4,6-trimethylpyrimidin-5-yl)-3,4-dihydropyrazino[1,2-*a*]indol-2(1*H*)-yl)-4-methoxy-1-methyl-5-((tetrahydro-2*H*-pyran-4-yl)methoxy)-1*H*-indole-2-carboxylic Acid (**26**)

The title compound (3.10 g, 61% yield) was prepared following General Procedure F using compound **60** (4.10 g, 5.10 mmol, 1.0 equiv) and (tetrahydro-2*H*-pyran-4-yl)methyl 4-methylbenzenesulfonate (4.20 g, 15 mmol, 3.0 equiv) followed by saponification using General Procedure G. The crude residue was purified by flash column chromatography eluting with 0 to 100% EtOAc in hexanes to afford the title compound. LCMS Method 1: *R*_T_ = 2.289 min, MS (ESI): mass calcd for C_47_H_51_Cl_2_N_5_O_7_, 867.3, *m*/*z* found 867.9 (M + H)^+^. ^1^H NMR (400 MHz, CDCl_3_) δ 7.76 (d, *J* = 8.8 Hz, 1H), 7.48 (s, 0.3H), 7.47 (s, 0.7H), 7.34 (d, *J* = 8.4 Hz, 1H), 6.82 (s, 0.3H), 6.81 (s, 0.7H), 6.60 (s, 1.4H), 6.58 (s, 0.6H), 4.37 (dd, *J* = 12.0, 4.0 Hz, 0.3H), 4.19 (dd, *J* = 13.2, 3.6 Hz, 0.7H), 4.11 (s, 1H), 4.02–3.94 (m, 9H), 3.87–3.78 (m, 3H), 3.51–3.35 (m, 3H), 3.34–3.28 (m, 2H), 2.82 (s, 1H), 2.81 (s, 2H), 2.42 (s, 1H), 2.41 (s, 2H), 2.30 (s, 6H), 2.25 (s, 1H), 2.23 (s, 2H), 2.20–2.17 (m, 2H), 2.09–2.02 (m, 1H), 1.76 (d, *J* = 12.4 Hz, 2H), 1.46 (dq, *J* = 12.4, 4.4 Hz, 2H), 1.29 (d, *J* = 6.4 Hz, 2H), 1.18 (d, *J* = 6.8 Hz, 1H), mixture of rotamers.

#### 7-((*R*)-7-Chloro-10-(3-(4-chloro-3,5-dimethylphenoxy)propyl)-6-(2,4,6-trimethylpyrimidin-5-yl)-4-methyl-1-oxo-3,4-dihydropyrazino[1,2-*a*]indol-2(1*H*)-yl)-4-methoxy-1-methyl-5-(((*S*)-tetrahydrofuran-2-yl)methoxy)-1*H*-indole-2-carboxylic Acid (**27**)

The title compound (40 mg, 58% yield) was prepared following General Procedure F using compound **60** (65 mg, 0.081 mmol, 1.0 equiv) and (*S*)-(tetrahydrofuran-2-yl)methyl 4-methylbenzenesulfonate (63 mg, 0.24 mmol, 3.0 equiv) followed by saponification using General Procedure G. LCMS Method 1: *R*_T_ = 2.295 min, MS (ESI): mass calcd for C_46_H_49_Cl_2_N_5_O_7_, 853.3, *m*/*z* found 853.9 (M + H)^+^. ^1^H NMR (400 MHz, CDCl_3_) δ 7.72 (d, *J* = 8.4 Hz, 0.3H), 7.71 (d, *J* = 8.4 Hz, 0.7H), 7.32–7.28 (m, 2H), 6.83 (s, 0.3H), 6.81 (s, 0.7H), 6.57 (s, 2H), 4.34 (dd, *J* = 12.4, 4.0 Hz, 0.3H), 4.22–4.18 (m, 1H), 4.12 (dd, *J* = 12.4, 4.0 Hz, 0.7H), 4.06 (s, 1H), 3.96–3.89 (m, 8H), 3.85 (q, *J* = 6.8 Hz, 2H), 3.81–3.74 (m, 2H), 3.44 (t, *J* = 11.2 Hz, 1H), 3.36–3.26 (m, 2H), 2.78 (s, 1H), 2.77 (s, 2H), 2.37 (s, 3H), 2.26 (s, 6H), 2.20 (s, 1H), 2.19 (s, 2H), 2.14 (t, *J* = 6.8 Hz, 2H), 2.05–1.99 (m, 1H), 1.93–1.87 (m, 2H), 1.74–1.67 (m, 1H), 1.27 (d, *J* = 6.4 Hz, 2H), 1.14 (d, *J* = 6.4 Hz, 1H), mixture of rotamers.

#### 7-((*R*)-7-Chloro-10-(3-(4-chloro-3,5-dimethylphenoxy)propyl)-6-(2,4,6-trimethylpyrimidin-5-yl)-4-methyl-1-oxo-3,4-dihydropyrazino[1,2-*a*]indol-2(1*H*)-yl)-4-methoxy-1-methyl-5-(((*R*)-tetrahydrofuran-2-yl)methoxy)-1*H*-indole-2-carboxylic Acid (**28**)

The title compound (29 mg, 42% yield) was prepared following General Procedure F using compound **60** (65 mg, 0.081 mmol, 1.0 equiv) and (*R*)-(tetrahydrofuran-2-yl)methyl 4-methylbenzenesulfonate (63 mg, 0.24 mmol, 3.0 equiv) followed by saponification using General Procedure G. LCMS Method 1: *R*_T_ = 2.301 min, MS (ESI): mass calcd for C_46_H_49_Cl_2_N_5_O_7_, 853.3, *m*/*z* found 853.9 (M + H)^+^. ^1^H NMR (400 MHz, CDCl_3_) δ 7.69 (d, *J* = 8.8 Hz, 0.3H), 7.68 (d, *J* = 8.8 Hz, 0.7H), 7.30–7.27 (m, 1H), 7.23 (s, 0.7H), 7.18 (s, 0.3H), 6.78 (s, 0.3H), 6.76 (s, 0.7H), 6.54 (s, 1.3H), 6.52 (s, 0.7H), 4.35 (dd, *J* = 9.2, 4.0 Hz, 0.3H), 4.22–4.15 (m, 1H), 4.08 (d, *J* = 10.4 Hz, 0.7H), 4.01 (s, 1H), 3.94–3.83 (m, 10H), 3.80–3.73 (m, 2H), 3.41 (t, *J* = 13.2 Hz, 1H), 3.32–3.26 (m, 2H), 2.77 (s, 1H), 2.76 (s, 2H), 2.35 (s, 3H), 2.23 (s, 4H), 2.22 (s, 2H), 2.20 (s, 1H), 2.17 (s, 3H), 2.14–2.10 (m, 2H), 2.04–1.96 (m, 1H), 1.68–1.62 (m, 1H), 1.24 (d, *J* = 6.4 Hz, 2H), 1.10 (d, *J* = 6.4 Hz, 1H), mixture of rotamers.

#### 7-((*R*)-7-Chloro-10-(3-(4-chloro-3,5-dimethylphenoxy)propyl)-4-methyl-1-oxo-6-(2,4,6-trimethylpyrimidin-5-yl)-3,4-dihydropyrazino[1,2-*a*]indol-2(1*H*)-yl)-4-methoxy-1-methyl-5-(((*S*)-tetrahydrofuran-3-yl)oxy)-1*H*-indole-2-carboxylic Acid (**29**)

The title compound (108 mg, 69% yield) was prepared following General Procedure F using compound **60** (150 mg, 0.19 mmol, 1.0 equiv) and (*R*)-tetrahydrofuran-3-yl 4-methylbenzenesulfonate (135 mg, 0.56 mmol, 3.0 equiv) followed by saponification using General Procedure G. LCMS Method 1: *R*_T_ = 2.203 min, MS (ESI): mass calcd for C_45_H_47_Cl_2_N_5_O_7_, 839.3, *m*/*z* found 839.8 (M + H)^+^. ^1^H NMR (400 MHz, DMSO-*d*_6_) δ 7.95 (d, *J* = 8.8 Hz, 0.3H), 7.94 (d, *J* = 8.8 Hz, 0.7H), 7.47–7.43 (m, 1H), 7.11 (s, 0.3H), 7.06 (s, 1H), 6.78 (s, 1.4H), 6.76 (s, 1.3H), 5.02–4.97 (m, 1H), 4.71 (dd, *J* = 12.8, 4.4 Hz, 0.3H), 4.35 (dd, *J* = 13.6, 3.2 Hz, 0.7H), 4.12 (s, 1H), 4.06–4.01 (m, 4H), 3.96–3.91 (m, 5H), 3.86–3.78 (m, 3H), 3.72–3.66 (m, 1H), 3.38–3.24 (m, 2H), 2.71 (s, 3H), 2.32 (s, 1H), 2.30 (s, 4H), 2.29 (s, 2H), 2.27 (s, 2H), 2.20 (s, 2H), 2.19 (s, 1H), 2.13–2.07 (m, 4H), 1.27 (d, *J* = 6.8 Hz, 2H), 1.16 (d, *J* = 6.8 Hz, 1H), mixture of rotamers.

#### 7-((*R*)-7-Chloro-10-(3-(4-chloro-3,5-dimethylphenoxy)propyl)-4-methyl-1-oxo-6-(2,4,6-trimethylpyrimidin-5-yl)-3,4-dihydropyrazino[1,2-*a*]indol-2(1*H*)-yl)-4-methoxy-1-methyl-5-(((*R*)-tetrahydrofuran-3-yl)oxy)-1*H*-indole-2-carboxylic Acid (**30**)

The title compound (59 mg, 86% yield) was prepared following General Procedure F using compound **60** (65 mg, 0.081 mmol, 1.0 equiv) and (*S*)-tetrahydrofuran-3-yl 4-methylbenzenesulfonate (59 mg, 0.24 mmol, 3.0 equiv) followed by saponification using General Procedure G. LCMS Method 1: *R*_T_ = 2.211 min, MS (ESI): mass calcd for C_45_H_47_Cl_2_N_5_O_7_, 839.3, *m*/*z* found 839.8 (M + H)^+^. ^1^H NMR (400 MHz, CDCl_3_) δ 7.75 (d, *J* = 8.8 Hz, 1H), 7.48 (s, 0.3H), 7.47 (s, 0.7H), 7.34 (d, *J* = 8.8 Hz, 0.3H), 7.33 (d, *J* = 8.4 Hz, 0.7H), 6.81 (s, 0.3H), 6.79 (s, 0.7H), 6.60 (s, 1.4H), 6.58 (s, 0.6H), 4.91–4.88 (m, 1H), 4.35 (dd, *J* = 13.2, 3.6 Hz), 0.3H), 4.16 (dd, *J* = 13.2, 3.6 Hz, 0.7H), 4.11 (s, 1H), 4.06–3.97 (m, 9H), 3.91–3.79 (m, 3H), 3.50–3.29 (m, 3H), 2.81 (s, 0.7H), 2.80 (s, 2.3H), 2.41 (s, 0.7H), 2.40 (s, 2.3H), 2.25–2.07 (m, 7H), 1.27 (d, *J* = 6.4 Hz, 2.3 H), 1.18 (d, *J* = 6.4 Hz, 0.7H); mixture of rotamers.

#### 7-((*R*)-7-Chloro-10-(3-(4-chloro-3,5-dimethylphenoxy)propyl)-4-methyl-1-oxo-6-(2,4,6-trimethylpyrimidin-5-yl)-3,4-dihydropyrazino[1,2-*a*]indol-2(1*H*)-yl)-4-ethoxy-1-methyl-5-(((*S*)-tetrahydrofuran-3-yl)oxy)-1*H*-indole-2-carboxylic Acid (**31**)

Compound **59** (500 mg, 0.91, 1.0 equiv) and ethyl 5-(benzyloxy)-4-ethoxy-7-iodo-1*H*-indole-2-carboxylate (720 mg, 1.54 mmol, 1.7 equiv) were coupled following General Procedure A (580 mg, 72% yield), LCMS Method 2: *R*_T_ = 1.676 min, MS (ES) 888.10 (M + H). The resultant product was alkylated with MeI following General Procedure D (550 mg, 93% yield), LCMS Method 2: *R*_T_ = 1.963, 2.020 min, MS (ES) 902.10 (M + H). The benzyl ether of the resultant product was cleaved following General Procedure E (quantitative yield), LCMS Method 2: *R*_T_ = 1.576, 1.648 min, MS (ES) 812.10 (M + H). The resultant 5-OH indole (70 mg, 0.86 mmol, 1.0 equiv) was alkylated with (*R*)-tetrahydrofuran-3-yl 4-methylbenzenesulfonate (63 mg, 0.26 mmol, 3.0 equiv) followed by saponification using General Procedure G (35 mg, 48% yield). LCMS Method 2: *R*_T_ = 1.446 min, MS (ESI): mass calcd for C_46_H_49_Cl_2_N_5_O_7_, 853.3, *m*/*z* found 853.8 (M + H). ^1^H NMR (400 MHz, CDCl_3_) δ 7.83 (d, *J* = 8.8 Hz, 1H), 7.49 (s, 1H), 7.37 (d, *J* = 8.8 Hz, 0.3H), 7.36 (d, *J* = 8.8 Hz, 0.7H), 6.84 (s, 0.3H), 6.80 (s, 0.7H), 6.61 (s, 1.4H), 6.59 (s, 0.6H), 4.95–4.92 (m, 1H), 4.42 (dd, *J* = 12.8, 4.0 Hz, 0.3H), 4.30–4.23 (m, 2H), 4.19 (dd, *J* = 13.6, 4.0 Hz, 0.7H), 4.12 (s, 1H), 4.08–3.97 (m, 6H), 3.95–3.89 (m, 1H), 3.87–3.79 (m, 2H), 3.55–3.50 (m, 1H), 3.43–3.30 (m, 2H), 2.94 (s, 1H), 2.92 (s, 2H), 2.55 (s, 1H), 2.52 (s, 2H), 2.35 (s, 3H), 3.21 (s, 6H), 2.20–2.15 (m, 3H), 2.10–2.03 (m, 1H), 1.39 (t, *J* = 7.2 Hz, 3H), 1.30 (d, *J* = 6.4 Hz, 2H), 1.19 (d, *J* = 6.4 Hz, 1H).

#### (*R*)-7-(7-Chloro-10-(3-(4-chloro-3,5-dimethylphenoxy)propyl)-4-methyl-1-oxo-6-(2,4,6-trimethylpyrimidin-5-yl)-3,4-dihydropyrazino[1,2-*a*]indol-2(1*H*)-yl)-4-ethoxy-1-methyl-5-((tetrahydro-2*H*-pyran-4-yl)methoxy)-1*H*-indole-2-carboxylic Acid (**32**)

The title compound (190 mg, 36% yield) was prepared following General Procedure F using ethyl (*R*)-7-(7-chloro-10-(3-(4-chloro-3,5-dimethylphenoxy)propyl)-4-methyl-1-oxo-6-(2,4,6-trimethylpyrimidin-5-yl)-3,4-dihydropyrazino[1,2-*a*]indol-2(1*H*)-yl)-4-ethoxy-5-hydroxy-1-methyl-1*H*-indole-2-carboxylate (495 mg, 0.61 mmol, 1.0 equiv) and (tetrahydro-2H-pyran-4-yl)methyl 4-methylbenzenesulfonate (494 mg, 1.83 mmol, 3.0 equiv) followed by saponification using General Procedure G. LCMS Method 2: *R*_T_ = 1.535 min, MS (ESI): mass calcd for C_48_H_53_Cl_2_N_5_O_7_, 881.3, *m*/*z* found 882.3 (M + H). ^1^H NMR (400 MHz, CDCl_3_) δ 7.84 (d, *J* = 8.8 Hz, 1H), 7.47 (s, 1H), 7.38 (d, *J* = 8.8 Hz, 0.3H), 7.37 (d, *J* = 8.8 Hz, 0.7H), 6.84 (s, 0.3H), 6.80 (s, 0.7H), 6.61 (s, 1.3H), 6.59 (s, 0.7H), 4.42 (dd, *J* = 12.4, 4.0 Hz, 0.3H), 4.28–4.21 (m, 2H), 4.18 (dd, *J* = 13.2, 3.2 Hz, 0.7H), 4.09 (s, 1H), 4.05 (dd, *J* = 11.2, 3.2 Hz, 2H), 4.00–3.97 (m, 2H), 3.94 (s, 2H), 3.87–3.79 (m, 3H), 3.52–3.29 (m, 5H), 2.94 (s, 1H), 2.93 (s, 2H), 2.58 (s, 1H), 2.55 (s, 2H), 2.38 (s, 3H), 2.31 (s, 6H), 2.20–2.15 (m, 2H), 2.09–2.05 (m, 1H), 1.79–1.75 (m, 2H), 1.53–1.45 (m, 2H), 1.43–1.38 (m, 3H), 1.31 (d, *J* = 6.4 Hz, 2H), 1.18 (d, *J* = 6.4 Hz, 1H).

## Supporting Information Available

X-ray collection data, HPLC-MS traces for compounds **10**–**32** (PDF)
Molecular string files of the final (CSV)

## Abbreviations Used

ABCC2: ATP-binding cassette subfamily C member 2
AUC: area under the curve
BAK: Bcl-2 homologous antagonist/killer
BAX: Bcl-2 associated X protein
Bcl-2: B-cell lymphoma 2
Bim: Bcl-2 interacting mediator of cell death
MAPK: mitogen-activated protein kinases
Mcl-1: myeloid cell leukemia 1
mTOR: murine target of rapamycin
NSCLC: non-small cell lung cancer
OATP1B1: organic anion transporter 1B1
Q_h_: clearance as percentage of liver bloodflow
QD: once a day
OATP1B1: organic anion transporter 1B1
SCLC: small cell lung cancer
