# Supplementary material for: Discovery of a Myeloid Cell Leukemia 1 (Mcl-1) Inhibitor That Demonstrates Potent In Vivo Activities in Mouse Models of Hematological and Solid Tumors
Source: J Med Chem. 2024 Aug 5;67(16):14370–93. doi: 10.1021/acs.jmedchem.4c01188 (PMC11345828; doi:10.1021/acs.jmedchem.4c01188)

Supporting Information

Discovery of a Myeloid Cell Leukemia 1 (Mcl-1) Inhibitor that Demonstrates Potent in Vivo Activities in Mouse Models of Hematological and Solid Tumors

James C. Tarr,[1] James M. Salovich,[1‡] Martin Aichinger,[2‡] KyuOk Jeon,[1,a] Nagarathanam Veerasamy,[1,b] John L. Sensintaffar,[1] Heribert Arnhof,[2] Matthias Samwer,[2] Plamen P. Christov,[3] Kwangho Kim,[3] Tobias Wunberg,[2] Norbert Schweifer,[2] Francesca Trapani,[2] Allison Arnold,[1,] Florian Martin[2], Bin Zhao,[1,c] Nagaraju Miriyala,[1] Danielle Sgubin,[1,] Stuart Fogarty,[1,] William J. Moore,[4d] Gordon M. Stott,[4] Edward T. Olejniczak,[1] Harald Engelhardt,[2] Dorothea Rudolph,[2] Taekyu Lee,[1] Darryl B. McConnell,[2] Stephen W. Fesik[1]*

1. Department of Biochemistry, Vanderbilt University School of Medicine, 2215 Garland Avenue, 607 Light Hall, Nashville, TN 37232-0146, United States.

2. Discovery Research, Boehringer Ingelheim Regional Center Vienna GmbH & Co KG, 1120 Vienna, Austria.

3. Molecular Design and Synthesis Center, Vanderbilt Institute of Chemical Biology, Vanderbilt University, Nashville, TN 37323-0146, United States.

4. Leidos Biomedical Research, Frederick National Laboratory for Cancer Research, Frederick, Maryland 21701-4907, United States.

Table of Content

S1. X-ray Collection Data and Refinement Statistics (**18**) (S3)

S2. HPLC-MS traces for compounds **10**-**32** (S4-S27)

Table S1. X-ray data collection and refinement statistics for compound.

Compound	VU0817431
PDB Accession code	
Space Group	P21 21 2
Cell Dimensions	
a, b, c (Å)	99.453, 136.858, 38.624
α, β, γ (°)	90.00, 90.00, 90.00
Resolution (Å)	38.65-1.90 (1.93-1.90)
R_{merge} (%)	0.142 (0.613)
Mean I / σI	10.4 (1.4)
Completeness (%)	96.5 (96.7)
Redundancy	5.8 (4.1)
No. Reflections	39,167
R_{work}/R_{free}	0.1773/0.2103
R.m.s. deviations	
Bond lengths	0.012
Bond angles	1.819
Preferred regions (%)	97.67
Allowed regions (%)	1.94
Disallowed regions (%)	0.39

High resolution shells are in parentheses.

S2. LCMS Traces of Compounds 10-32

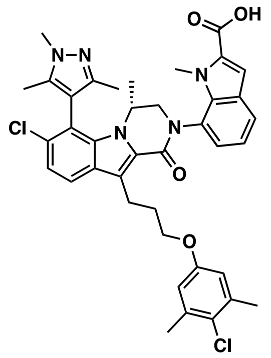

(*R*)-7-(7-Chloro-10-(3-(4-chloro-3,5-dimethylphenoxy)propyl)-4-methyl-1-oxo-6-(1,3,5-trimethyl-1*H*-pyrazol-4-yl)-3,4-dihydropyrazino[1,2-*a*]indol-2(1*H*)-yl)-1-methyl-1*H*-indole-2-carboxylic acid **(Compound 10)**

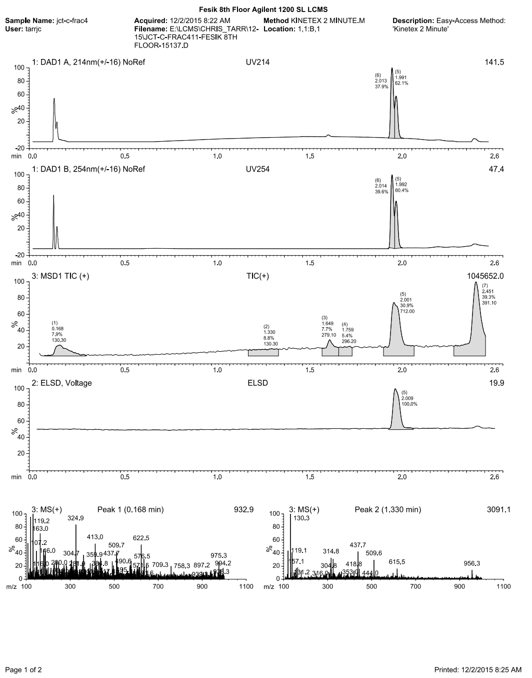

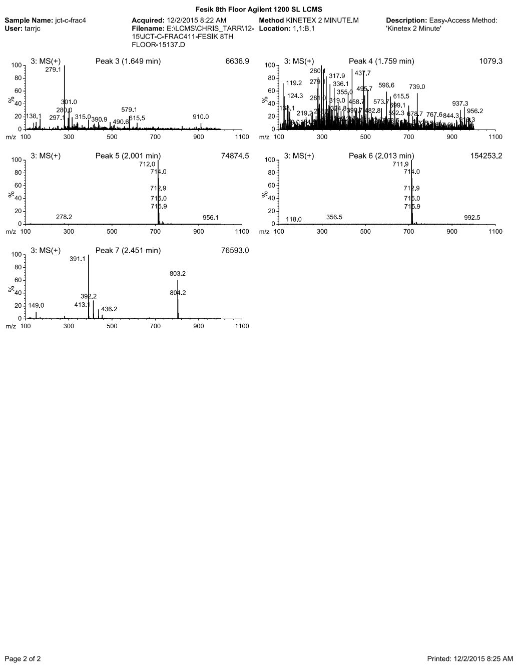

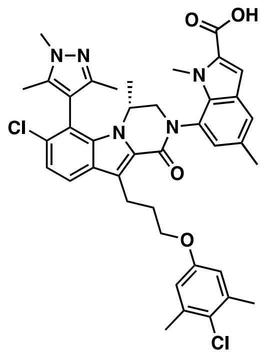

(*R*)-7-(7-chloro-10-(3-(4-chloro-3,5-dimethylphenoxy)propyl)-4-methyl-1-oxo-6-(1,3,5-trimethyl-1*H*-pyrazol-4-yl)-3,4-dihydropyrazino[1,2-*a*]indol-2(1*H*)-yl)-1,5-dimethyl-1*H*-indole-2-carboxylic acid **(Compound 11)**

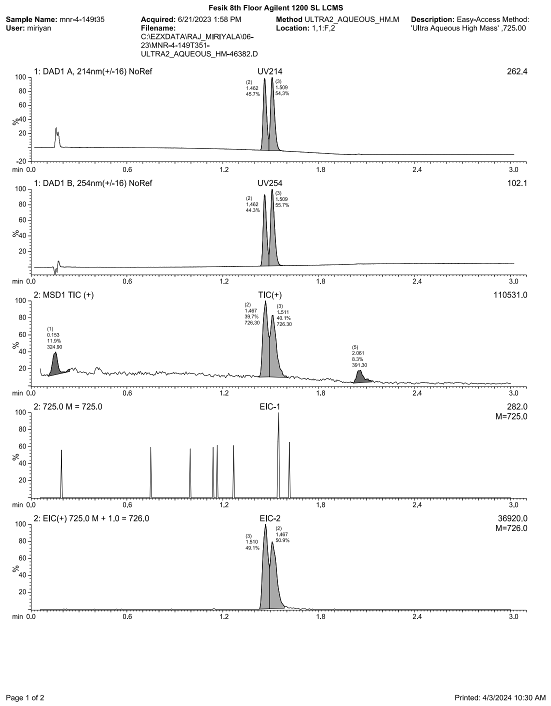

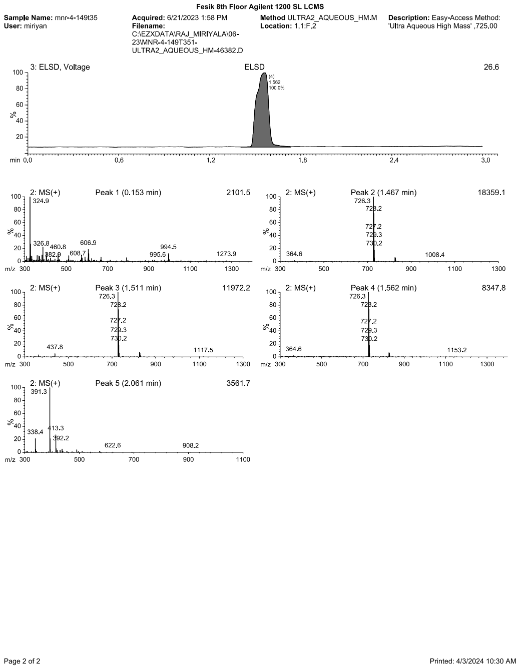

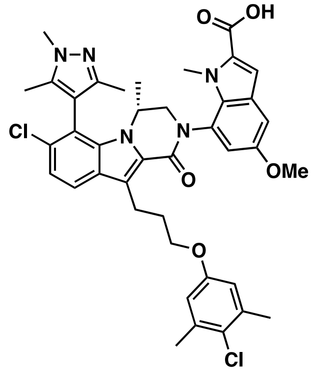

(*R*)-7-(7-Chloro-10-(3-(4-chloro-3,5-dimethylphenoxy)propyl)-4-methyl-1-oxo-6-(1,3,5-trimethyl-1*H*-pyrazol-4-yl)-3,4-dihydropyrazino[1,2-*a*]indol-2(1*H*)-yl)-5-methoxy-1-methyl-1*H*-indole-2-carboxylic acid **(Compound 12)**

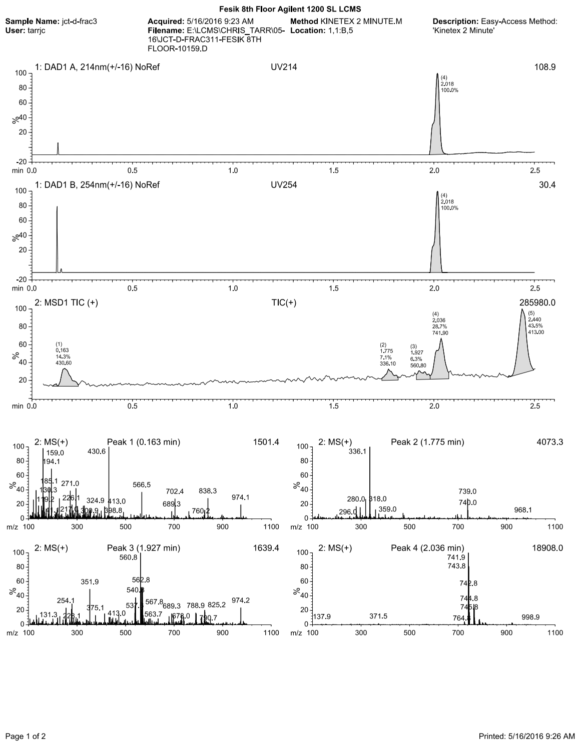

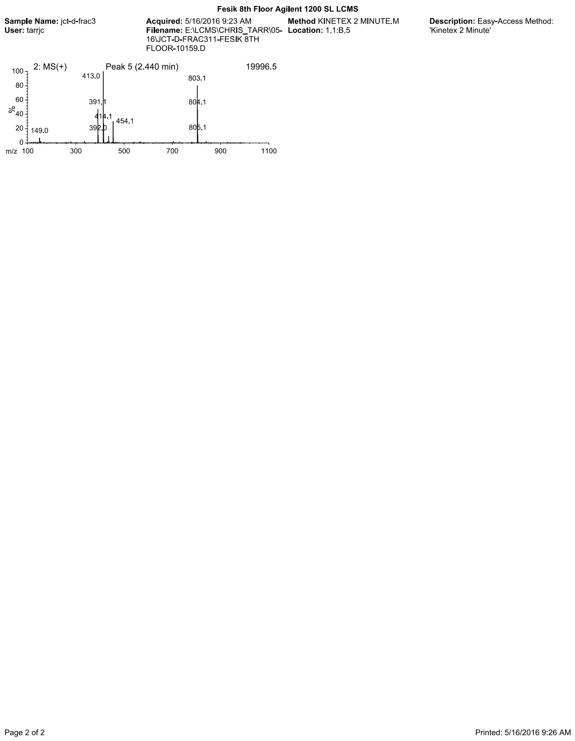

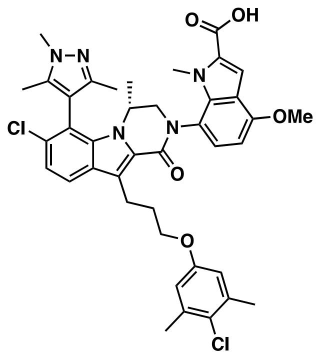

(*R*)-7-(7-Chloro-10-(3-(4-chloro-3,5-dimethylphenoxy)propyl)-4-methyl-1-oxo-6-(1,3,5-trimethyl-1*H*-pyrazol-4-yl)-3,4-dihydropyrazino[1,2-*a*]indol-2(1*H*)-yl)-4-methoxy-1-methyl-1*H*-indole-2-carboxylic acid **(Compound 13)**

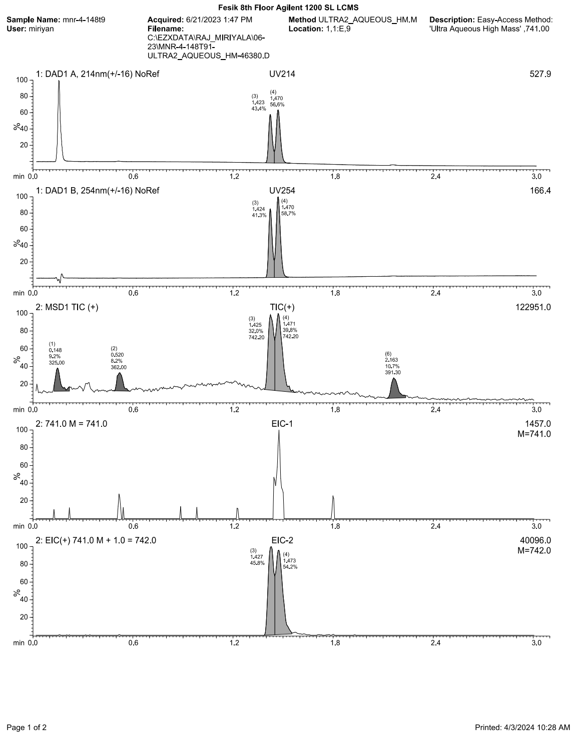

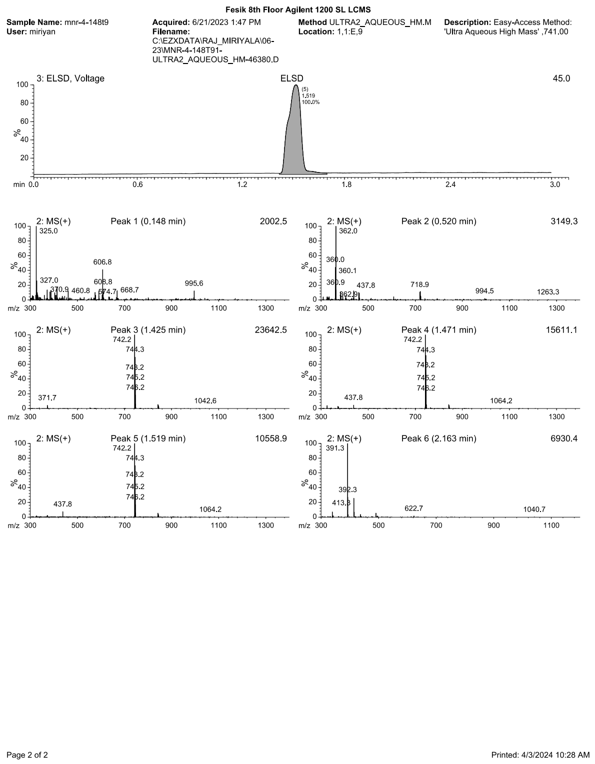

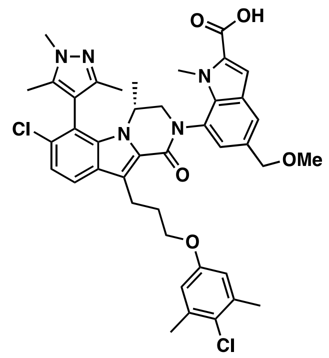

(*R*)-7-(7-chloro-10-(3-(4-chloro-3,5-dimethylphenoxy)propyl)-4-methyl-1-oxo-6-(1,3,5-trimethyl-1*H*-pyrazol-4-yl)-3,4-dihydropyrazino[1,2-*a*]indol-2(1*H*)-yl)-5-(methoxymethyl)-1-methyl-1*H*-indole-2-carboxylic acid **(Compound 14)**

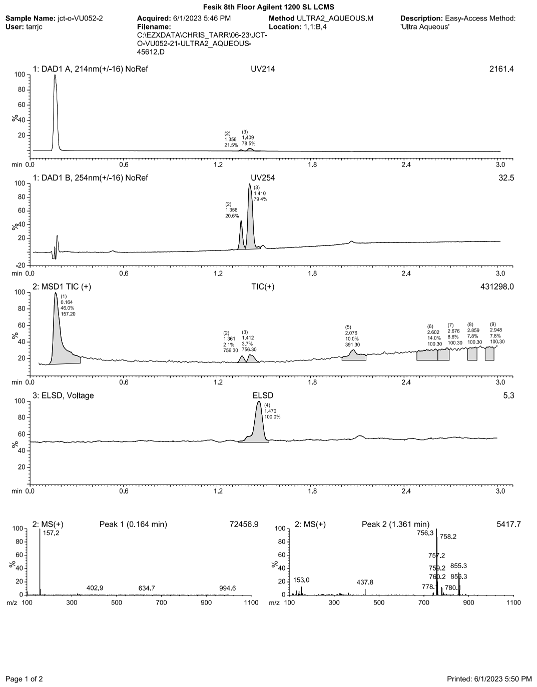

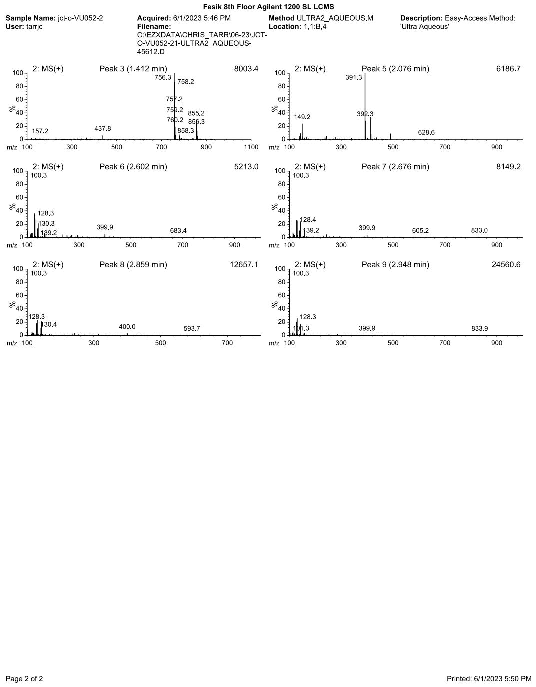

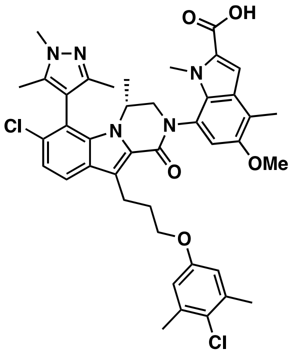

(*R*)-7-(7-chloro-10-(3-(4-chloro-3,5-dimethylphenoxy)propyl)-4-methyl-1-oxo-6-(1,3,5-trimethyl-1*H*-pyrazol-4-yl)-3,4-dihydropyrazino[1,2-*a*]indol-2(1*H*)-yl)-5-methoxy-1,4-dimethyl-1*H*-indole-2-carboxylic acid **(Compound 15)**

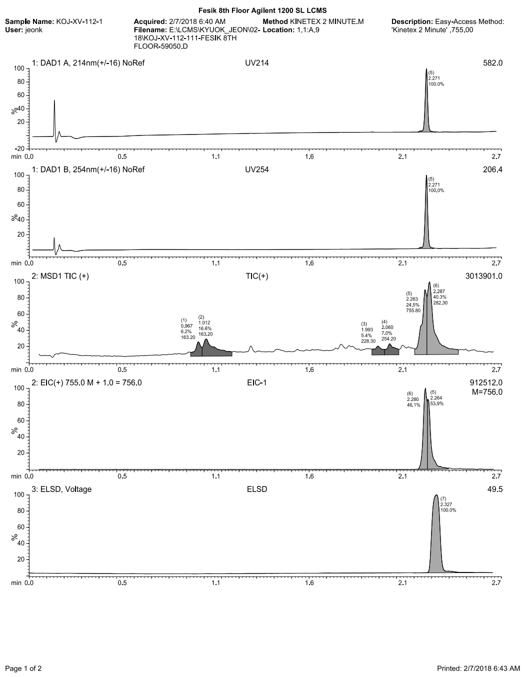

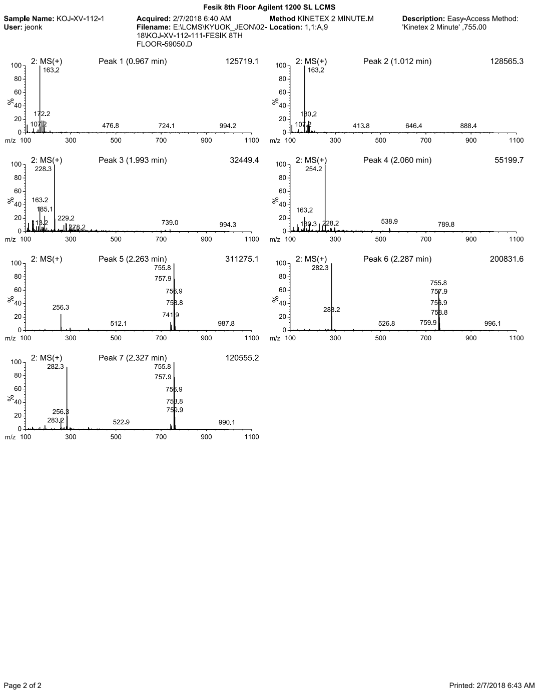

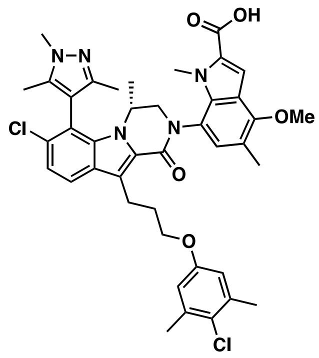

(*R*)-7-(7-chloro-10-(3-(4-chloro-3,5-dimethylphenoxy)propyl)-4-methyl-1-oxo-6-(1,3,5-trimethyl-1*H*-pyrazol-4-yl)-3,4-dihydropyrazino[1,2-*a*]indol-2(1*H*)-yl)-4-methoxy-1,5-dimethyl-1*H*-indole-2-carboxylic acid **(Compound 16)**

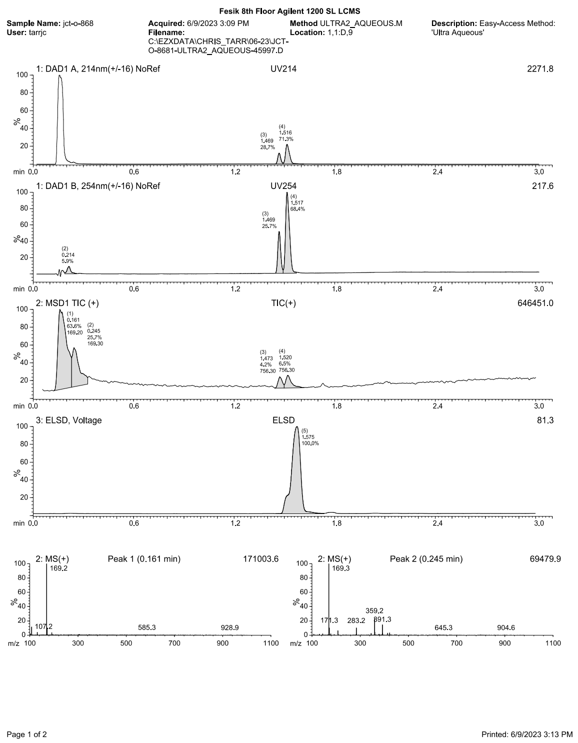

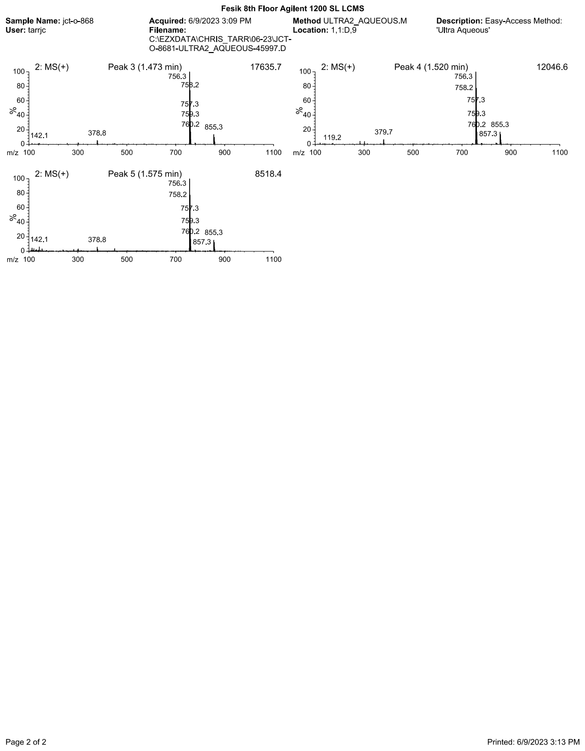

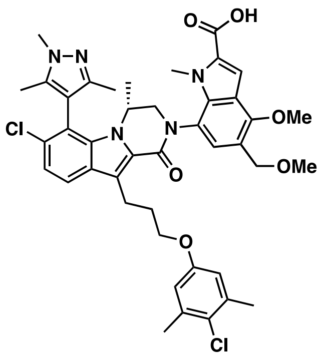

(*R*)-7-(7-chloro-10-(3-(4-chloro-3,5-dimethylphenoxy)propyl)-4-methyl-1-oxo-6-(1,3,5-trimethyl-1*H*-pyrazol-4-yl)-3,4-dihydropyrazino[1,2-*a*]indol-2(1*H*)-yl)-4-methoxy-5-(methoxymethyl)-1-methyl-1*H*-indole-2-carboxylic acid **(Compound 17)**

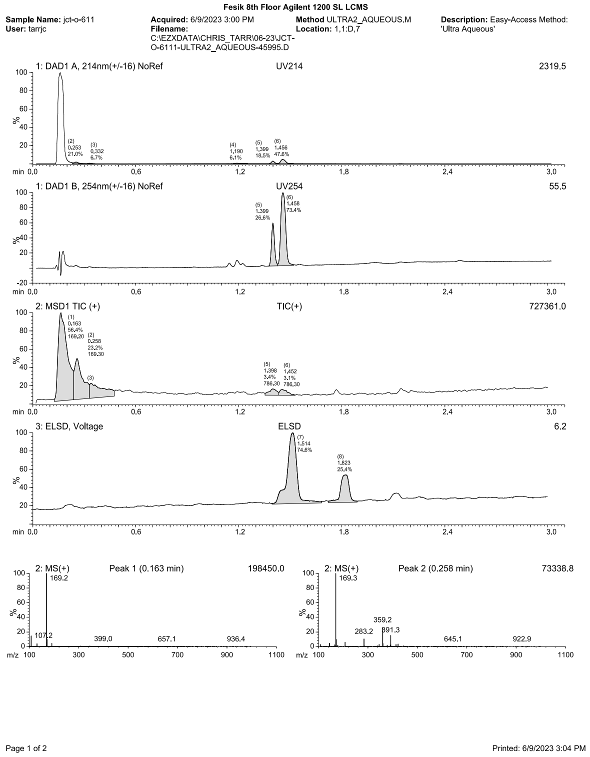

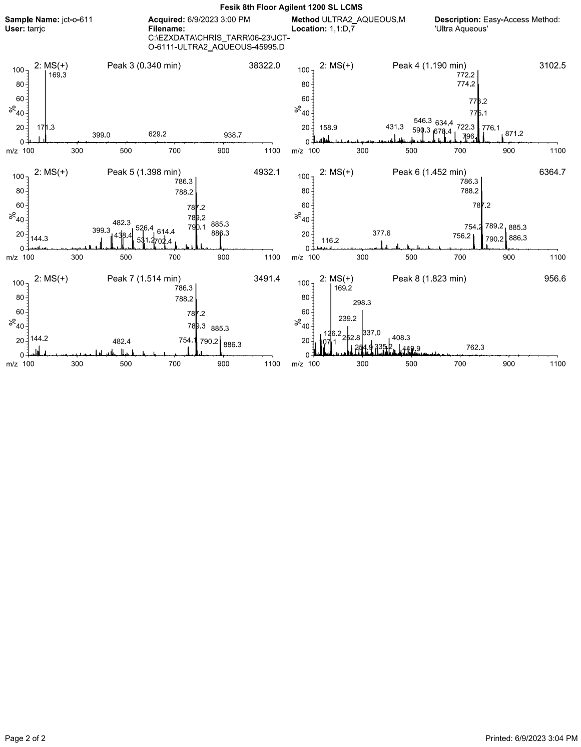

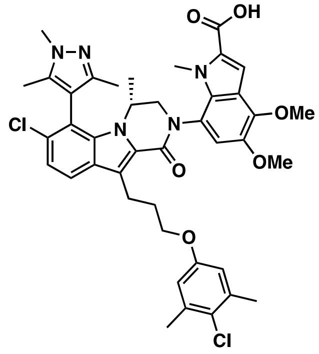

(*R*)-7-(7-chloro-10-(3-(4-chloro-3,5-dimethylphenoxy)propyl)-4-methyl-1-oxo-6-(1,3,5-trimethyl-1*H*-pyrazol-4-yl)-3,4-dihydropyrazino[1,2-*a*]indol-2(1*H*)-yl)-4,5-dimethoxy-1-methyl-1*H*-indole-2-carboxylic acid **(Compound 18)**

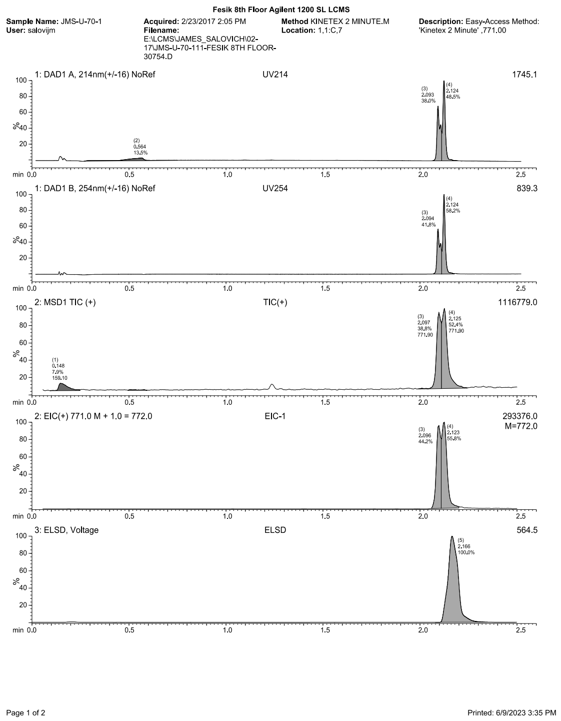

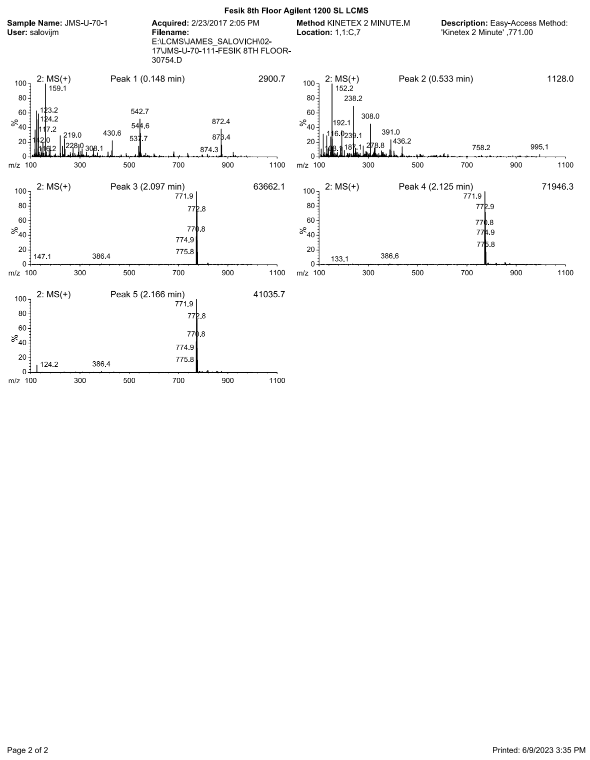

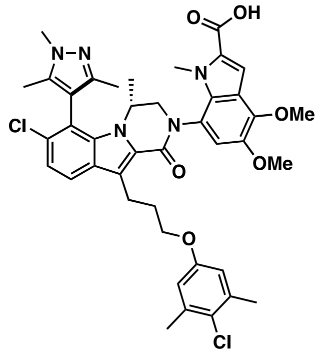

(*M*,*R*)-7-(7-chloro-10-(3-(4-chloro-3,5-dimethylphenoxy)propyl)-4-methyl-1-oxo-6-(1,3,5-trimethyl-1*H*-pyrazol-4-yl)-3,4-dihydropyrazino[1,2-*a*]indol-2(1*H*)-yl)-4,5-dimethoxy-1-methyl-1*H*-indole-2-carboxylic acid **(Compound *M*-18)**

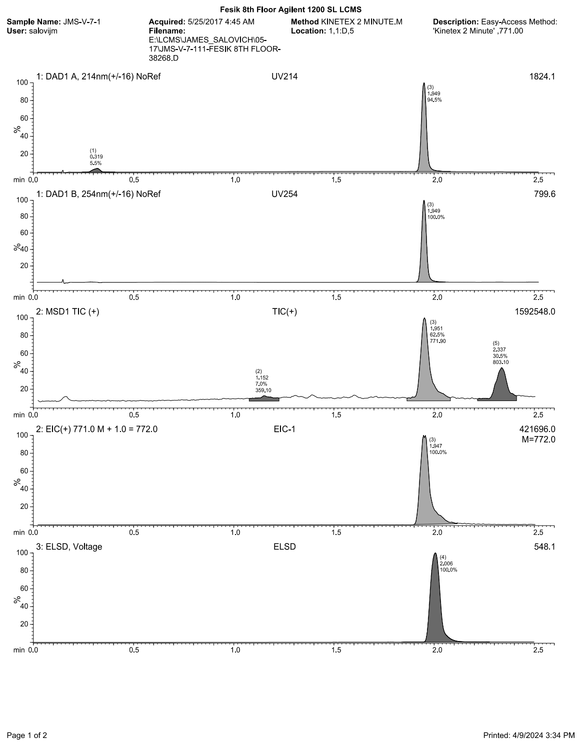

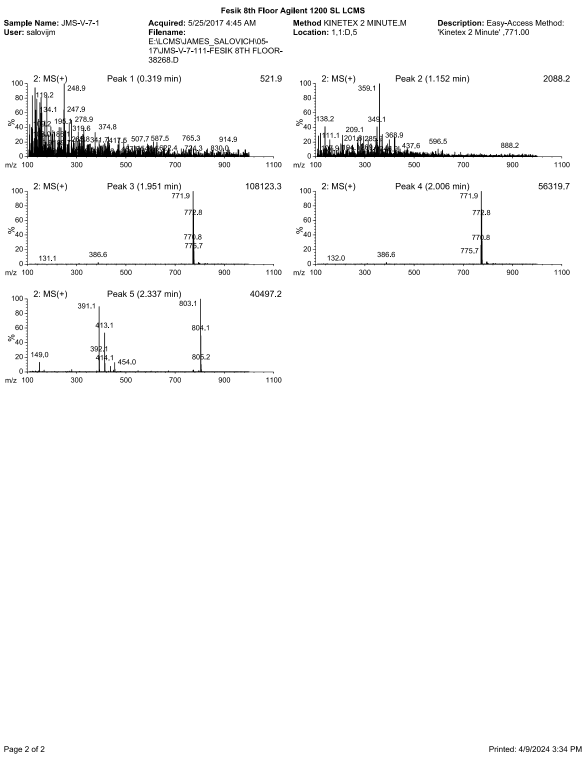

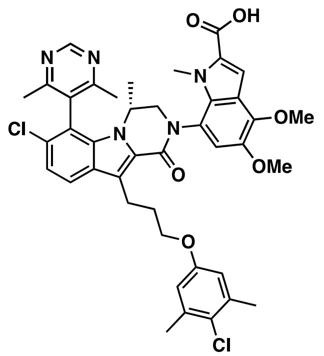

(*R*)-7-(7-chloro-10-(3-(4-chloro-3,5-dimethylphenoxy)propyl)-6-(4,6-dimethylpyrimidin-5-yl)-4-methyl-1-oxo-3,4-dihydropyrazino[1,2-*a*]indol-2(1*H*)-yl)-4,5-dimethoxy-1-methyl-1*H*-indole-2-carboxylic acid **(Compound 19)**

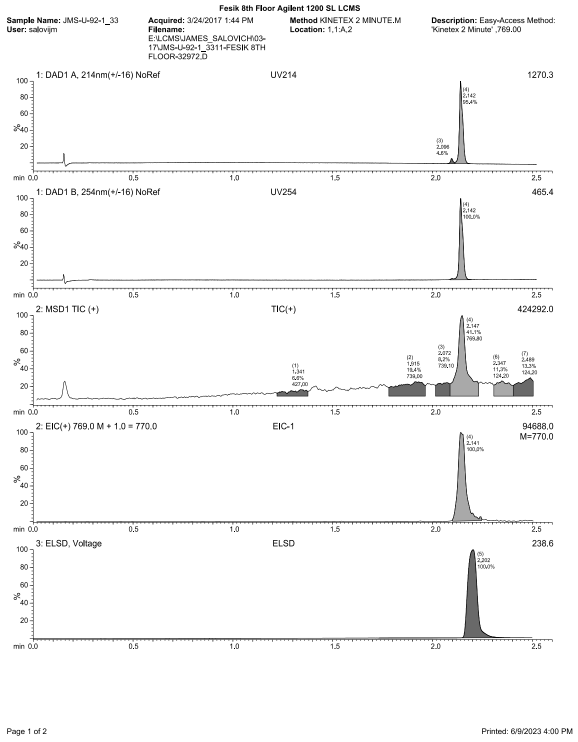

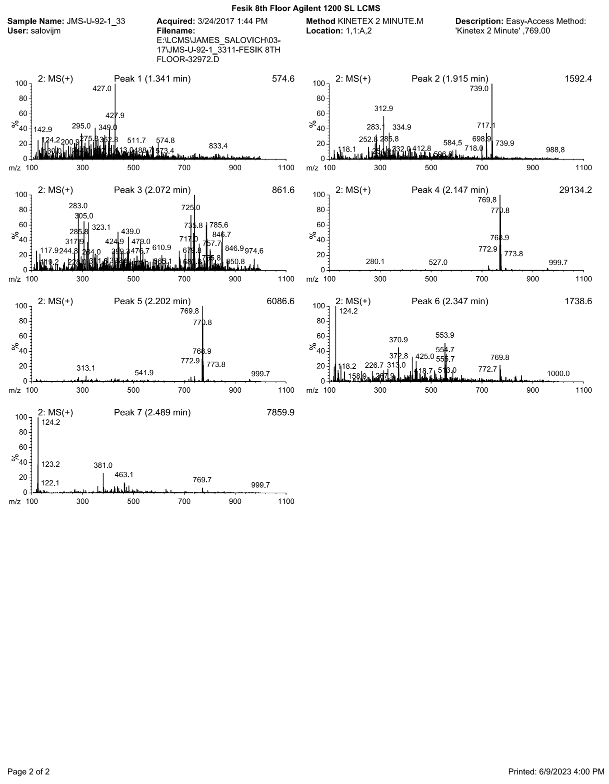

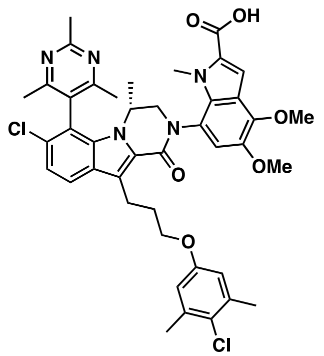

(*R*)-7-(7-chloro-10-(3-(4-chloro-3,5-dimethylphenoxy)propyl)-4-methyl-1-oxo-6-(2,4,6-trimethylpyrimidin-5-yl)-3,4-dihydropyrazino[1,2-*a*]indol-2(1*H*)-yl)-4,5-dimethoxy-1-methyl-1*H*-indole-2-carboxylic acid **(Compound 20)**

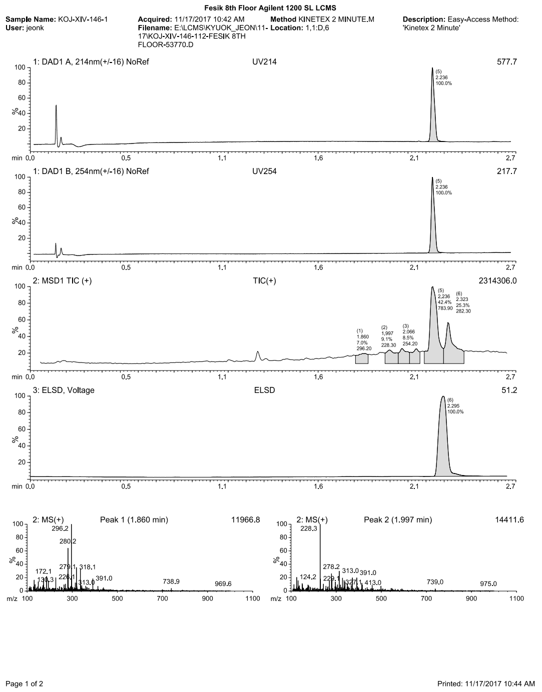

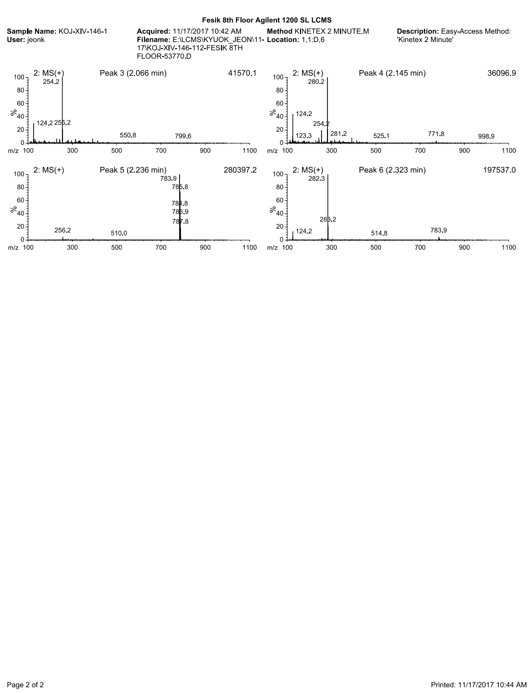

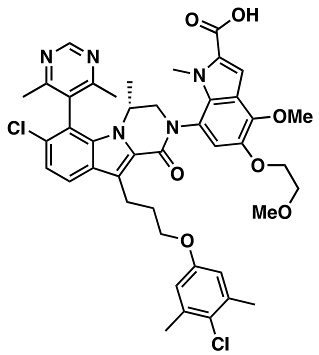

(*R*)-7-(7-chloro-10-(3-(4-chloro-3,5-dimethylphenoxy)propyl)-6-(4,6-dimethylpyrimidin-5-yl)-4-methyl-1-oxo-3,4-dihydropyrazino[1,2-*a*]indol-2(1*H*)-yl)-4-methoxy-5-(2-methoxyethoxy)-1-methyl-1*H*-indole-2-carboxylic acid **(Compound 21)**

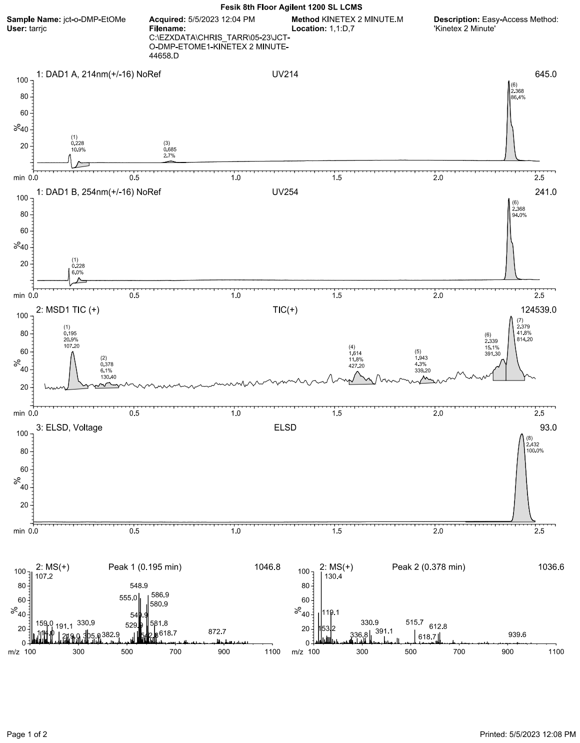

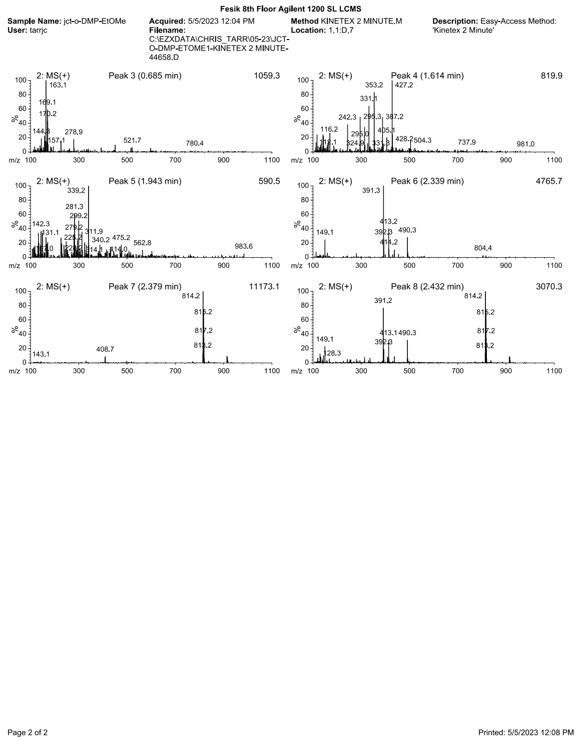

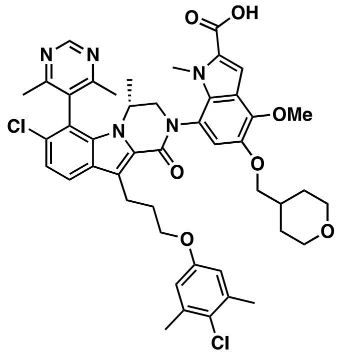

(*R*)-7-(7-chloro-10-(3-(4-chloro-3,5-dimethylphenoxy)propyl)-6-(4,6-dimethylpyrimidin-5-yl)-4-methyl-1-oxo-3,4-dihydropyrazino[1,2-*a*]indol-2(1*H*)-yl)-4-methoxy-1-methyl-5-((tetrahydro-2*H*-pyran-4-yl)methoxy)-1*H*-indole-2-carboxylic acid **(Compound 22)**

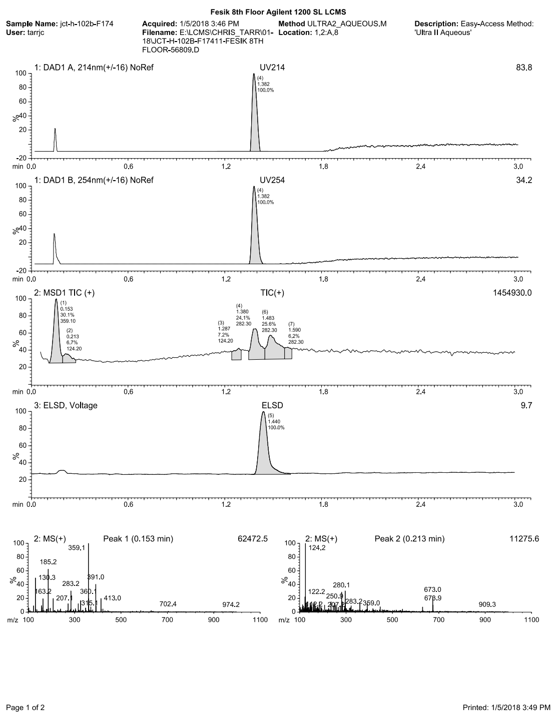

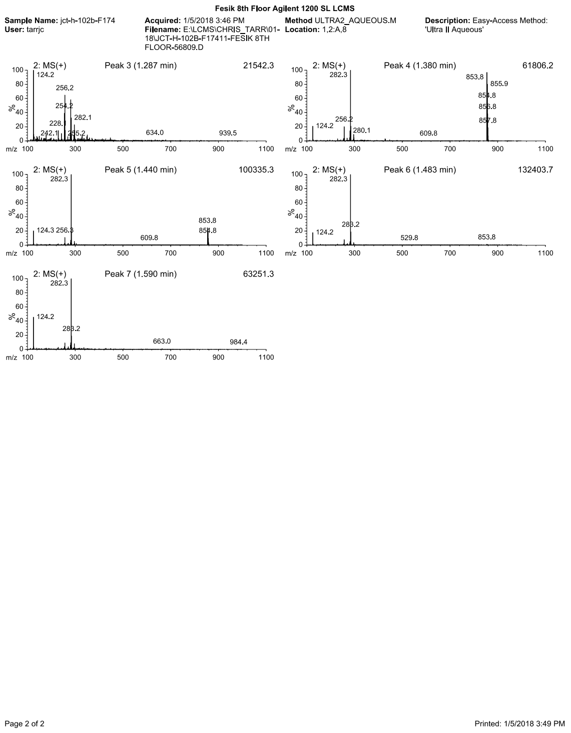

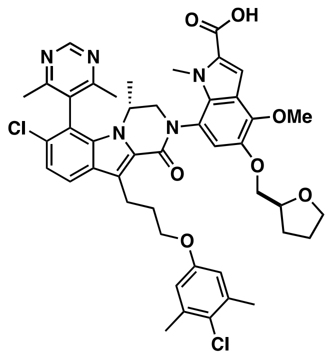

7-((*R*)-7-chloro-10-(3-(4-chloro-3,5-dimethylphenoxy)propyl)-6-(4,6-dimethylpyrimidin-5-yl)-4-methyl-1-oxo-3,4-dihydropyrazino[1,2-*a*]indol-2(1*H*)-yl)-4-methoxy-1-methyl-5-(((*S*)-tetrahydrofuran-2-yl)methoxy)-1*H*-indole-2-carboxylic acid **(Compound 23)**

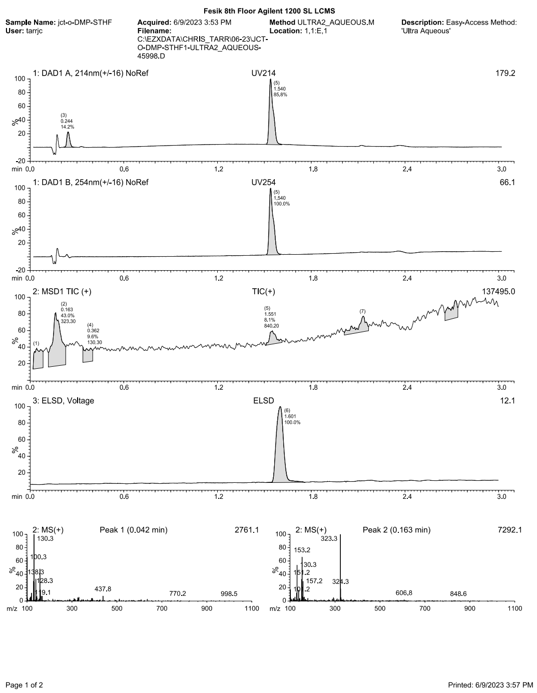

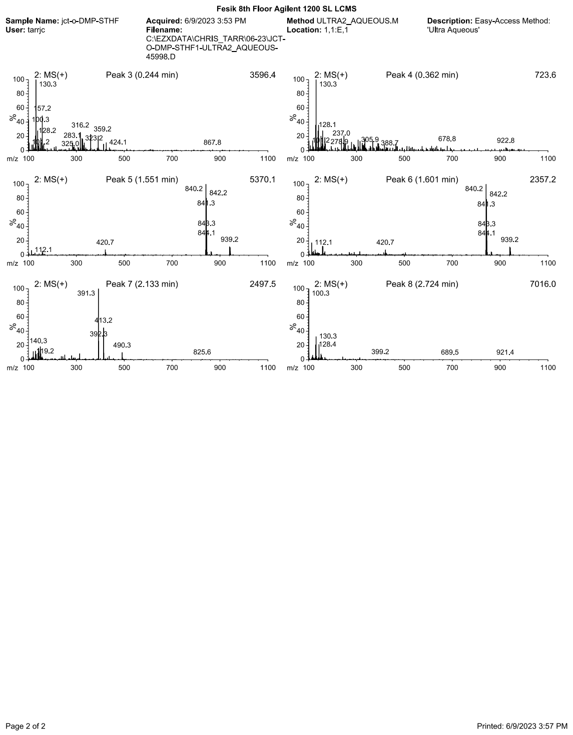

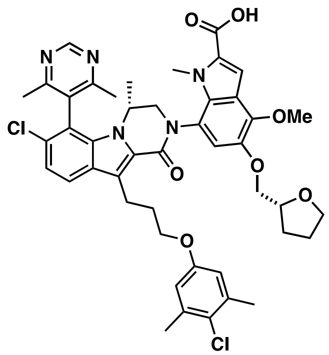

7-((*R*)-7-chloro-10-(3-(4-chloro-3,5-dimethylphenoxy)propyl)-6-(4,6-dimethylpyrimidin-5-yl)-4-methyl-1-oxo-3,4-dihydropyrazino[1,2-*a*]indol-2(1*H*)-yl)-4-methoxy-1-methyl-5-(((*R*)-tetrahydrofuran-2-yl)methoxy)-1*H*-indole-2-carboxylic acid **(Compound 24)**

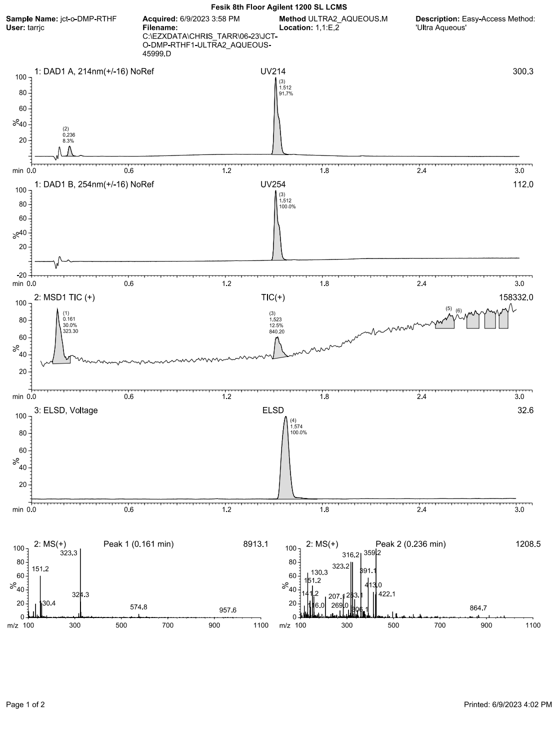

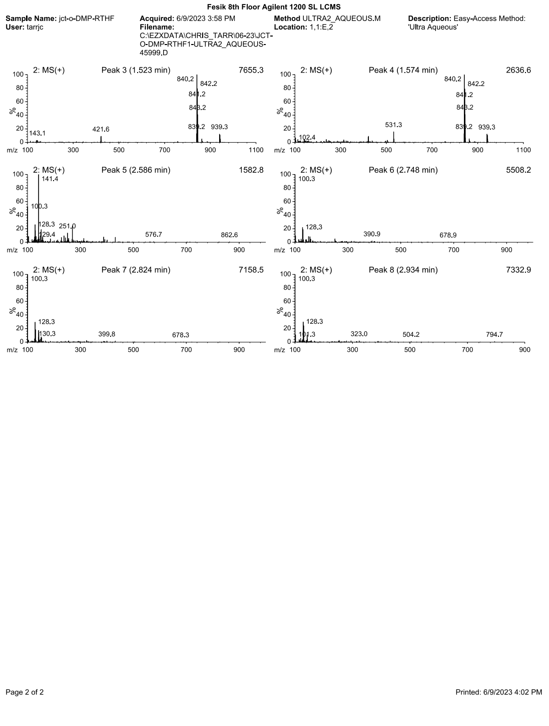

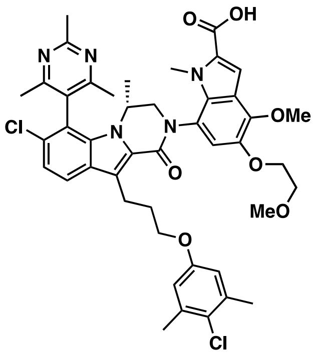

(*R*)-7-(7-chloro-10-(3-(4-chloro-3,5-dimethylphenoxy)propyl)-4-methyl-1-oxo-6-(2,4,6-trimethylpyrimidin-5-yl)-3,4-dihydropyrazino[1,2-*a*]indol-2(1*H*)-yl)-4-methoxy-5-(2-methoxyethoxy)-1-methyl-1*H*-indole-2-carboxylic acid **(Compound 25)**

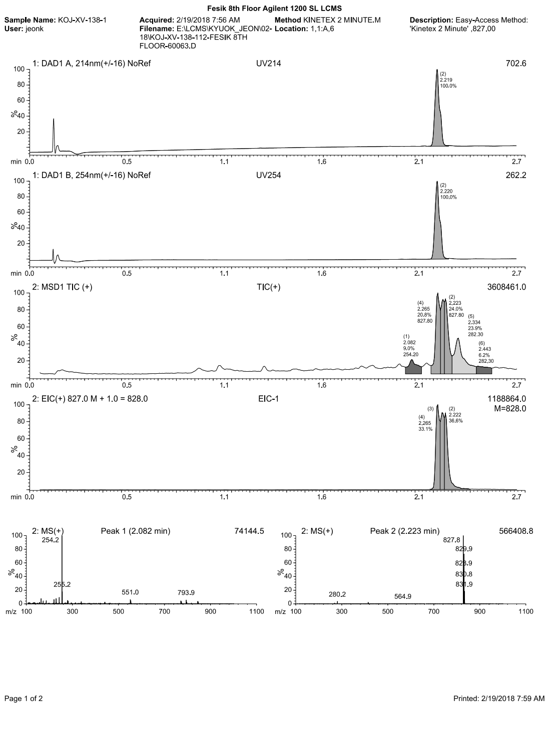

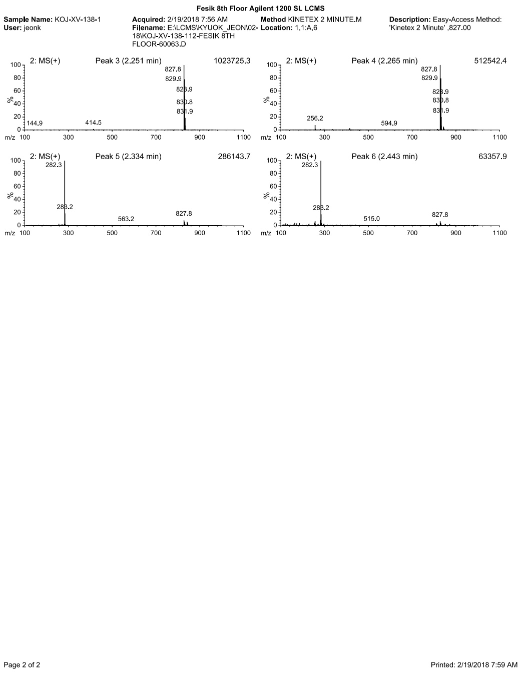

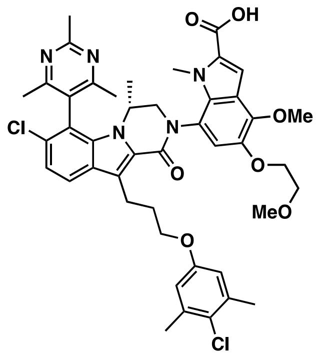

(*R*)-7-(7-chloro-10-(3-(4-chloro-3,5-dimethylphenoxy)propyl)-4-methyl-1-oxo-6-(2,4,6-trimethylpyrimidin-5-yl)-3,4-dihydropyrazino[1,2-*a*]indol-2(1*H*)-yl)-4-methoxy-1-methyl-5-((tetrahydro-2*H*-pyran-4-yl)methoxy)-1*H*-indole-2-carboxylic acid **(Compound 26)**

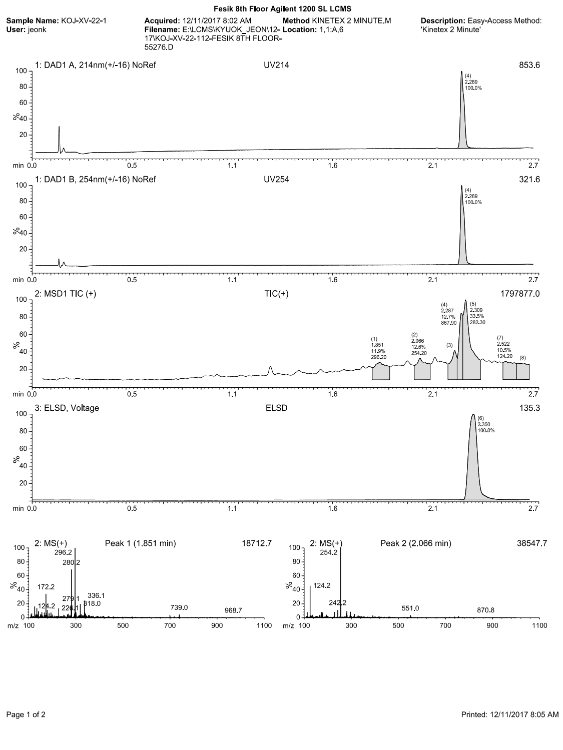

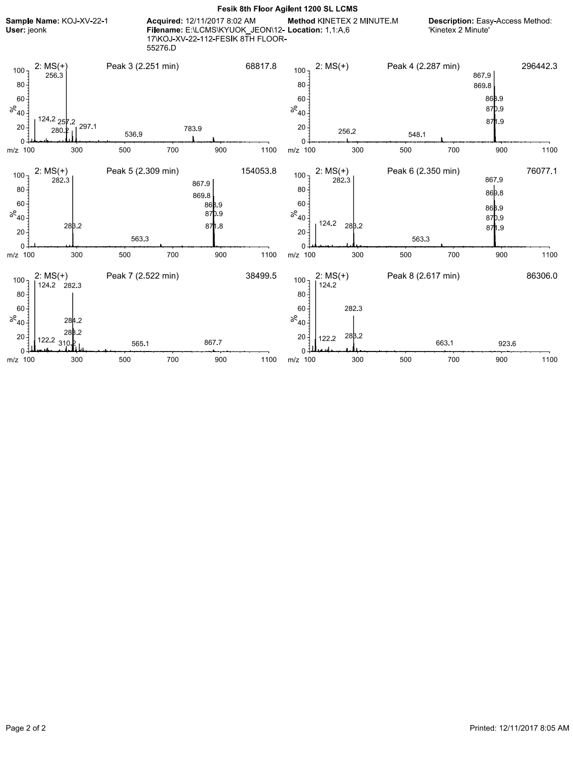

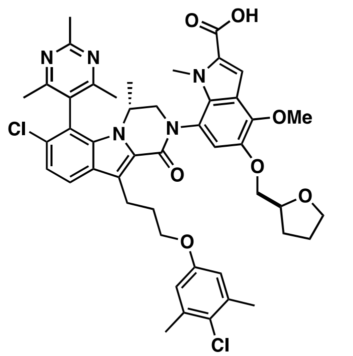

7-((*R*)-7-chloro-10-(3-(4-chloro-3,5-dimethylphenoxy)propyl)-6-(2,4,6-trimethylpyrimidin-5-yl)-4-methyl-1-oxo-3,4-dihydropyrazino[1,2-*a*]indol-2(1*H*)-yl)-4-methoxy-1-methyl-5-(((*S*)-tetrahydrofuran-2-yl)methoxy)-1*H*-indole-2-carboxylic acid **(Compound 27)**

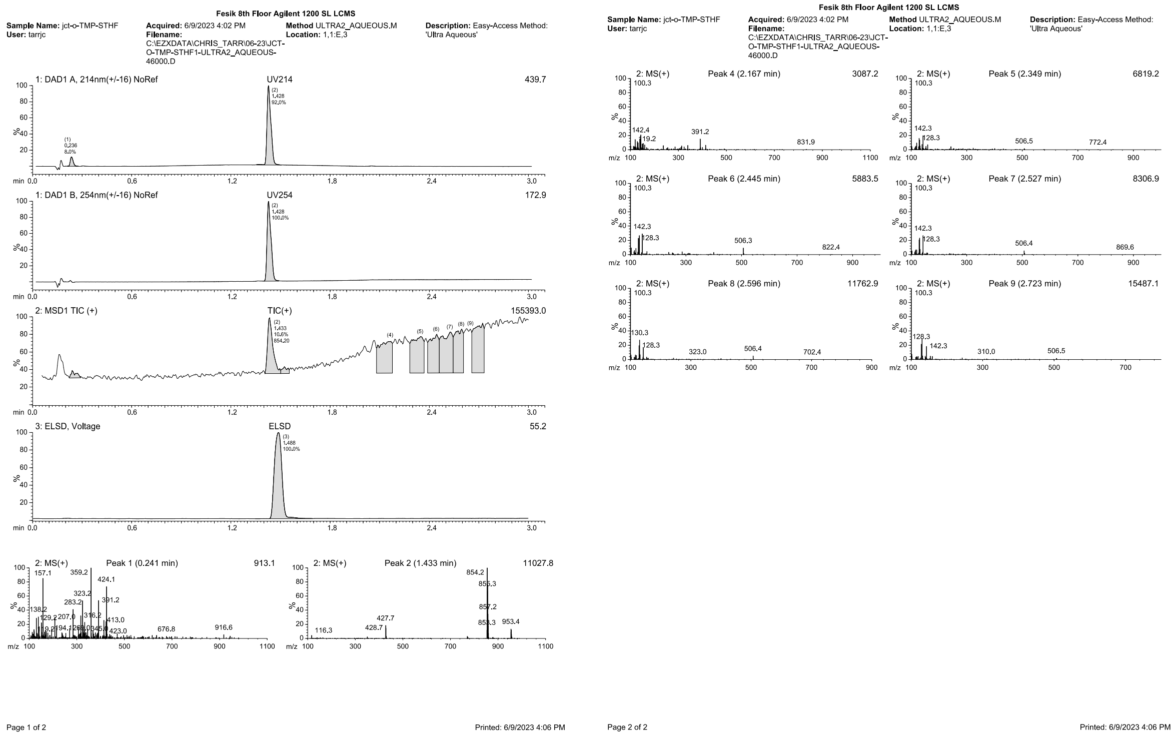

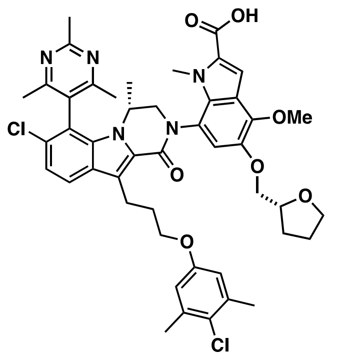

7-((*R*)-7-chloro-10-(3-(4-chloro-3,5-dimethylphenoxy)propyl)-6-(2,4,6-trimethylpyrimidin-5-yl)-4-methyl-1-oxo-3,4-dihydropyrazino[1,2-*a*]indol-2(1*H*)-yl)-4-methoxy-1-methyl-5-(((*R*)-tetrahydrofuran-2-yl)methoxy)-1*H*-indole-2-carboxylic acid **(Compound 28**)

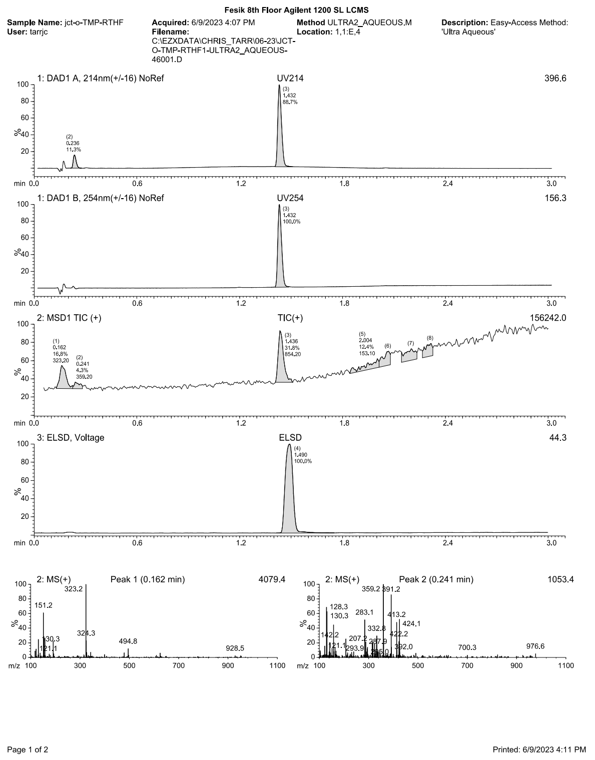

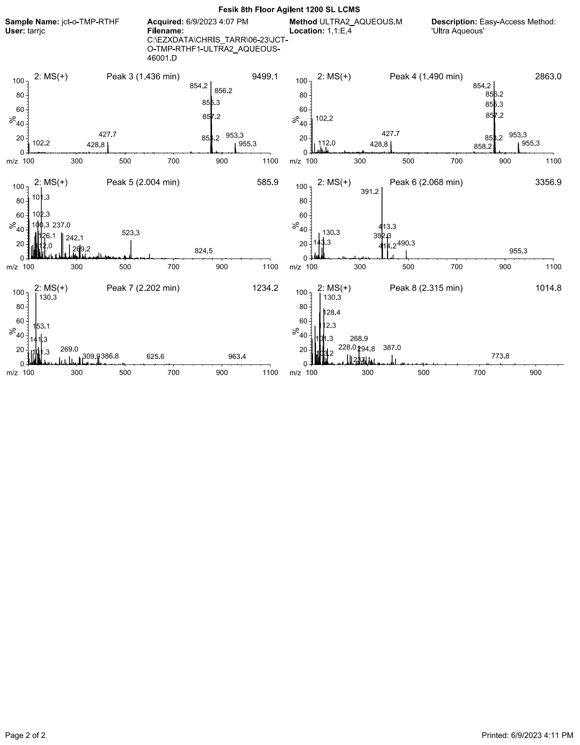

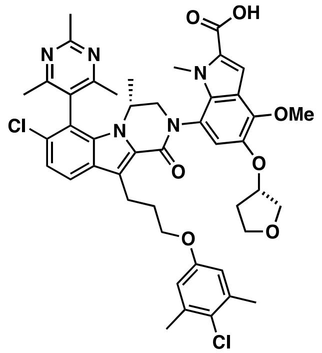

7-((*R*)-7-chloro-10-(3-(4-chloro-3,5-dimethylphenoxy)propyl)-4-methyl-1-oxo-6-(2,4,6-trimethylpyrimidin-5-yl)-3,4-dihydropyrazino[1,2-*a*]indol-2(1*H*)-yl)-4-methoxy-1-methyl-5-(((*S*)-tetrahydrofuran-3-yl)oxy)-1*H*-indole-2-carboxylic acid **(Compound 29)**

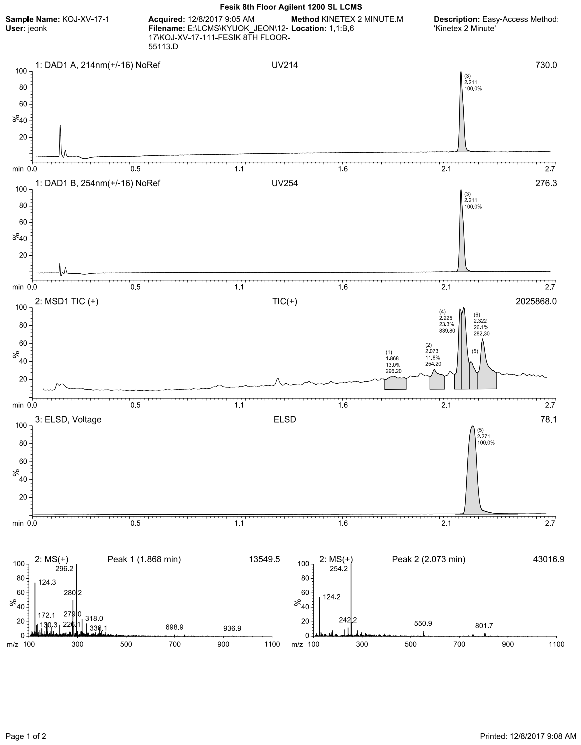

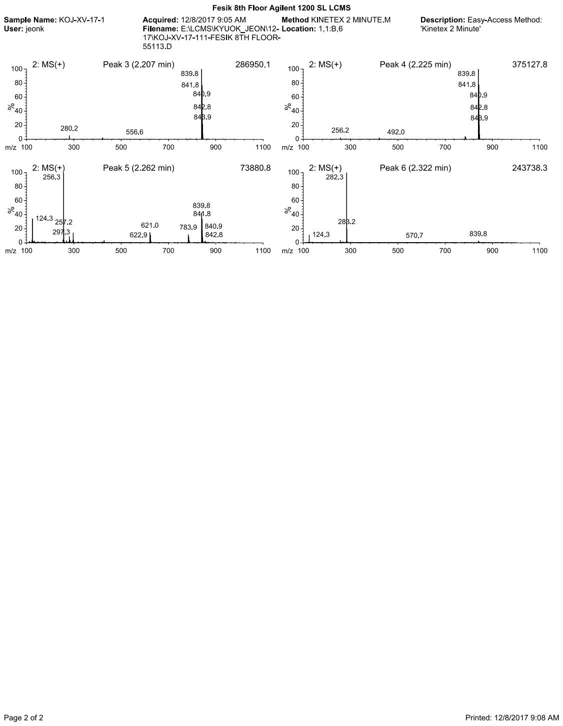

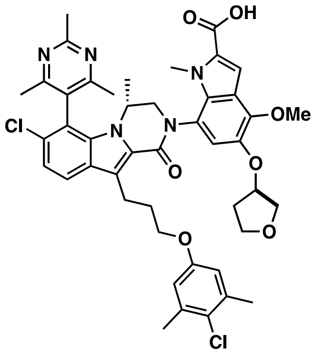

7-((*R*)-7-chloro-10-(3-(4-chloro-3,5-dimethylphenoxy)propyl)-4-methyl-1-oxo-6-(2,4,6-trimethylpyrimidin-5-yl)-3,4-dihydropyrazino[1,2-*a*]indol-2(1*H*)-yl)-4-methoxy-1-methyl-5-(((*R*)-tetrahydrofuran-3-yl)oxy)-1*H*-indole-2-carboxylic acid **(Compound 30)**

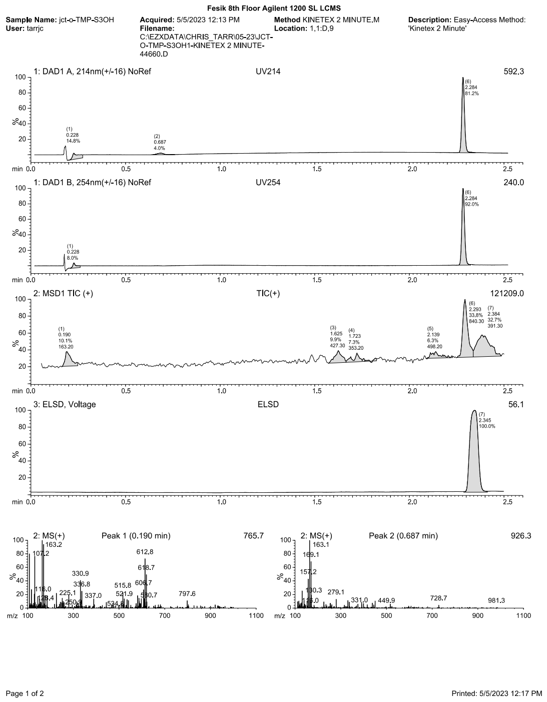

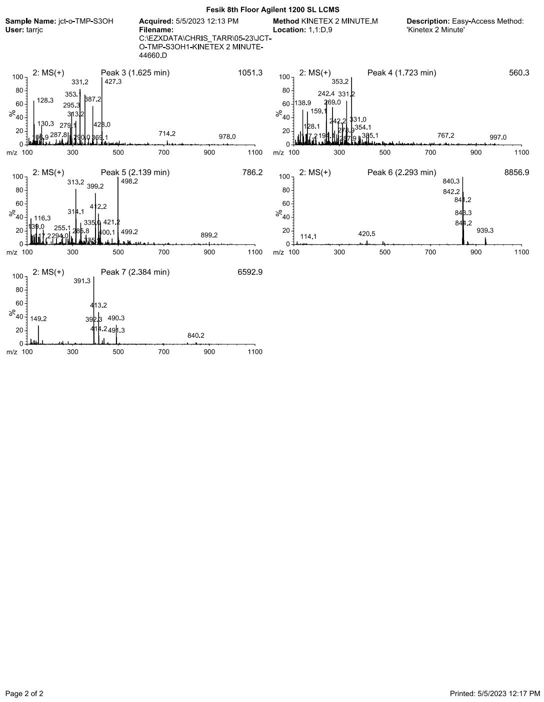

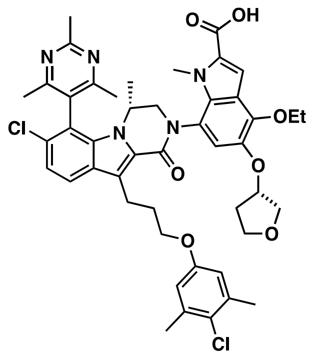

7-((*R*)-7-chloro-10-(3-(4-chloro-3,5-dimethylphenoxy)propyl)-4-methyl-1-oxo-6-(2,4,6-trimethylpyrimidin-5-yl)-3,4-dihydropyrazino[1,2-*a*]indol-2(1*H*)-yl)-4-ethoxy-1-methyl-5-(((*S*)-tetrahydrofuran-3-yl)oxy)-1*H*-indole-2-carboxylic acid **(Compound 31)**

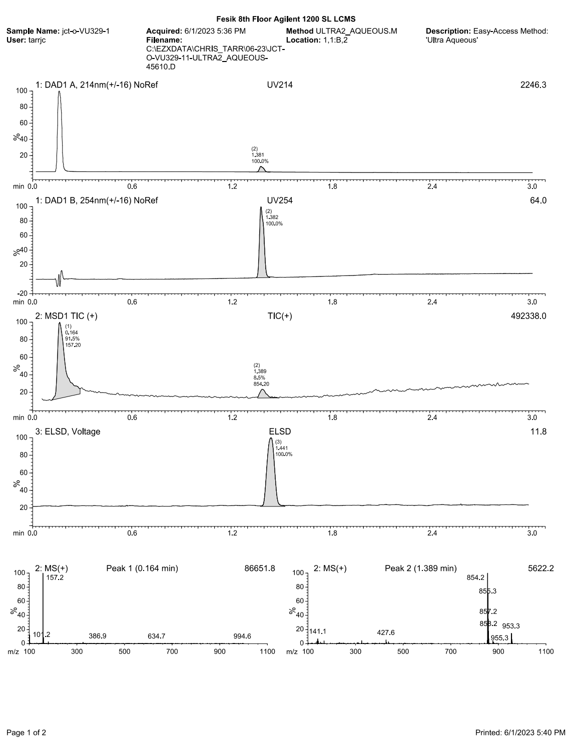

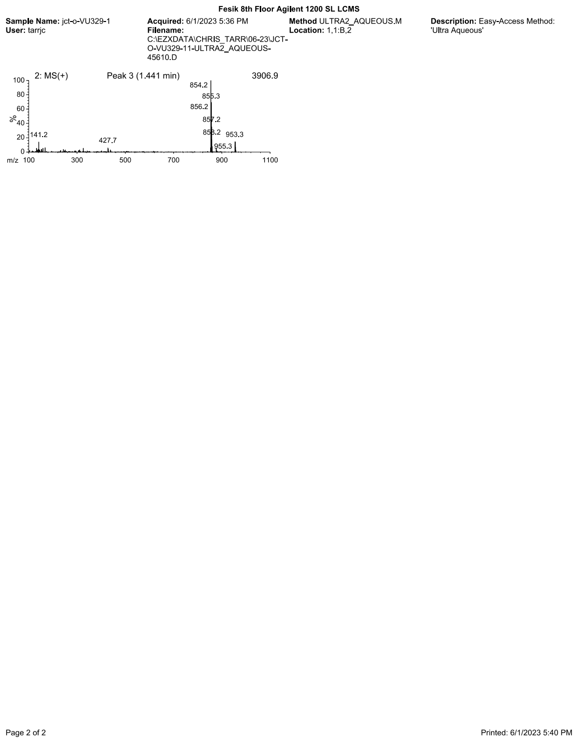

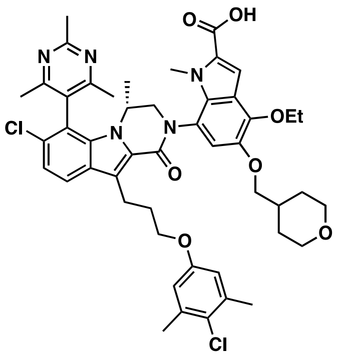

(*R*)-7-(7-chloro-10-(3-(4-chloro-3,5-dimethylphenoxy)propyl)-4-methyl-1-oxo-6-(2,4,6-trimethylpyrimidin-5-yl)-3,4-dihydropyrazino[1,2-*a*]indol-2(1*H*)-yl)-4-ethoxy-1-methyl-5-((tetrahydro-2*H*-pyran-4-yl)methoxy)-1*H*-indole-2-carboxylic acid **(Compound 32)**

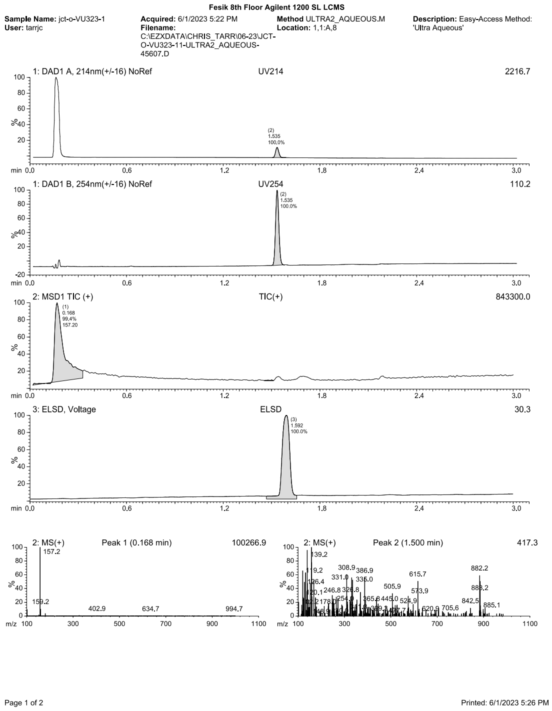

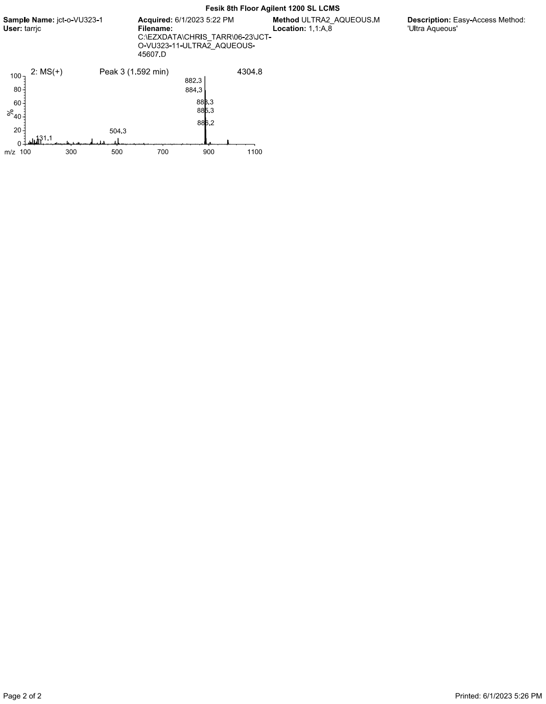

Supplement: Supplementary file 1 — jm4c01188_si_001.pdf [file jm4c01188_si_001.pdf]
